# Direct RNA Nanopore Sequencing of Pseudomonas aeruginosa Clone C Transcriptomes

**DOI:** 10.1128/JB.00418-21

**Published:** 2022-01-18

**Authors:** Marie-Madlen Pust, Colin Francis Davenport, Lutz Wiehlmann, Burkhard Tümmler

**Affiliations:** a Department of Pediatric Pneumology, Allergology, and Neonatology, Hannover Medical School, Hannover, Germany; b Biomedical Research in Endstage and Obstructive Lung Disease Hannover (BREATH), German Center for Lung Research, Hannover Medical School, Hannover, Germany; c Research Core Unit Genomics, Hannover Medical School, Hannover, Germany; University of California San Francisco

**Keywords:** *Pseudomonas aeruginosa*, antisense RNA, direct RNA sequencing, Nanopore sequencing, tRNA, transcriptome

## Abstract

The transcriptomes of Pseudomonas aeruginosa clone C isolates NN2 and SG17M during the mid-exponential and early stationary phases of planktonic growth were evaluated by direct RNA sequencing on the nanopore platform and compared with established short-read cDNA sequencing on the Illumina platform. Fifty to ninety percent of the sense RNAs turned out to be rRNA molecules, followed by similar proportions of mRNA transcripts and noncoding RNAs. The two platforms detected similar proportions of uncharged tRNAs and 29 yet-undescribed antisense tRNAs. For example, the rarest arginine codon was paired with the most abundant tRNA^Arg^, and the tRNA^Arg^ gene is missing for the most frequent arginine codon. More than 90% of the antisense RNA molecules were complementary to a coding sequence. The antisense RNAs were evenly distributed in the genomes. Direct RNA sequencing identified more than 4,000 distinct nonoverlapping antisense RNAs during exponential and stationary growth. Besides highly expressed small antisense RNAs less than 200 bases in size, a population of longer antisense RNAs was sequenced that covered a broad range (a few hundred to thousands of bases) and could be complementary to a contig of several genes. In summary, direct RNA sequencing identified yet-undescribed RNA molecules and an unexpected composition of the pools of tRNAs and sense and antisense RNAs.

**IMPORTANCE** Genome-wide gene expression of bacteria is commonly studied by high-throughput sequencing of size-selected cDNA fragment libraries of reverse-transcribed RNA preparations. However, the depletion of rRNAs, enzymatic reverse transcription, and the fragmentation, size selection, and amplification during library preparation lead to inevitable losses of information about the initial composition of the RNA pool. We demonstrate that direct RNA sequencing on the Nanopore platform can overcome these limitations. Nanopore sequencing of total RNA yielded novel insights into the Pseudomonas aeruginosa transcriptome that—if replicated in other species—will change our view of the bacterial RNA world. The discovery of sense-antisense pairs of transfer-messenger RNA (tmRNA), tRNAs, and mRNAs indicates a further and unknown level of gene regulation in bacteria.

## INTRODUCTION

The gammaproteobacterium Pseudomonas aeruginosa is a globally distributed opportunistic pathogen that thrives in aquatic habitats and can colonize the animate surfaces of plants, animals, and humans (1). The P. aeruginosa population consists of three minor groups and two major groups, 1 and 2, that are designated by the mutually exclusive presence of the virulence effectors ExoS and ExoU (2). The 25 most frequent clones make up about 50% in the contemporary P. aeruginosa population (3, 4). The most common clone in strain collections is the ExoS-positive clone C (5–7), followed by the ExoU-positive clone PA14 (8–10). Complete genome sequences are available for the clone representatives C-NN2 (11) and UCBPP-PA14 (12). Clones C and PA14 are generalists that are widespread in environmental and disease habitats (4, 11), but their similar abundances do not correlate with a comparable body of knowledge of their lifestyle and physiology (7, 13). Whereas the UCBPP-PA14 strain has become a major workhorse to investigate virulence, biofilms, signaling, and quorum sensing (10, 14), most experimental work on ExoS-positive strains has been performed not on clone C but on a representative of a rare group 1 clone, i.e., strain PAO1, a burn wound isolate from the 1950s. PAO1 has been and still is the prototype to resolve the physiology, metabolism, and genome organization of P. aeruginosa (15–17).

When the first complete genome sequence of a P. aeruginosa strain was published (15), studies on the P. aeruginosa transcriptome became feasible. Gene expression profiling is instrumental to understand the lifestyle and pathogenicity of P. aeruginosa. Initially performed by microarray hybridization (18), high-throughput sequencing of cDNA fragments became a welcome alternative with the advent of next-generation sequencing platforms (19–21). Widespread adoption of this technology, given the popular name RNA-Seq (22) (or RNA-seq), led to an exponential increase in the number of whole-transcriptome experiments reported in the literature (21). An RNA-seq experiment will typically explore the consequence of an environmental or genetic perturbation on the levels of mRNA transcripts and small noncoding RNAs. However, short-read cDNA sequencing does not capture the complete RNA world. The depletion of rRNAs, the loss of strand specificity, and the fragmentation, size selection, and amplification during library preparation lead to inevitable losses of information about the initial composition of the RNA pool. Direct RNA sequencing could potentially avoid these limitations of current standard protocols of “cDNA-seq.”

The direct RNA sequencing technology developed by Oxford Nanopore Technologies (ONT) offers the possibility of sequencing native RNA molecules (23). Direct RNA-seq is strand specific, retains the native length and nucleotide composition of the RNA molecule, and avoids any bias introduced by reverse transcription and amplification. Despite these obvious advantages, the amount of direct RNA-seq data among published transcriptome studies is small, which could partly be attributed to the lack of convenient software pipelines for the processing and evaluation of the primary sequencing data (24). Within the microbial world, the main subjects of the few publications have been lower eukaryotes or RNA viruses (25–28). We identified only one bacteriological study that applied direct RNA-seq to determine the expression of the resistome in extensively drug-resistant Klebsiella pneumoniae isolates (29).

Here, we report on the direct RNA sequencing of the transcriptome of two members of P. aeruginosa clone C, i.e., the cystic fibrosis clinical isolate NN2 and the environmental aquatic isolate SG17M (30). RNA was isolated and processed from the growing bacteria as quickly as possible to gain an unbiased insight into the natural RNA repertoire of the bacteria. The bacterial transcriptomes obtained by direct RNA sequencing on the Nanopore platform revealed yet-undescribed RNA molecules and an unexpected composition of the pools of sense and antisense RNAs (asRNAs).

## RESULTS

### Genome sequence of P. aeruginosa clone C strains NN2 and SG17M.

P. aeruginosa strain NN2 is the first clone C isolate that was retrieved from a patient with cystic fibrosis at the onset of her more than 30-year-long airway colonization with clone C (31). The 6.90-Mbp large circular NN2 genome sequence is available as a single contig (European Nucleotide Archive [ENA] accession no. PRJEB2325) (11). P. aeruginosa strain SG17M is an environmental clone C isolate recovered from a river in Germany (5). A draft version of 17 scaffolds of the SG17M genome has been published (32). Since we envisaged to align the reads of direct RNA sequencing experiments to complete clone C genomes, the SG17M genome was resequenced by a combination of short-read (43-Mbp raw reads) and long-read (19-Mbp raw reads) sequencing. A single contig was *de novo* assembled from Nanopore DNA reads with a minimum read length of 8,000 bp and polished with paired-end Illumina reads, yielding a 6.88-Mbp large circular genome with a completeness score of 99.9% (33) (ENA accession no. PRJEB749924) (see Fig. S1A and B in the supplemental material). Of the close to 50 elements of the accessory genome of clone C that are absent in reference strain PAO1 (7), a gene cluster in strain SG17M, which encodes stress-related cargo gene products, has been experimentally characterized (34–36). Of these genes, we confirmed the map positions for *sHsp20c* (34) and the disaggregase genes *clpB*, *clpG_GI_*, and *clpG* (35). However, we also detected three further *ftsH* metalloprotease homologs in addition to the two already functionally characterized genes in the core and accessory genome (36) (Fig. S1C to E).

Strains NN2 and SG17M share their core genomes and most regions of plasticity (RGPs), with a sequence diversity of just 0.01% consisting of 109 intergenic single nucleotide variants (SNVs), 464 synonymous SNVs, and 158 nonsynonymous SNVs. Most sequence diversity among the 2,338 SNVs resides within PAGI-2 genomic islands (37). Strain NN2 harbors 181,188 bp of sequence in an integron, RGP5, RGP10, and PAGI-2 that is absent in strain SG17M. Conversely, SG17M includes 85 open reading frames (ORFs) that are absent in NN2 and are partially located in the strain-specific cargo region of genome island PAGI-3 (37). PAGI-2 and PAGI-3 are integrative and conjugative elements (ICEs) (38). They carry an individual cargo of genes and a conserved gene set that confers the integration, excision, and conjugal transfer of the island. These islands can be transferred to other strains and even across species barriers to other beta- and gammaproteobacteria (39).

### Direct RNA sequencing on the Nanopore platform.

RNA was extracted from P. aeruginosa NN2 or SG17M grown in lysogeny broth (LB) at 37°C until mid-exponential (4 h) or early stationary (8 h) phase. After poly(A) tailing and library preparation, RNA sequencing was performed with MinION flow cells in the 3′-to-5′ direction. Three biological replicates were investigated on separate occasions. Only 4 out of a total of 143,693 total spike-in enolase control reads (0.003%) were spuriously antisense associated, indicating that the strand specificity of the P. aeruginosa RNA molecules had been retained during processing and sequencing.

**FIG 1:**
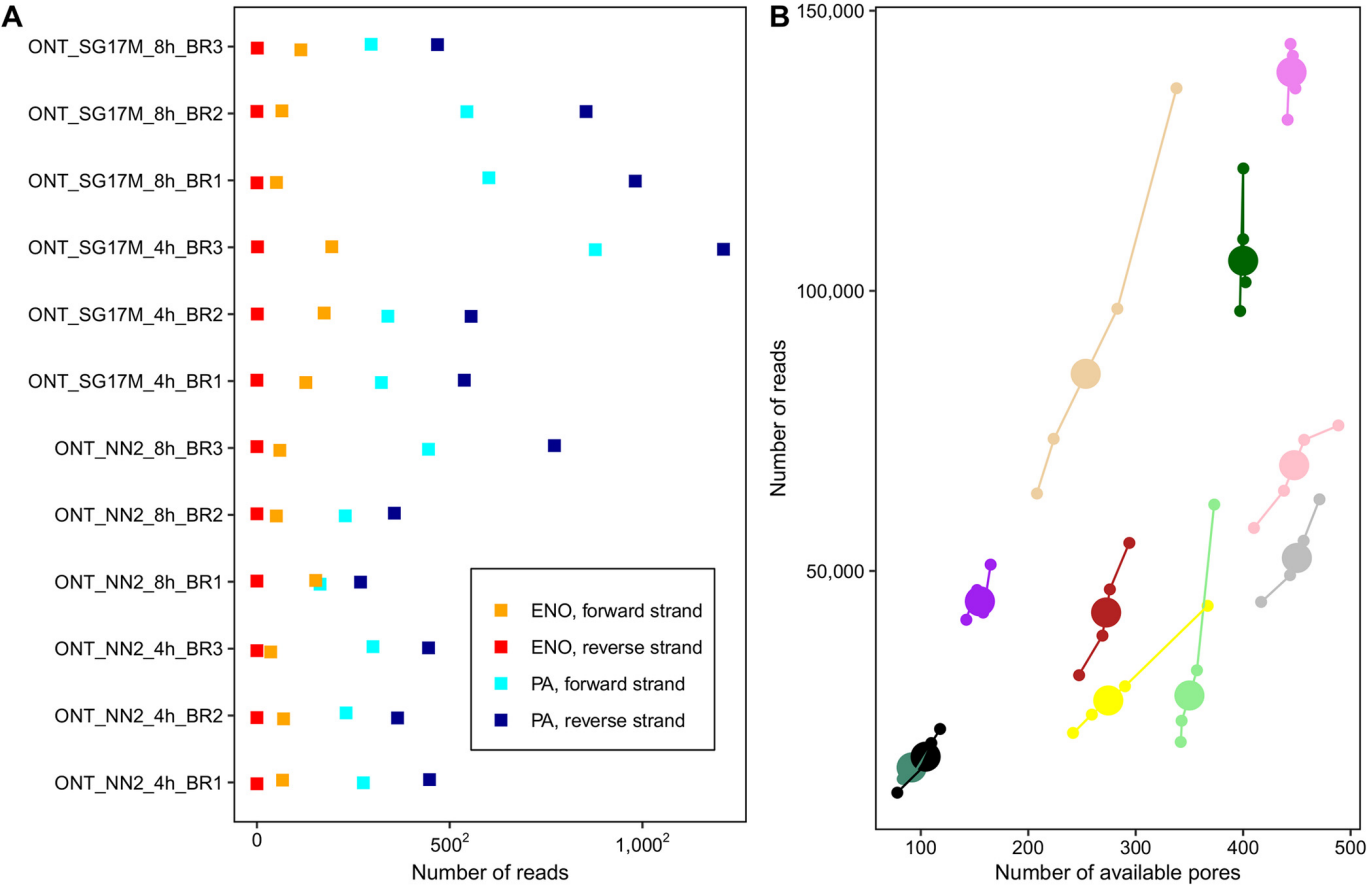
Evaluation of the Nanopore sequencing runs and genome-based read alignment with minimap2. (A) Representation of the number of P. aeruginosa-specific RNA reads aligning to either the forward (light blue) or the reverse (dark blue) strand of the corresponding reference sequence as well as the number of spike-in RNA control reads mapping to the forward (orange) or reverse (red) human enolase strand. Overall, 4 out of 143,693 total spike-in enolase reads (0.003%) were spuriously antisense associated. Results are shown for the three biological replicates (BR1 to BR3) of SG17M and NN2 at the mid-exponential phase (4 h) and early stationary growth phase (8 h). (B) During Oxford Nanopore sequencing runs, MUX sensor scans of pore fit were performed every 90 min to evaluate the number of available pores for the next 90-min sequencing period. Here, the number of reported available pores after MUX scan and the number of corresponding reads sequenced in the following 90-min period are visualized for the first 6 h. Each flow cell is represented by a unique color. The enlarged circle depicts the group centroid. A strong positive correlation between the number of available pores and the number of sequenced reads was detected (Pearson’s correlation coefficient = 0.8; Pearson’s *P* value < 0.0001; confidence intervals = 0.73 to 0.86).

The number of available pores and generated reads varied widely during sequencing runs and between the individual flow cells (Fig. 1). However, the distributions of read length for both sense and antisense RNAs ranging from about 100 to 5,000 nucleotides were remarkably similar in all 12 sequencing runs (Fig. S2 and S3), indicating that by the criterion of read length, similar repertoires of RNA molecules had been captured in all experiments. However, please note that our bead-based purification strategy which aimed at rescuing small RNA transcripts during sample processing shifted the read length to a factitious peak at 500 bp (Fig. S3).

### Repertoire of RNA molecules.

**FIG 2:**
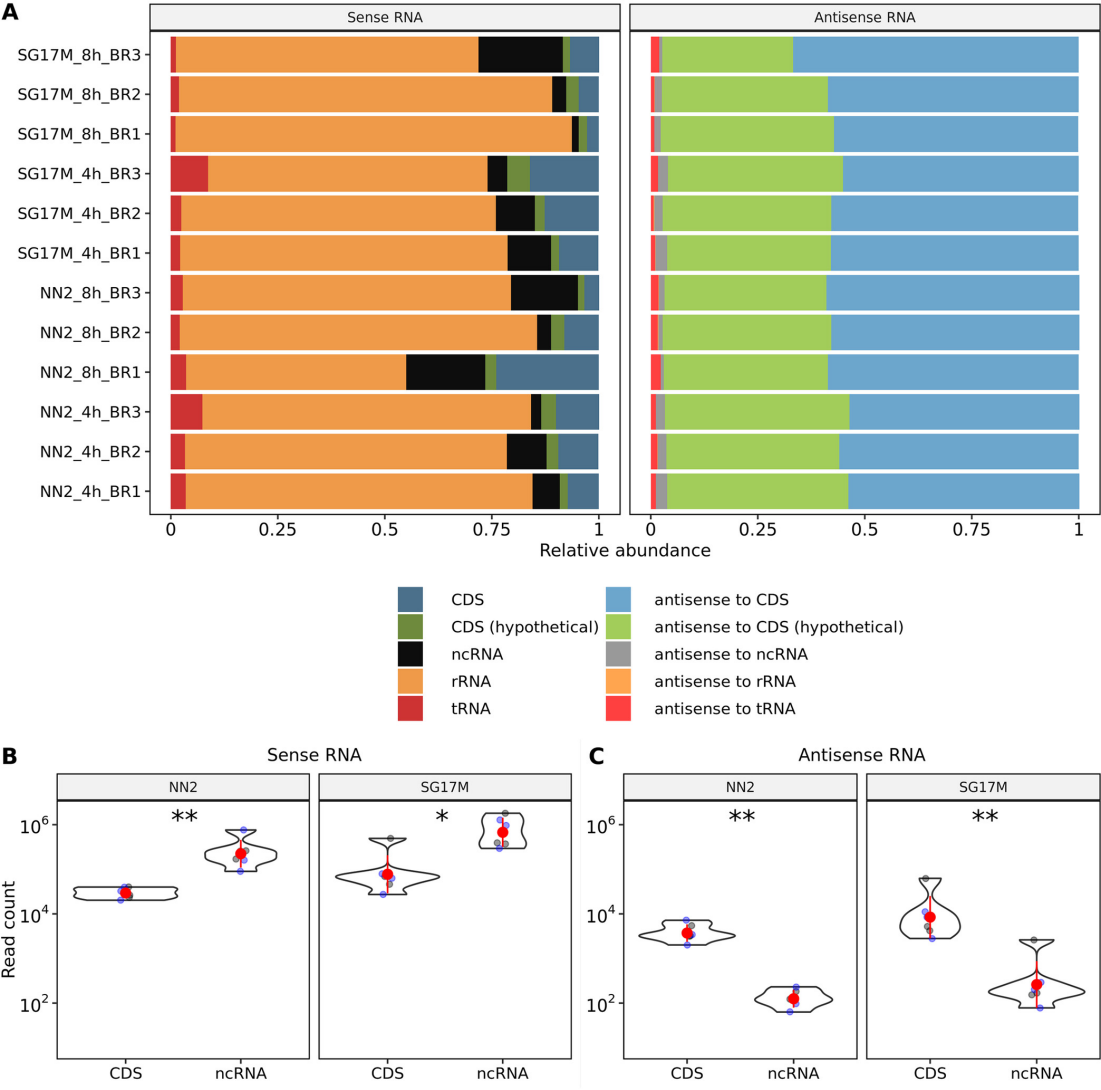
Abundances of sense and antisense transcripts with regard to the genomic feature types. (A) Relative abundances of sense (left) and antisense (right) RNA transcripts obtained from nonnormalized read counts in the biological replicates (BR1 to BR3) of SG17M and NN2 at 4- and 8-h time points. The reads were classified according to their alignment toward a known coding sequence (CDS), a coding sequence with no functional annotation (hypothetical), a noncoding sequence (ncRNA), rRNA (rRNA), and tRNA (tRNA). The variance observed between replicates and strains can be obtained from Fig. S4. (B) Representation of the log_10_-scaled counts of sense transcripts mapping to CDS or ncRNA regions in NN2 and SG17M at the mid-exponential phase (blue dots) and the early stationary phase (black dots). The red dots and red lines reveal the means and standard deviations. Most of the sense transcripts aligned to ncRNA regions in NN2 (Wilcoxon *P* value = 0.002; effect size *r* = 0.8; confidence interval = 0.63 to 0.85) and SG17M (Wilcoxon *P* value = 0.02; effect size *r* = 0.69; confidence interval = 0.26 to 0.85). (C) The log_10_-scaled antisense transcript count was significantly higher in CDS than in ncRNA regions with known and undefined annotations in both NN2 (Wilcoxon *P* value = 0.002, effect size *r* = 0.83; confidence interval = 0.63 to 0.85) and SG17M (Wilcoxon *P* value = 0.002; effect size *r* = 0.83; confidence interval = 0.63 to 0.85). *, *P* < 0.05; **, *P* < 0.01.

The RNA sequences were aligned to the NN2 and SG17M genomes. Fifty to ninety percent of the sense RNAs turned out to be rRNA molecules, followed by similar proportions of mRNA transcripts and noncoding RNAs (Fig. 2A, left). The pool of antisense RNA molecules mapped more than 90% to coding regions, with a slight preponderance for genes with functional annotation compared to hypotheticals of unknown function (Fig. 2B, right). Noncoding sense RNAs (including rRNAs) were 10-fold more abundant than sense coding sequences during the exponential and early stationary phases of growth in both NN2 and SG17M (*P = *0.002) (Fig. 2B). Antisense transcripts complementary to coding sequences dominated the antisense transcriptome more than 10-fold compared to antisense noncoding RNAs (*P = *0.002) (Fig. 2C). Principal-component analysis (PCA) of the alignment of the RNAs to the five transcript classes in Fig. 2A did not separate the individual experiments by strain or time point in the first dimension (Fig. S4). However, in the second dimension, the variance of sense and antisense RNAs was explained by the quantitatively small and very small contributions of noncoding RNAs, respectively. Thus, PCA told us that the global composition of the RNA repertoire varied to similar extents between biological replicates, growth phases, and strains.

### Comparison of direct RNA Nanopore sequencing with cDNA Illumina sequencing.

With the advent of second-generation sequencing technologies about 15 years ago, high-throughput short-read sequencing by synthesis on the Illumina platform has become the most common approach for the investigation of bacterial transcriptomes (21). Hence, to benchmark the novel strategy of direct RNA sequencing (ONT) with the established state of the art, we examined the P. aeruginosa NN2 transcriptome at mid-exponential and early stationary phases also by cDNA Illumina sequencing. The RNA preparations were split into two aliquots, one of which was treated with terminator 5′ phosphate-dependent exonuclease (TEX) (40, 41) to remove 5′ monophosphorylated RNA species. Thus, the Nanopore transcriptome (ONT) could be compared with the bulk cDNA transcriptome (0-TEX) and the cDNA transcriptome enriched for primary transcripts (TEX).

The paired comparisons (Fig. S5) revealed that sense and antisense transcript levels were most similar between ONT and TEX and most divergent between TEX and 0-TEX. Hence, our experiments detected more differences in gene expression between the primary and the bulk transcriptomes derived from the same RNA preparation than between biological replicates that had been processed by different sequencing technologies on separate days. Apparently, our Nanopore protocol primarily yielded a primary transcriptome.

### tRNAs.

Next, we compared the abundances of tRNAs in our samples because this class of RNA molecules has not received much attention in bacterial transcriptomics. A reason for the scarce literature may be the fact that both the Illumina and the ONT protocols do not detect aminoacylated tRNA molecules, because the 3′ end of the tRNA used for adapter ligation (Illumina) or polyadenylation (ONT) is blocked by the amino acid.

**FIG 3:**
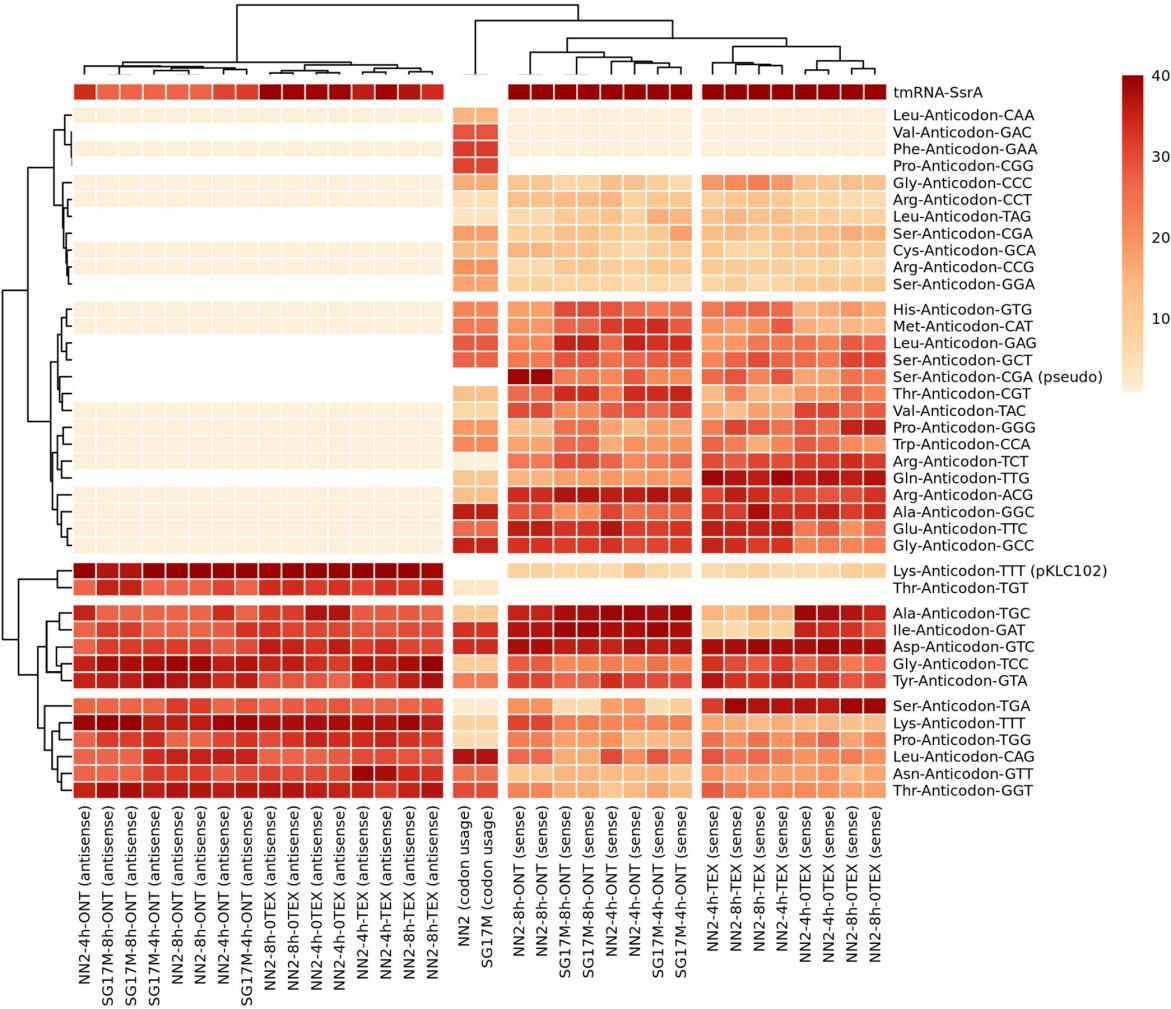
Rank-based scoring of sense and antisense tRNA abundance observed across biological replicates sequenced on the Illumina (TEX, 0-TEX) or Nanopore direct RNA-seq (ONT) platform versus codon usage in NN2 and SG17M. (Left) Ranking of antisense tRNA abundance. Thirteen high-ranking antisense tRNAs (red) and 16 low-ranking tRNAs (read count < 10) were detected (beige). The white color depicts the complete absence of sense and antisense transcripts. (Right) Ranking of sense tRNA abundance. Note that all 37 P. aeruginosa-specific anticodon alternatives without stop codons are included here and are summarized in Table S1. The ranking of tRNAs was performed by setting equal values (ties) to their minimum score. For constructing row and column dendrograms, complete-linkage clustering was performed based on a Euclidean distance matrix.

Like in other bacteria (42), the tRNA repertoires of the P. aeruginosa PAO1 (15) and clone C genomes (this work) consist of one, two, or three copies of 37 different isoacceptor genes plus three stop codons (Table S1). The ONT and Illumina platforms detected similar transcript levels of uncharged tRNA isoacceptors in the clone C biological replicates (Fig. 3). Moreover, both ONT and Illumina workflows discovered 13 antisense tRNAs, with a minimum of 10 reads across all samples (Fig. 3 and Table S2). Antisense tRNAs are common in eukaryotes, particularly among mitochondrial tRNAs, but to our knowledge have so far been described for just one archaeon species in prokaryotes (43). Eleven of the 13 antisense tRNAs had highly expressed sense counterparts (Fig. 3 and Table S2). When we predicted sense and antisense secondary structures of the 13 sense-antisense pairs with the RnaFold 2.4.18 software of the ViennaRNA package 2.0 (44) based on free energy minimization algorithms (44, 45), we found generally no significant difference in the thermodynamic stability between antisense and sense tRNA structures (Fig. S6). As reported for mitochondrial data sets (46), the sense tRNA anticodon predicted the antisense tRNA anticodon (anticodon symmetry). The only exception to this rule was the Thr-anticodon-TGT tRNA pair with high antisense and low sense tRNA ranking scores. For all other tRNAs, no antisense tRNA or only trace amounts (<10 reads in 16 entities) were detected (Fig. 3 and Table S2). In these cases, the thermodynamic stability of predicted secondary structures of sense tRNAs was higher than for corresponding antisense tRNA structures, suggesting that the formation of antisense tRNA was energetically not favorable (Fig. S6 and Table S2).

The most abundant molecule in the tRNA pool was the transfer-messenger RNA (tmRNA) SsrA, known to play a key role in the rescue of ribosomes, stalled on a truncated mRNA and the tagging of the nascent peptides for degradation (47, 48). Consistent with the literature, SsrA was more abundant in stationary phase than during exponential growth, but otherwise, the composition of the pool of uncharged tRNAs did not differ significantly by strain or growth phase (Fig. 3). Interestingly, the complementary antisense tmRNA was also abundant (Fig. 3). The second most common molecules in this pool were two antisense tRNA^Lys^. The version with a longer tail at the 3′ end (−) Lys-anticodon-TTT-pKLC102 (Fig. S7) extends into the plasmid attachment site of the genomic island pKLC102, which integrates into the 3′ end of the chromosomal tRNA^Lys^ gene close to the *pilA* locus (49). pKLC102 is a highly mobile genomic island, with spontaneous chromosomal excision rates in the steady state of at least 10^−1^ (39).

In Fig. 3, the abundance of uncharged tRNA transcripts is clustered by Euclidian distance with the codon usage in P. aeruginosa SG17M and NN2 clone C isolates. According to Spearman’s correlation analysis (Fig. S8), the overall abundance of uncharged sense tRNA transcripts significantly correlated with genomic codon usage, with average correlation coefficients of about 0.3 and 0.2 for experiments based on the Nanopore and Illumina platforms, respectively. However, the inspection of the individual associations uncovered a more complex picture. For example, the rarest arginine codon was paired with the most abundant tRNA^Arg^, and the tRNA^Arg^ gene is missing (15) for the most frequent arginine codon (Fig. 3).

### Antisense RNAs.

**FIG 4:**
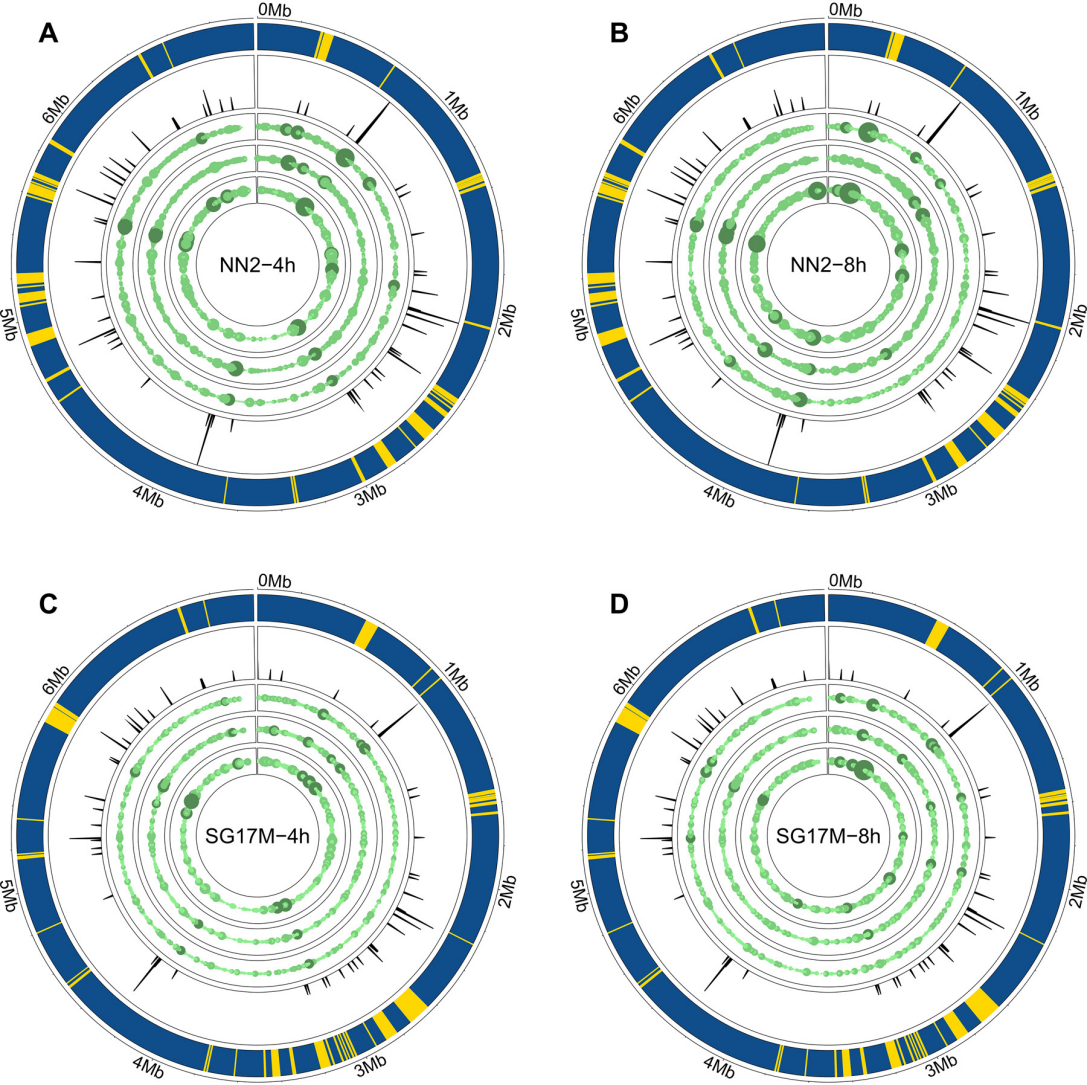
Genome visualization of antisense transcript hot spots in P. aeruginosa clone C isolates with the R package circlize (67). The outermost circle represents the core (blue) and accessory (yellow) genome as predicted by IslandViewer4 (68). The second circle shows the frequency of tRNAs in the genome sequence. The third, fourth, and fifth lanes depict antisense coverage in the first, second, and third biological replicates of NN2 in mid-exponential phase (A), NN2 in early stationary phase (B), SG17M in mid-exponential phase (C), and SG17M in early stationary phase (D). The number of antisense transcripts was normalized beforehand (TMM-normalized log_2_-scaled CPM). The higher the antisense transcript coverage, the larger the circle and the darker the green color. The overall design of the figure was inspired by Fig. 6 in the publication of Wurtzel and colleagues (20) to allow direct comparison between their and our data.

More than 90% of the antisense RNA molecules were complementary to a coding sequence. Only a few molecules were antisense tRNAs (see above) or matched with a complementary noncoding sequence other than rRNAs (Fig. 2). The Genome Atlases (20) visualize the quantitative distribution of antisense RNAs on the NN2 and SG17M chromosomes (Fig. 4). In all experiments, the antisense RNAs were evenly distributed in the genomes. Direct RNA sequencing identified medians of 4,100 (median absolute deviation = 1,045) and 4,695 (median absolute deviation = 2,441) distinct nonoverlapping antisense RNAs during exponential and stationary growth, respectively (Table 1). Although the numbers of antisense RNA entities were not significantly different between the experiments, we noted increased accumulation of antisense reads in distinct hot spot regions in biological replicates of NN2 compared to SG17M (Fig. 4 and Fig. S9A and B). Hot spots of the 3% highest expression in NN2 and SG17M are shown in Table S3.

**TABLE 1:** Overview of sequencing and alignment parameters obtained for the three biological replicates per strain and time point

Sample	Isolate	Time point (h)	Replicate	Flow cell identifier	Duration of base calling in ms (days)^*a*^	No. of sequenced bases	No. of antisense transcripts with no overlap	Error rate^*b*^
NN2_4h_BR1	NN2	4	1	FAO52629	286,670,962 (3.3)	91,645,543	2,653	0.11
NN2_4h_BR2	NN2	4	2	FAM92370	234,696,844 (2.7)	67,762,786	2,712	0.11
NN2_4h_BR3	NN2	4	3	FAL84182	326,128,171 (3.8)	103,114,020	4,803	0.21
NN2_8h_BR1	NN2	8	1	FAO52848	223,471,116 (2.6)	65,434,441	1,722	0.09
NN2_8h_BR2	NN2	8	2	FAM95403	107,231,447 (1.2)	31,702,988	3,045	0.11
NN2_8h_BR3	NN2	8	3	FAO52661	555,460,919 (6.4)	260,692,826	6,345	0.11
SG17M_4h_BR1	SG17M	4	1	FAO52663	407,266,972 (5.7)	150,006,926	3,612	0.11
SG17M_4h_BR2	SG17M	4	2	FAL55242	362,454,058 (4.2)	160,857,070	4,587	0.10
SG17M_4h_BR3	SG17M	4	3	FAM95414	956,237,877 (11.1)	547,180,145	56,583	0.12
SG17M_8h_BR1	SG17M	8	1	FAO52611	438,286,716 (5.1)	252,533,658	7,395	0.11
SG17M_8h_BR2	SG17M	8	2	NA^*c*^	476,546,009 (5.5)	225,927,528	9,490	0.11
SG17M_8h_BR3	SG17M	8	3	FAO52535	368,466,527 (4.3)	98,126,611	2,512	0.11

a All computation was performed on the internal MHH HPC-seq 1000 core Ubuntu Linux compute cluster using software described at https://github.com/colindaven/guppy_on_slurm.

b Error rate was obtained with samtools stats and displays the ratio between mismatches and bases mapped. The “error rate” reflects the high rate of posttranscriptional base modifications of natural RNA. During base-calling the raw current signals of the Nanopore are transformed into the typical four-letter genetic code (A, G, U, and C) and information on base modification is no longer stored in the translated sequences. This loss of information turns up in the system as high error rate.

c NA, not available.

**FIG 5:**
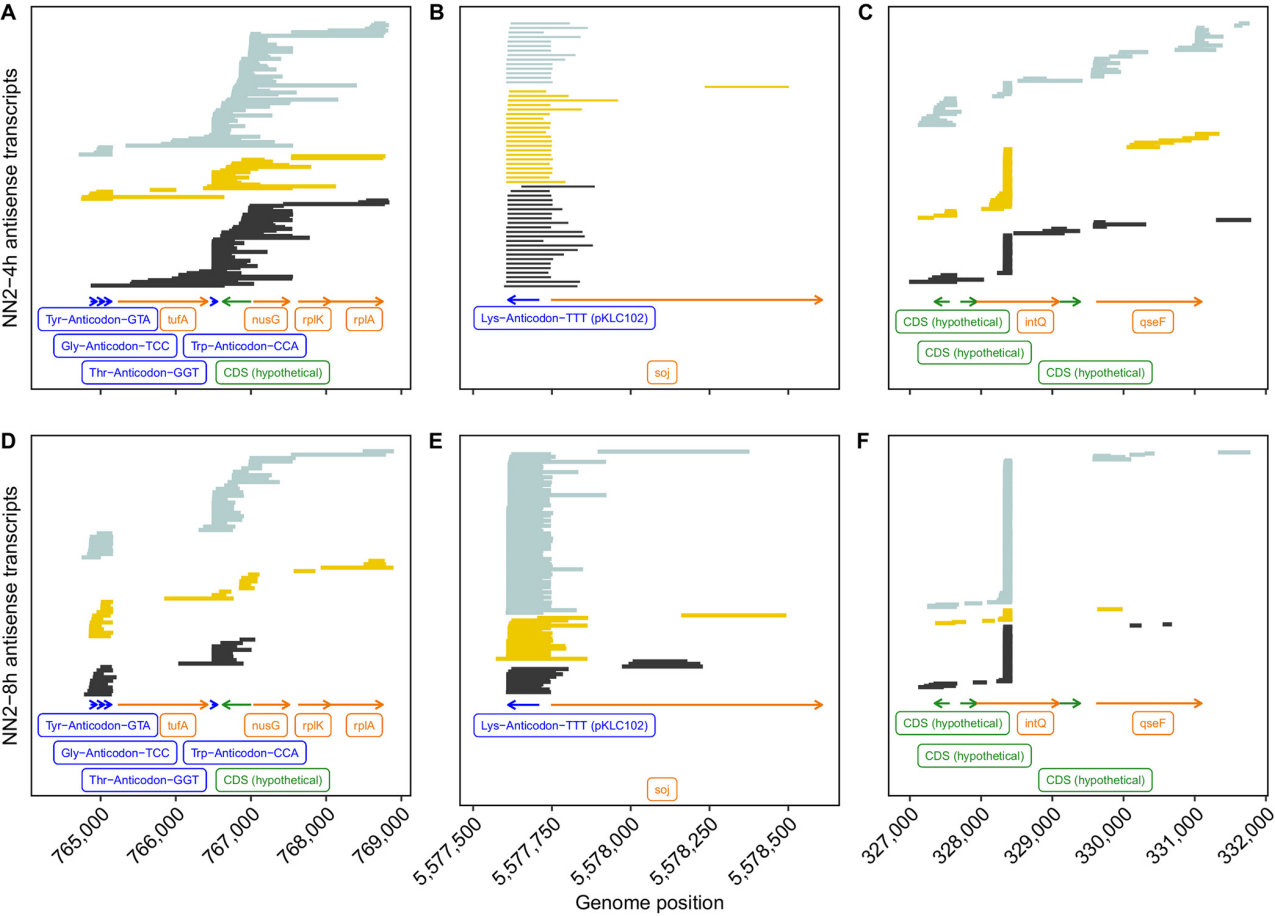
Three antisense RNA coverage hot spots in P. aeruginosa NN2 during mid-exponential (upper) and early stationary (lower) growth. Antisense RNA reads of the biological replicates 1 (black), 2 (yellow), and 3 (light blue) were aligned to the genome sequence with minimap2. Green and orange arrows represent read alignments toward coding sequences with no functional annotation (CDS, undefined) and known coding sequences, respectively. The dark blue label depicts tRNA coverage. The abscissa provides the map position of the selected genomic region. Please note that the bead-based purification of RNA prior to sequencing introduced a skew toward shorter and more heterogeneous read lengths.

**FIG 6:**
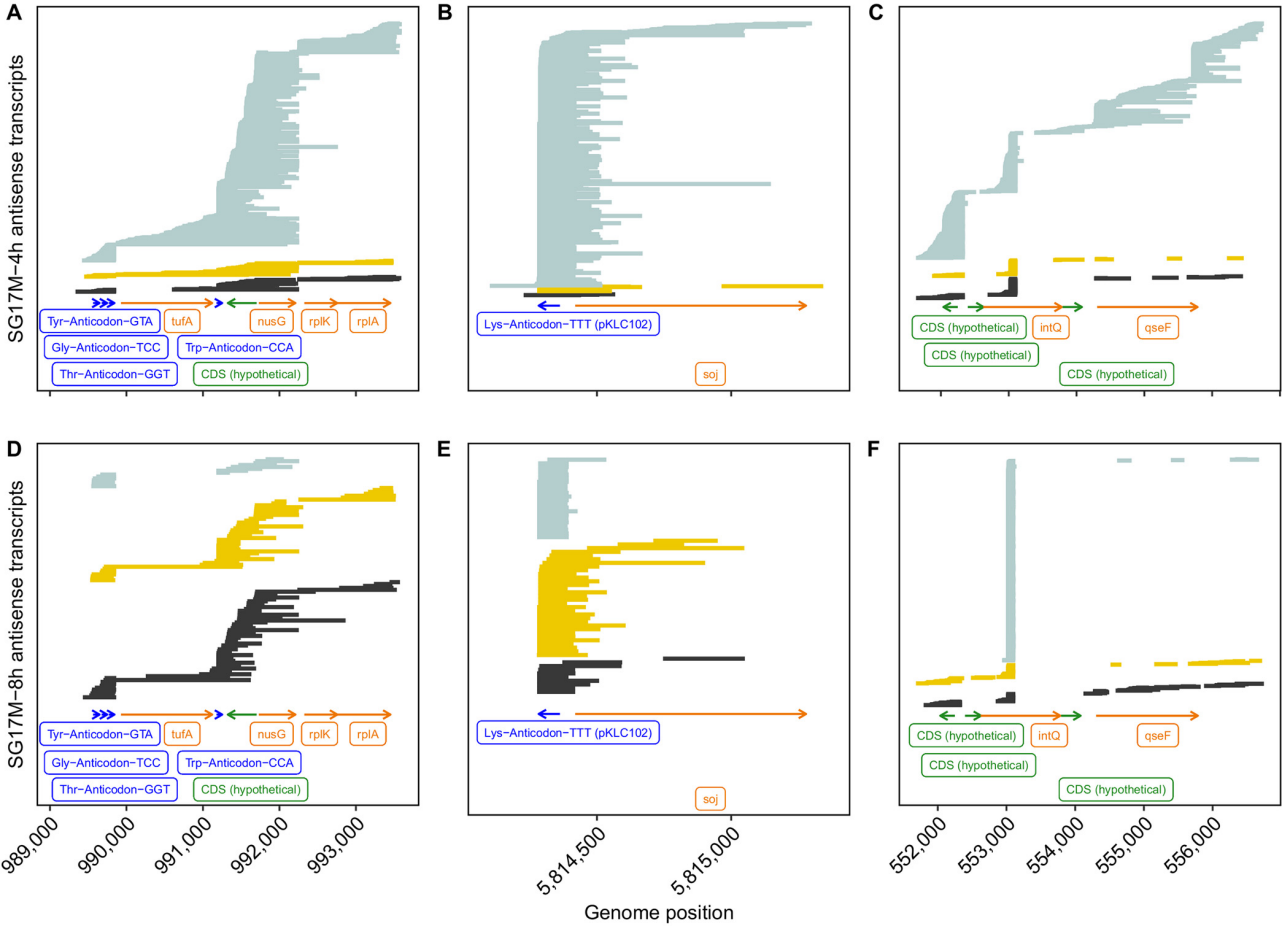
Three antisense RNA coverage hot spots in P. aeruginosa SG17M during mid-exponential (upper) and early stationary (lower) growth. Antisense RNA reads of biological replicates 1 (black), 2 (yellow), and 3 (light blue) were aligned to the genome sequence with minimap2. Green and orange arrows represent read alignments toward coding sequences with no functional annotation (CDS, undefined) and known coding sequences, respectively. The dark blue label depicts tRNA coverage. The abscissa provides the map position of the selected genomic region. Please note that the bead-based purification of RNA prior to sequencing introduced a skew toward shorter and more heterogeneous read lengths.

To provide an impression about the spectrum of antisense RNA molecules in the clone C strains, we selected three genomic regions to which a comparably large number of antisense RNA reads had been mapped in all experiments (Fig. 5 and 6). Panels A and D of Fig. 5 and 6 show the reads of strains NN2 (Fig. 5) and SG17M (Fig. 6) that were aligned from the 4-h (A) and 8-h (D) biological replicates to a gene cluster evolutionarily highly conserved in gammaproteobacteria (50). The elongation factor Tu *tufB* gene is flanked upstream by the triplet tRNA^Tyr^-tRNA^Gly^-tRNA^Thr^ genes and downstream by the tRNA^Trp^ gene, followed by the genes *secE*, *nusG*, *rplK*, and *rplA*, encoding a protein cotranslocator, a transcription termination factor, and two ribosomal proteins. The majority of antisense RNA reads either covered the triple tRNA gene cluster or aligned to the gene contig tRNA^Trp^ gene-*secE-nusG* and parts thereof. A minority of >1-kb-long molecules mapped also to the other genes. This example shows a preponderance of conserved transcription and termination sites but also a large population of molecules with variable lengths and map positions. The next two examples demonstrate that this variability is not a technical artifact introduced during sample processing but reflects the natural variation of antisense RNA in P. aeruginosa. The second example (Fig. 5B and E and Fig. 6B and E) deals with the genomic integration site of the mobile genomic island pKLC102 (39, 49). Almost all antisense RNA molecules retrieved from this region start at the 5′ end of the tRNA^Lys^ gene and span the *att* integration site of pKLC102, and a few of them extend beyond into the chromosome partitioning gene *soj*. The third example (Fig. 5C and F and Fig. 6C and F) was taken from the region of the accessory genome closest to the origin of replication. Here, the population of antisense RNA molecules is dominated by a small RNA that is complementary to the intragenic sequence at nucleotide positions 373 to 507 of the 1,161-bp phage-related site-specific integrase *intQ* gene (Fig. S9C and D). A few antisense RNA molecules mapped to other sequences of this region.

### Strain- and growth phase-specific expression signatures.

**FIG 7:**
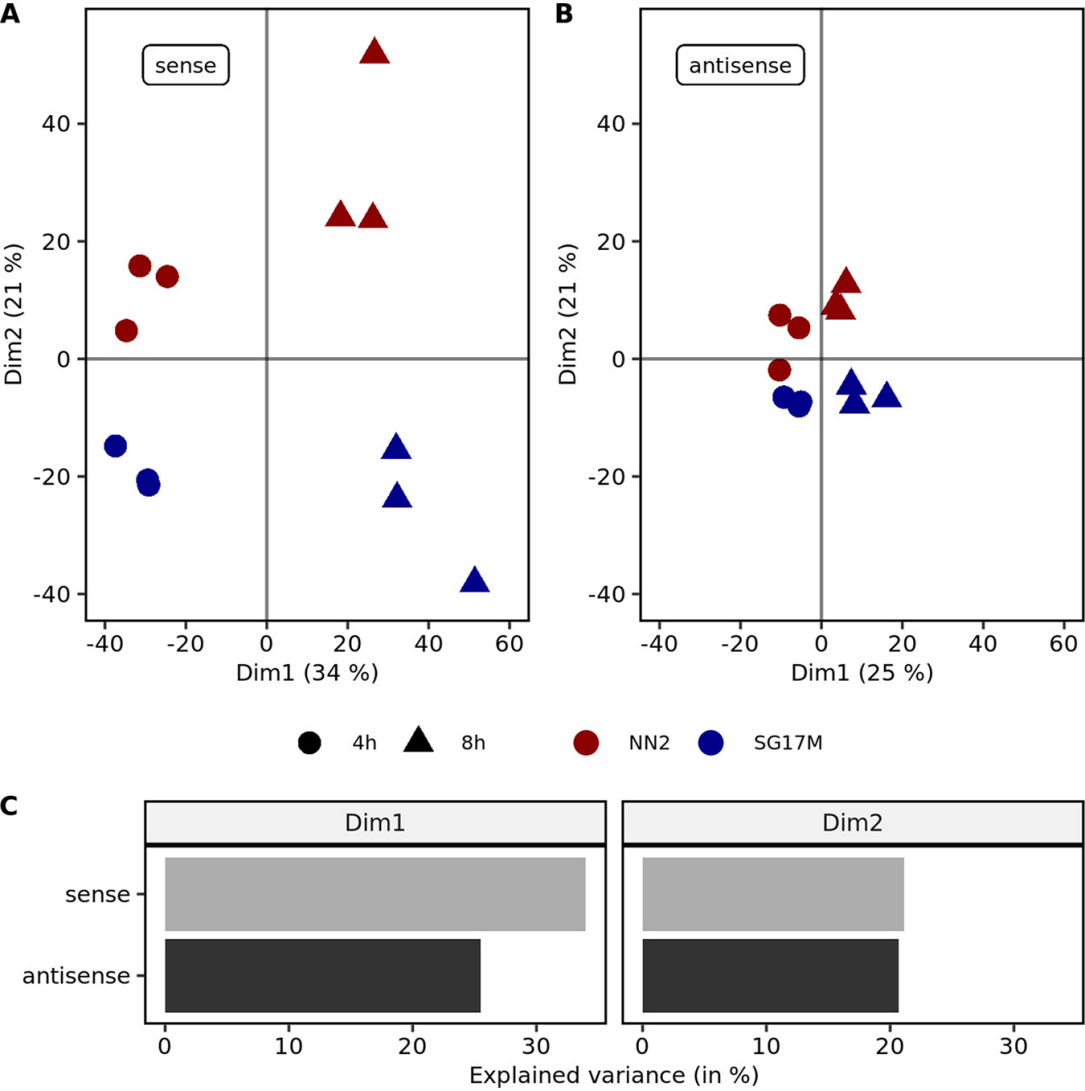
Principal-component analysis (PCA) of sense and antisense mRNA expression. (A) The first principal component in sense mRNA expression PCA explains 34% of the observed variance mainly between the mid-exponential (circles) and early stationary (triangles) growth phases. The second principal component explains about 21% of the variance and predominantly separates NN2 (red) and SG17M (blue) biological replicates. (B) The first principal component in antisense mRNA expression PCA explains 25% of the observed variance mainly between the mid-exponential (circles) and early stationary (triangles) growth phases. The second principal component explains about 21% of the variance and predominantly separates NN2 (red) and SG17M (blue) biological replicates. (C) In the first dimension (Dim1), which was found to explain the variance between time points, sense mRNA expression is slightly more important. For the second dimension (Dim2), which separates the SG17M and NN2 isolates, antisense and sense mRNA expressions contribute equally to the variance.

Next, we wanted to explore whether the pools of mRNA and antisense RNA molecules were carrying diagnostic signatures that differentiate mid-exponential and stationary growth in NN2 and SG17M from each other. PCA indeed segregated the four experimental groups by growth phase in the first dimension and by strain in the second dimension whereby the biological replicates clustered together (Fig. 7). Gene expression profiles were more distinct between the sense mRNA but rather similar between the antisense RNA populations.

**FIG 8:**
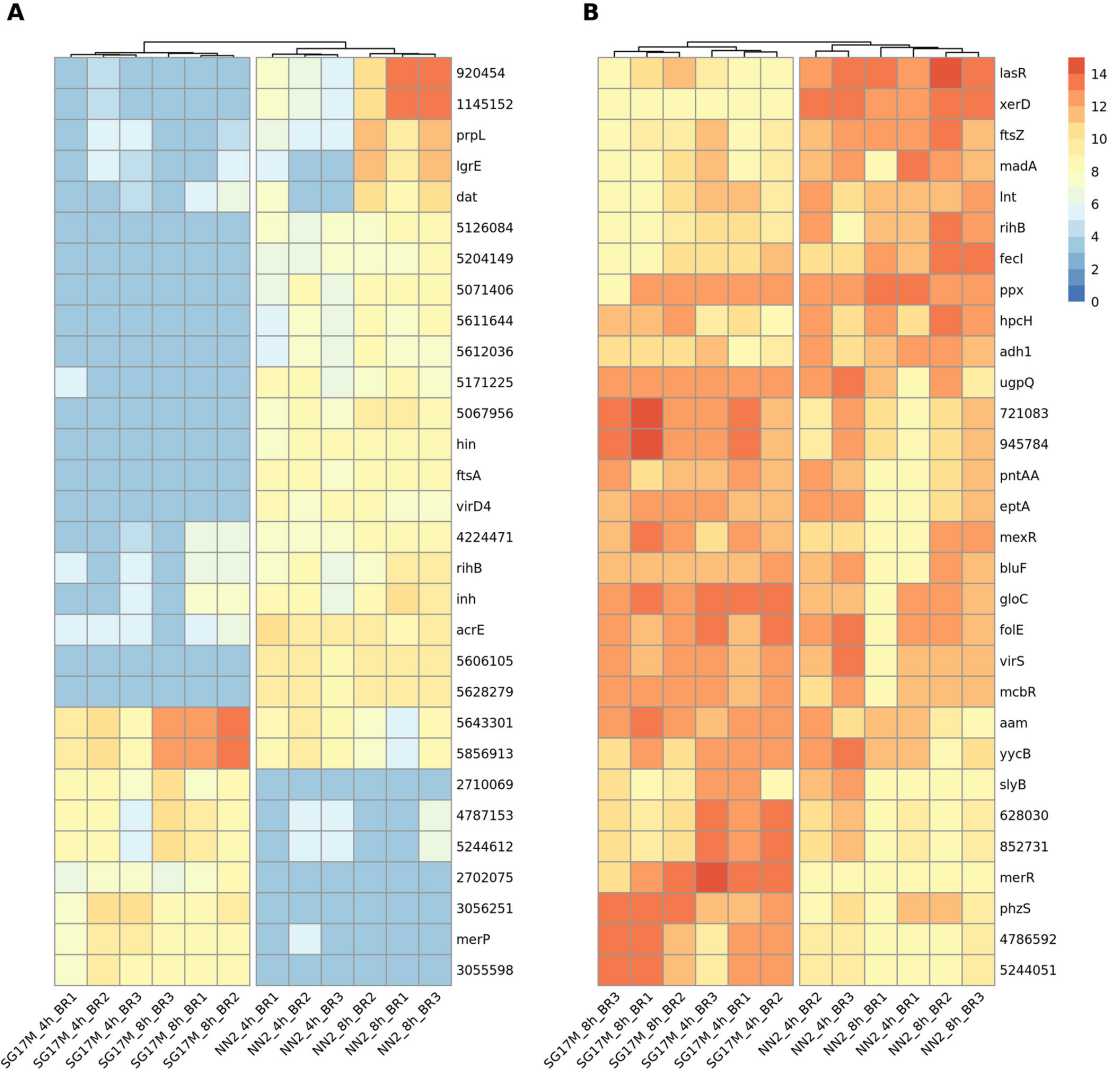
Heat map representation of the 30 variables contributing most to the replicate separation in the second dimension of the principal-component analysis. The second dimension was found to explain differences between P. aeruginosa clone C strains NN2 and SG17M. (A) The 30 most differentially expressed sense coding sequences between NN2 and SG17M. For coding sequences without functional annotation, the genome position is provided. (B) The 30 most differentially expressed antisense coding sequences between NN2 and SG17M. For coding sequences without functional annotation, the genome position is provided. For the column-based dendrogram analysis, a complete clustering method based on Euclidean distances of normalized read counts (TMM-normalized log_2_-scaled CPM) was chosen.

**FIG 9:**
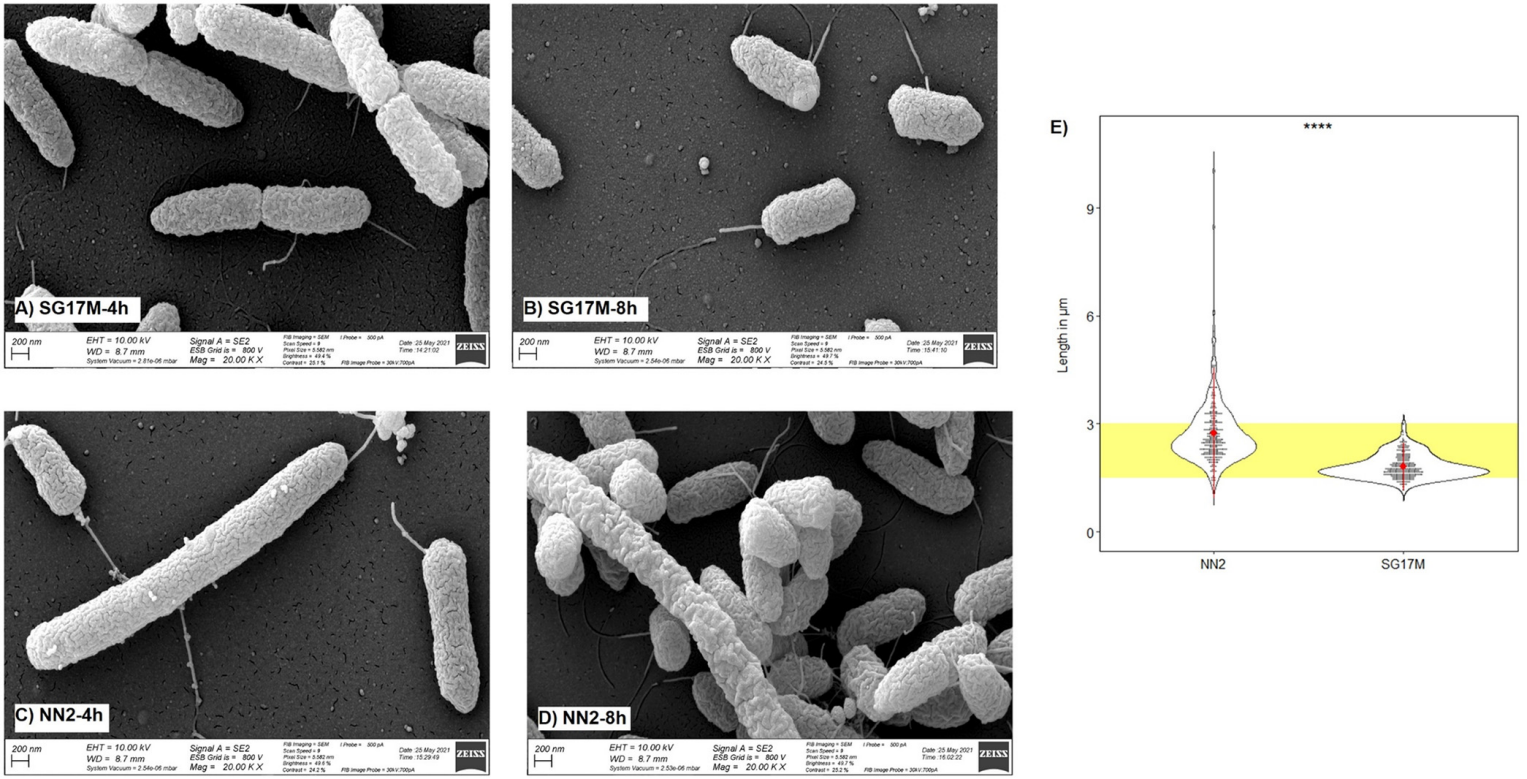
Electron microscopy of P. aeruginosa clone C bacteria. (A) SG17M, mid-exponential phase; (B) SG17M, early stationary phase; (C) NN2, mid-exponential phase; (D) NN2, early stationary phase. (E) Distribution of bacterial cell length of two biological replicates per strain and time point. ****, *P < *0.0001.

Figure 8 provides an overview of the top 30 sense and antisense transcripts that are most differentially expressed between the clinical isolate NN2 and the environmental isolate SG17M during planktonic growth in LB. Most antisense transcripts mapped to a locus of the core genome that encodes an annotated function on the opposite strand. Conversely, the majority of mRNAs had been transcribed from a gene in the accessory genome and/or from an open reading frame that lacks functional annotation. Thus, we concluded that the impact of the differential transcriptome on bacterial phenotype could not be predicted *prima facie* from the sequence data. The only clear exceptions were the cell division genes *ftsA* and *ftsZ*. And indeed, the NN2 bacteria were longer and showed a broader size distribution than the SG17M bacteria (Fig. 9 and Fig. S10), implying that the differential expression profile of FtsA mRNA and antisense FtsZ RNA was translated into different shapes of the cells.

## DISCUSSION

Direct RNA sequencing of transcriptomes of a clinical and an environmental isolate of P. aeruginosa clone C provided a representative overview of the various transcript categories. We consider the simultaneous sequencing of coding and noncoding RNAs in both sense and antisense directions to be a major step forward in the understanding of the bacterial transcriptome compared to the common focus on the repertoire of reverse-transcribed mRNA transcripts.

An unexpected finding was the detection of antisense tRNA molecules. Sense-antisense pairs of tRNAs are common among mitochondrial tRNAs (51) but to our knowledge have only reported once in the bacterial world, for the archaeon Sulfolobus solfataricus (43). Our *in silico* simulations suggest that like in mitochondria (46), the matching thermodynamic stability is key that both the sense and the antisense versions of the tRNA coexist in the cell. It will be interesting to learn in the future whether antisense tRNAs are widespread in the bacterial world. We assume that imperfections of the routine bioinformatics pipelines have impeded the detection of antisense tRNAs in transcriptome data sets.

A particular case was tRNA^Lys^. The sense tRNA^Lys^ was lowly expressed, but the antisense tRNA^Lys^ was the most abundant antisense tRNA in the bacterial cell. We assume that this scenario is probably specific for clone C and does not apply to all P. aeruginosa strains. Downstream of the two tRNA^Lys^ loci, the clone C genome includes the irreversibly incorporated genomic island PAGI-4 close to *oprL-phnAB* and pKLC102, the most mobile ICE known to date, close to *pilA* (49). Coverage of tRNA coding sequence and the downstream *att* integration site by the antisense tRNA^Lys^ could downregulate the frequency of reversible excision and integration of chromosomal pKLC102. Planktonic SG17M bacteria carry about 30 circular pKLC102 molecules per host chromosome, and the proportion of pKLC102-free chromosomes increases from 2% to 3% in mid-exponential phase to about 10% in stationary phase (39). The antisense tRNA^Lys^ could be an important control element to keep pKLC102 in the host chromosome.

**FIG 10:**
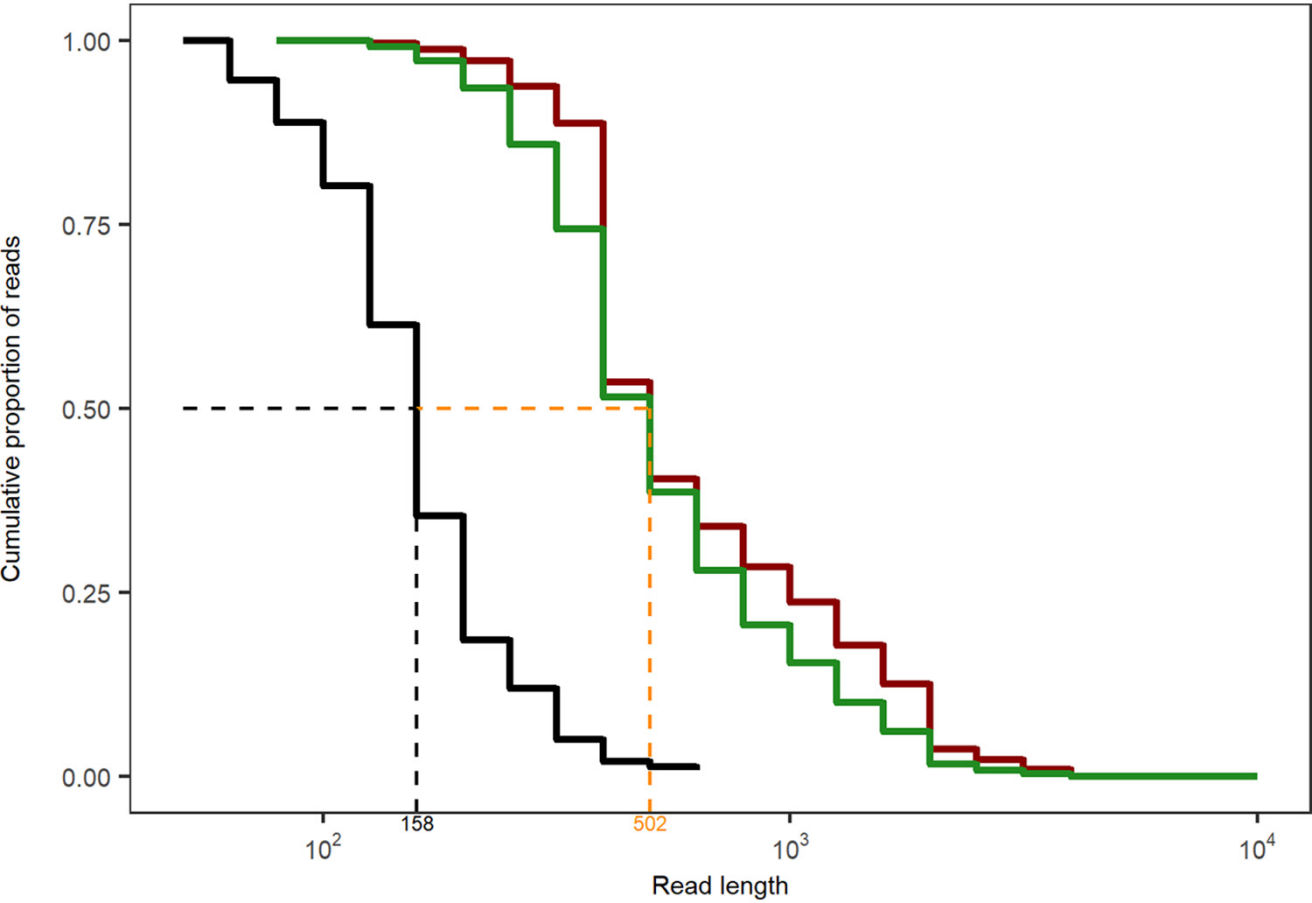
Kaplan-Meier plot of the antisense RNA length distribution obtained with short-read cDNA sequencing on the Illumina platform (53) (black line) or by direct full-length RNA Nanopore sequencing (this work) of NN2 (red line) and SG17M (green line). The median length of antisense transcripts observed by Nanopore sequencing (orange) is similar to the maximum read length observed with Illumina.

Besides the antisense tRNAs, almost all antisense RNA (asRNA) molecules mapped to coding sequences consistent with the three previous reports that dealt with antisense RNAs in P. aeruginosa (20, 52, 53). The first study by Wurtzel and colleagues (20) identified 384 distinct asRNAs in strain PA14, which is about 1 order of magnitude less than recorded by direct RNA-seq in the clone C strains. Ferrara and colleagues (52) noted the strain specificity of expression for the majority of their 60 detected asRNAs. In the clone C strains, we observed such variable expression not only between strains but also between growth phase and biological replicates, particularly if the asRNA entity was present in low copy numbers (see the examples in Fig. 5 and 6). The most extensive study of cDNA sequencing on an Illumina platform identified 232 different asRNAs of 50 to 581 nucleotides in length (53). The majority of asRNAs were small: only 10% were 200 to 300 bases, and 3% were longer than 300 bp. Most asRNAs overlapped one gene transcribed from the opposite strand; only 13 asRNAs (6%) overlapped two contiguous genes. Our direct RNA sequencing unraveled a different composition of the asRNA repertoire in the P. aeruginosa transcriptome. Besides some highly expressed small asRNAs (less than 200 bases), a population of longer asRNAs was sequenced that could be complementary to a contig of several genes and covered a broad range (a few hundred to thousands of bases) (Fig. 10). The Kaplan-Meier plot in Fig. 10 illustrates the different length distributions of asRNA molecules of planktonic P. aeruginosa retrieved by short-read cDNA sequencing on the Illumina platform (51) and by direct full-length RNA Nanopore sequencing (this work). The Nanopore RNA-seq data show a length distribution of the population of asRNAs similar to that of coding mRNA transcripts. The median length of antisense transcripts observed by Nanopore sequencing is similar to the maximum read length observed with Illumina (Fig. 10). Since the discovery of large asRNA molecules changes our view about the makeup and role of asRNAs, replication studies with other taxa should clarify whether long gene-spanning asRNAs are common in bacteria.

Direct RNA sequencing was rewarding in the detection of RNA entities that are typically not covered by conventional cDNA-seq protocols. However, we observed several shortcomings of the current Nanopore RNA pipeline. First, base-calling was time-consuming and computationally intensive on central processing unit (CPU) hardware, though this was completely alleviated later after acquisition of graphics processing unit (GPU) hardware. Second, flow cells exhibited variable quality. Prior to starting the sequencing experiments, quality checks of flow cells were performed according to the protocol. Oxford Nanopore Technologies offers to replace any flow cells with less than 800 pores available for sequencing. However, our work plan required immediate sequencing of the processed samples, and flow cells should not be stored for more than 3 months. We hence initiated sequencing on flow cells with less than 800 pores. Consequently, some experiments were executed with poorly performing flow cells with a low number of available pores (Fig. 1). Hopefully this limitation will be overcome in the future by a more reproducible production of high-quality flow cells and the development of direct RNA multiplexing kits by Oxford Nanopore Technologies to examine biological replicates within the same cell. Moreover, if the polyadenylation of the 3′ end could be skipped, RNAs with blocked 3′ ends, like aminoacylated tRNAs, could be sequenced. The downsizing of the demands of the current protocol of several micrograms of RNA as starting material would allow the analysis of minute and/or scarce sources that are typical for most real-world applications from medicine and the life sciences. If in parallel the databases were fed with more primary electric current signals of matching sequences with and without base modifications, improved software and algorithms would pave the way to decode the epitranscriptional landscape of native RNAs (54).

Then direct RNA sequencing could become an integral component of the portfolio for bacterial transcriptomics and may even replace conventional cDNA-seq in many applications. We can envisage that direct RNA-seq could deliver unbiased, quantitative, and comprehensive strand-specific transcriptomes including meaningful information about RNA isoforms, antisense RNAs, operon structure, and transcription initiation and termination sites.

## MATERIALS AND METHODS

### Strains.

The P. aeruginosa clone C isolates NN2 and SG17M were stored in triplicate as glycerol stock cultures at −80°C. To assess the cell morphology of the strains, the bacteria were grown at 37°C in LB until mid-exponential and early stationary phases, precipitated, resuspended to a density of 10^7^ bacteria/mL, and then immediately examined by light and electron microscopy at the Core Unit for Electron Microscopy (Jan Hegermann).

### Bacterial culturing.

Bacteria were cultured in 25 mL of LB overnight at 37°C on a shaker platform (150 rpm). Two uninoculated LB controls were processed in parallel. After 18 h, fresh LB suspensions (five technical replicates per strain) were set up with starting optical densities at 600 nm (OD_600_) of 0.05 and two uninoculated LB controls. The newly prepared bacterial suspensions were grown at 37°C on a shaker platform (150 rpm). After 4 h (mid-exponential phase) and 8 h (early stationary phase), OD_600_ values of all replicates were obtained. At both time points, a subsample of each bacterial suspension (0.5 mL) was separately mixed with 1 mL of TRIzol reagent (Invitrogen; 15596026) for RNA extraction and subsequent Nanopore sequencing.

### RNA extraction and subsequent cDNA sequencing on the Illumina platform.

RNAprotect (Qiagen, Hilden, Germany) was added to each aliquot after sampling and centrifuged for 10 min at 5,000 × *g*. The pellet was frozen at −80°C until use. For RNA isolation, the bacterial pellet was resuspended in 400 μL of Tris-EDTA (TE) buffer containing 1.5 mg/mL of lysozyme, and cells were lysed for 15 min at room temperature. Thereafter, RNA was prepared with the RNeasy minikit (Qiagen, Hilden, Germany) following the protocol of the manufacturer. The RNA preparation was purified from contaminating DNA by two cycles of on-column DNase I digestion. RNA was eluted from the column with 2 × 75 μL of H_2_O. After addition of 2 μL of RNasin, the solution was stored at −80°C. The quality of the RNA preparation was checked with an Agilent 2100 BioAnalyzer (Santa Clara, CA).

Pairs of strand-specific cDNA libraries were prepared by the Eurofins Genomics Europe Sequencing Laboratory in Constance, Germany. One 0-TEX library was generated from bulk RNA for global transcriptome analysis, and the other library of enriched primary transcripts was generated from RNA that had been preincubated with terminator 5′ phosphate-dependent exonuclease (TEX) to remove 5′ monophosphorylated mRNA species (40, 41). The cDNA libraries were sequenced on an Illumina HiSeq 2000 instrument.

### RNA extraction for Nanopore sequencing.

The TRIzol suspension was vigorously shaken and incubated for 5 min at room temperature (RT) with regular tube inversions. Chloroform was added (200 μL). The mixture was incubated (5 min, RT) and centrifuged (4°C, 10 min, 13,000 × *g*). The aqueous upper phase was isolated and mixed with isopropanol (500 μL) without pipetting but rather snapping against the tube, thereby avoiding RNA shearing into small fragments. The solution was centrifuged (4°C, 10 min, 13,000 × *g*). The supernatant was discarded and the pellet was carefully resuspended in ethanol (75%, 1 mL), without pipetting. The samples were centrifuged (4°C, 10 min, 15,000 × *g*). The washing step with ethanol was repeated. The pellet was air dried (2 min) and resuspended in RNase-free water by snapping against the tube (50 μL). An additional bead-based purification step (Agencourt RNAclean XP kit, A63987; Beckman Coulter) including two further rounds of pellet washing with ethanol (75%) was incorporated. The purified RNA was resuspended in nuclease-free water (60 μL). The Qubit fluorometer and NanoDrop spectrophotometer were used for assessing RNA concentration and purity.

### Poly(A) tailing.

Bacterial RNA was poly(A) tailed with the E. coli poly(A) polymerase (New England BioLabs [NEB]; M0276) kit. After the final incubation (37°C, 30 min), an additional bead-based purification step including two further rounds of pellet washing with ethanol (75%) was performed with a final pellet resuspension in 12 μL of nuclease-free water. The Qubit fluorometer and NanoDrop spectrophotometer were used for assessing RNA concentration and purity, and the technical replicates were pooled to achieve a final poly(A)-tailed RNA concentration of 800 ng per sample in 9 μL of nuclease-free water.

### Library preparation and Nanopore sequencing.

Oxford Nanopore Technologies’ library preparation protocol for direct RNA sequencing (SQK-RNA002) was followed as recommended. Human enolase mRNA (110 nM, 0.5 μL) was spiked into the bacterial RNA samples. The libraries (200 ng of RNA) were loaded onto MinION flow cells (FLO-MIN106, R9.4 SpotON) and run for a minimum of 8 h. RNA was sequenced in the 3′-to-5′ direction.

### Computer and server requirements.

All computation was performed on the internal MHH HPC-seq 1000 core Ubuntu Linux compute cluster using software described at https://github.com/colindaven/guppy_on_slurm. Due to the intensive nature of base-calling, we strongly recommend using a GPU. A single Compute Unified Device Architecture (CUDA)-capable GPU is more energy efficient and far quicker than an entire compute cluster for this purpose, generating data in minutes instead of days.

### cDNA and RNA data analysis.

The differential expression analysis between cDNA 0-TEX, TEX, and ONT data sets was performed with the R function DESeq2 (55), which involves the following three steps: normalization of raw read counts by applying the median ratio method across biological replicates, followed by dispersion estimation and negative binominal general linear model fitting with Wald significance testing (56). Thereafter, empirical Bayes shrinkage of log_2_ fold change was applied to improve the fold change estimates and extract pairwise platform comparisons (57).

The high-accuracy base-calling of Nanopore raw signals and the automatic flipping of reads from 3′-to-5′ toward 5′-to-3′ direction was performed with Guppy (version 3.6.1 + 249406c, client-server API version 1.1.0 [https://community.nanoporetech.com]). The read quality was assessed with FASTQC 0.119 (58), and adapters were trimmed with Porechop 0.2.4.-2 (59). The reads were aligned against the P. aeruginosa NN2, P. aeruginosa SG17M, and enolase reference sequences with minimap2 2.15-r905 (60). Mapped reads were assigned to genomic features by approaching the R function *featureCounts* of Rsubread 2.6.4 (61) and by distinguishing between antisense (strandSpecific = 2) and sense (strandSpecific = 1) transcription. Raw RNA read counts were normalized with the weighted trimmed mean of M-values (TMM) method (62) to account for library size variations between samples and replicates. Afterwards, log_2_-scaled counts per million (CPM) were obtained with the R function cpm of edgeR 3.34.0 (63). Further details in relation to statistical testing and analysis are provided in the corresponding figure legends. All R scripts are publicly available.

### Generation of the SG17M genome sequence.

DNA was isolated from SG17M bacteria grown in LB until early stationary phase, according to the TRIzol DNA extraction protocol (Invitrogen; 15596026). For short-read DNA sequencing, aliquots were directly loaded onto a COVARIS S220 Focused-ultrasonicator to induce DNA fragmentation. Afterwards, the DNA was purified with AMPure XP beads (Beckman Coulter; A63881), and the NEBNext Ultra II DNA library prep kit (E7645, E7103) protocol was used. The DNA library was sequenced on the Illumina NextSeq 550 platform (high-output kit v2.5, 300 cycles, 20024908), so that approximately 21.9 million (1.8 Gb) forward and 21.9 million (1.7 Gb) reverse reads were generated. For Nanopore sequencing, the DNA obtained after cell lysis underwent direct AMPure XP bead purification. Replicates were pooled to achieve DNA concentrations of 1 μg in 47 μL of nuclease-free water. Oxford Nanopore Technologies’ library preparation protocol SQK-LSK109 was followed as recommended, and the library was loaded onto a MinION flow cell (FLO-MIN106). About 19.3 million reads were generated (3.9 Gb) and base-called with Guppy. The read quality was assessed with FastQC, and adapters were removed with Trimmomatic 0.39 (64) and Porechop. The Nanopore reads were assembled with flye 2.3 (65). Illumina reads were used for polishing the circular construct with pilon 1.23 (66). The genome completeness was assessed with BUSCO 4.1.2, a tool that verifies the presence of single-copy orthologs based on the lineage data set of *Pseudomonadales* (33).

### Data availability.

Coding scripts are publicly available from Github (https://github.com/mmpust/direct-RNAseq-2021). FAST5 files have been submitted to the public domain of the ENA (PRJEB46647). The complete reference genome of SG17M can be obtained from the National Center for Biotechnology Information (NCBI; accession numbers CP080369 and PRJNA749924).
