# Supplementary material for: Direct RNA Nanopore Sequencing of Pseudomonas aeruginosa Clone C Transcriptomes
Source: J Bacteriol. 2022 Jan 18;204(1):e00418-21. doi: 10.1128/JB.00418-21 (PMC8765398; doi:10.1128/JB.00418-21)
Supplement: Supplemental file 1 — Fig. S1 to S10 and Tables S1 to S3. Download JB.00418-21-s0001.pdf, PDF file, 5.9 MB [file jb.00418-21-s0001.pdf]

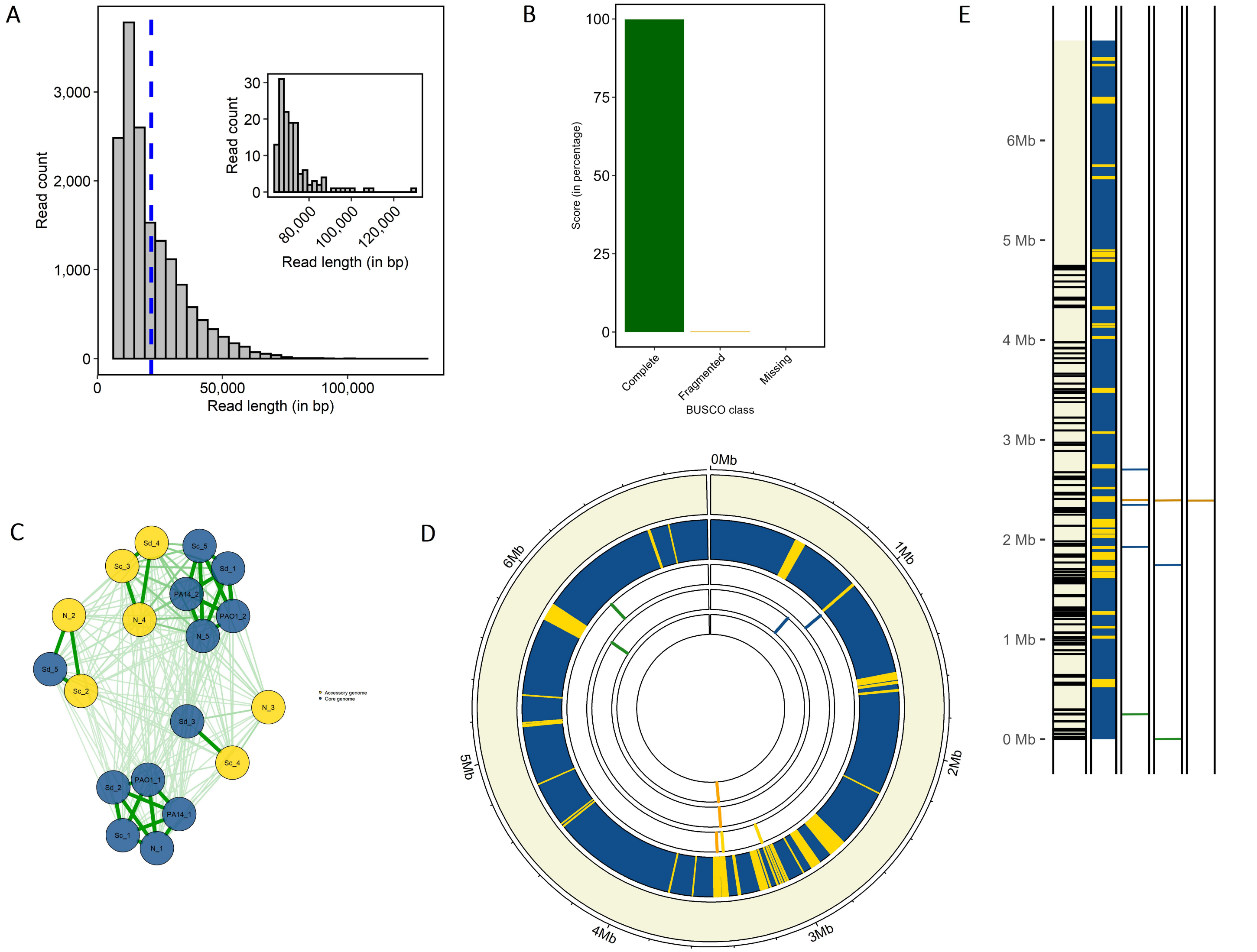

Supplementary Figure S1. The *P. aeruginosa* SG17M genome. (A) Evaluation of the SG17M genome assembly with flye. For the *de novo* assembly procedure only nanopore DNA reads of the SG17M strain with a minimum read length of 8,000 bp were selected. **(B)** After polishing the circular contig with paired-end Illumina reads, a BUSCO completeness score of 99.9 % was obtained. **(C) Network analysis of the sequence similarity** of ftsH observed in the following *P. aeruginosa* genomes: SG17M complete (SG17Mc), SG17M draft (SG17Md), NN2 (N2), PAO1 and PA14. The blue and yellow color represent *ftsH* gene locations in the core and accessory genome, respectively. **(D, E)** Structural overview of the publicly available and the newly generated SG17M reference DNA sequence. **(E)** Illustration of the linear SG17M draft and **(D)** the circularized complete genome. The left or in case of the circular genome the outer lane (beige) show the number and distribution of observed assembly gaps (black) in the genome structure. The second lane/inner circle (blue) visualizes the accessory genome (yellow) as predicted with IslandViewer4 (51). The third lane/inner circle provides information on the genome positions of *ftsH* genes in the core (green or blue) or accessory (yellow or orange) genome. The green and orange *ftsH* position correspond to the previously reported locations of the two clone C specific *ftsH* genes in the core and accessory genome, respectively. The two *ftsH* genes exhibit a sequence similarity of 45 % (36). The fourth lane/inner circle represents the genome positions of the detected core *clpG* gene (green), the accessory $clpG_{GI}$ gene (orange) and *clpB* (blue).The fifth lane/innermost circle shows the map position of the heat shock gene *sHscp20c*.

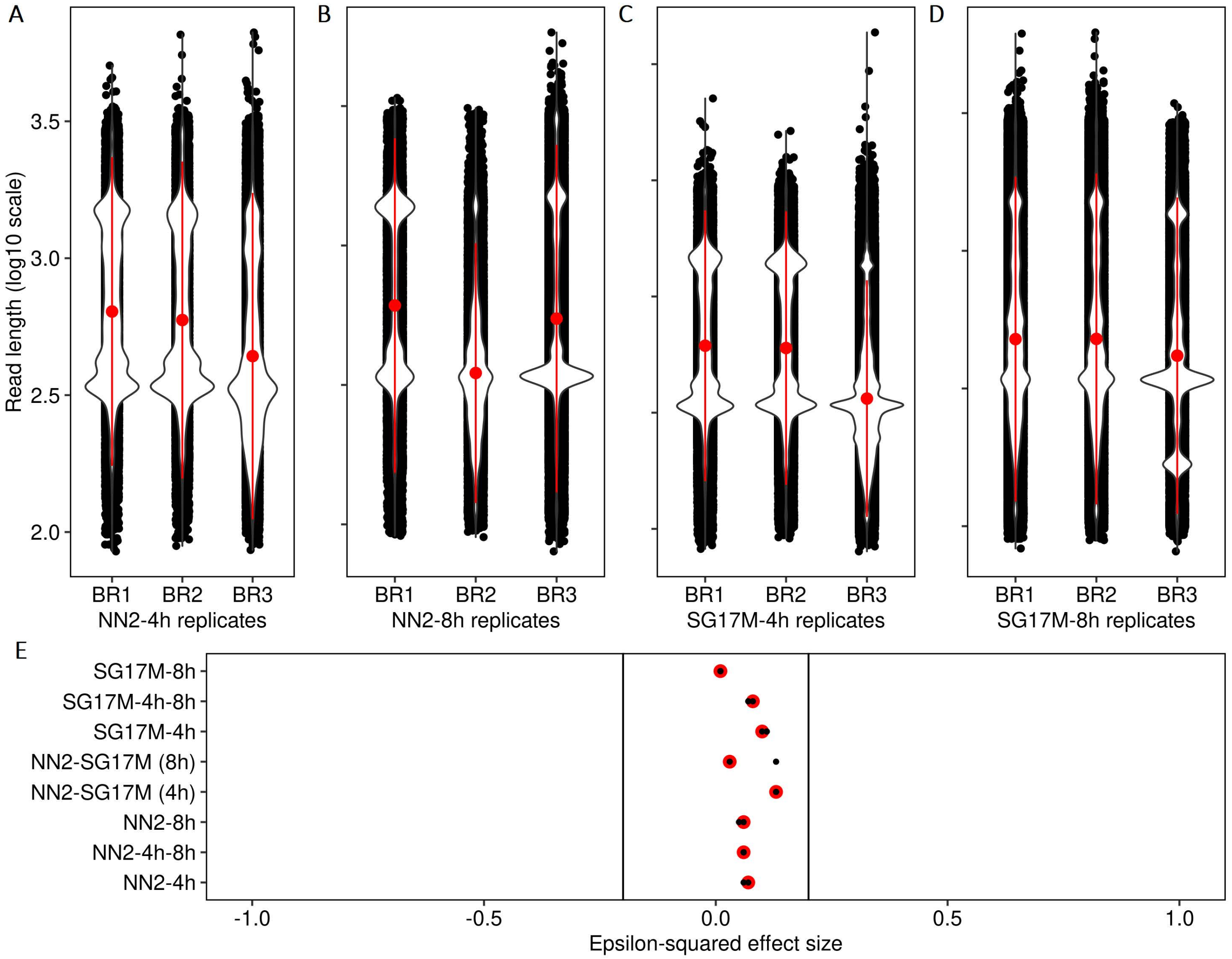

Supplementary Figure S2. Read length comparison between replicates, strains and time points. (A) Comparison between NN2 replicates at the mid-exponential (4h) growth phase. **(B)** Comparison between NN2 replicates at the early-stationary growth phase. **(C)** Comparison between SG17M replicates at the mid-exponential growth phase. **(D)** Comparison between SG17M replicates at the early-stationary growth phase. **(A) – (D)** The red dot and red lines reveal the mean and standard deviation. **(E)** The epsilon-squared effect size was always below 0.2, indicating none or only small variations in read length. The red dot represents the confidence intervals of the effect size.

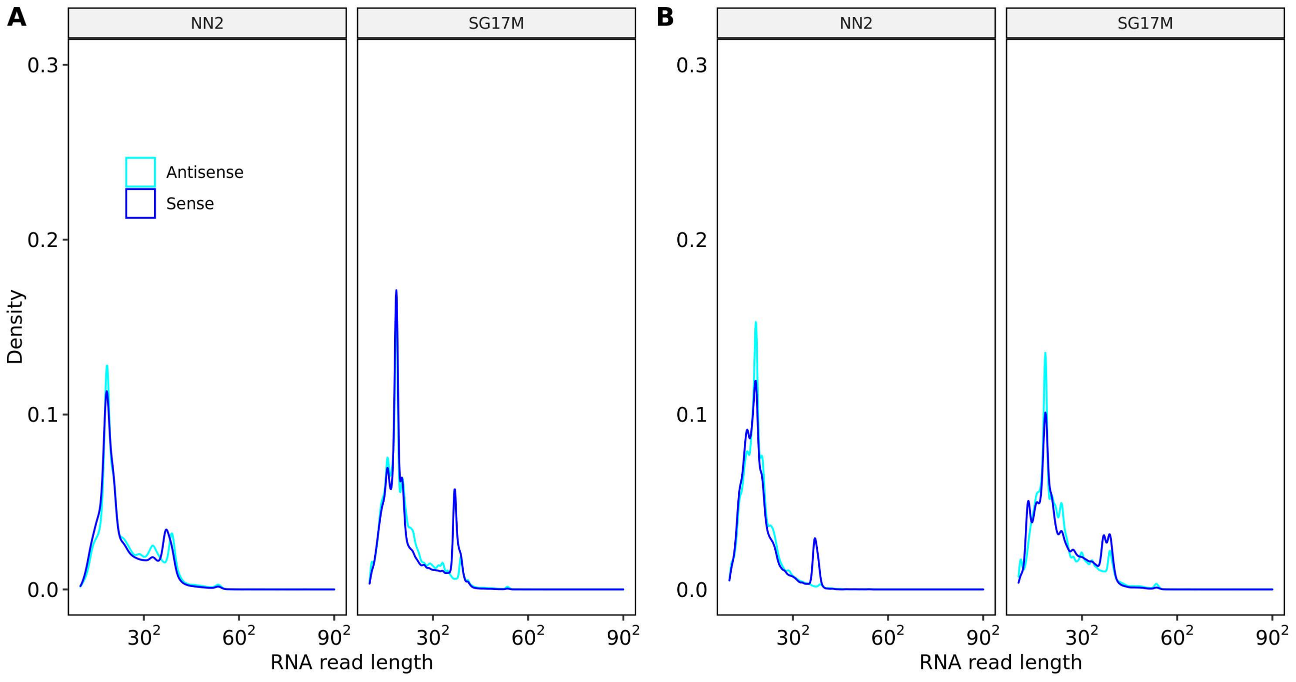

Supplementary Figure S3. Read length distribution between sense and antisense transcripts. (A) Comparison of sense and antisense transcript length at the mid-exponential phase in NN2 and SG17M. The observed difference in terms of effect size was neglectable (NN2: Wilcoxon r = 0.04 [0.02-0.06]; SG17M: Wilcoxon r = 0.03 [0.01-0.05]). **(B)** Comparison of sense and antisense transcript length at the early-stationary growth phase in NN2 and SG17M. The observed difference in terms of effect size was neglectable (NN2: Wilcoxon r = 0.05 [0.03-0.07]; SG17M: Wilcoxon r = 0.03 [0.0-0.04]).

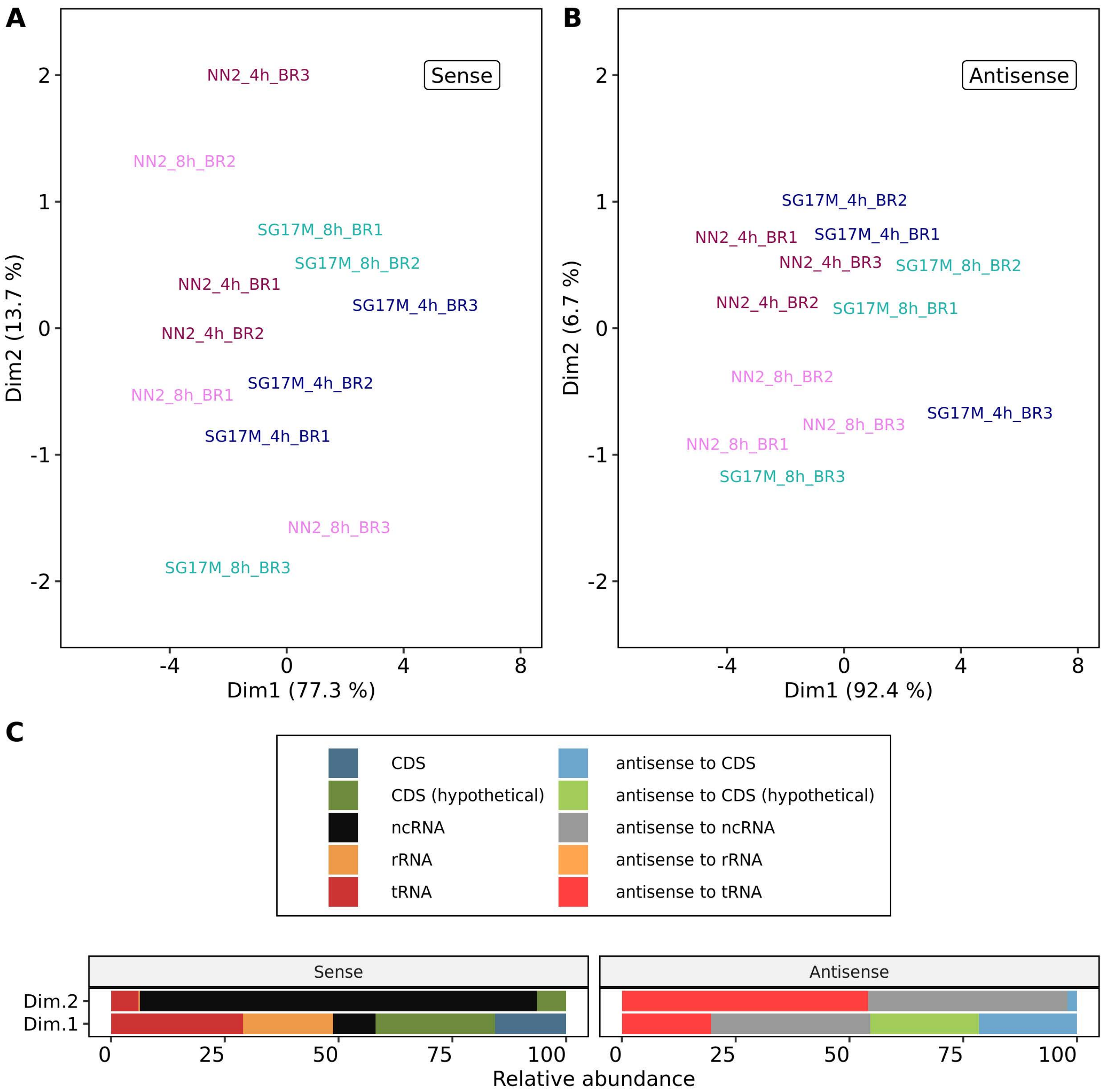

Supplementary Figure S4. Principal component analysis (PCA) for investigating the observed variance in sense (left) and antisense (right) transcript alignments to five genomic features between biological replicates. (A) PCA of sense transcript alignments. No strain- or time-specific signature was apparent. **(B) PCA of antisense transcript alignments.** NN2 replicates cluster close together and NN2 mid-exponential (4h) and early-stationary phase (8h) replicates are clearly separated by the second dimension. (**C) Contribution of variables to the separation** in the first dimension (Dim.1) and second dimension (Dim.2) for sense (left) and antisense (right) transcript alignment. For sense and antisense alignment, more than 50 % of the explained variance in the first dimension is associated with non-coding RNA. However, as shown in Figure 2, only a small proportion of non-coding RNA is found in the antisense read population. For antisense alignment, about 50 % of the observed variance is explained by tRNA and ncRNA pattern. This is what also separates the NN2-mid-exponential samples from the NN2 early-stationary-samples.

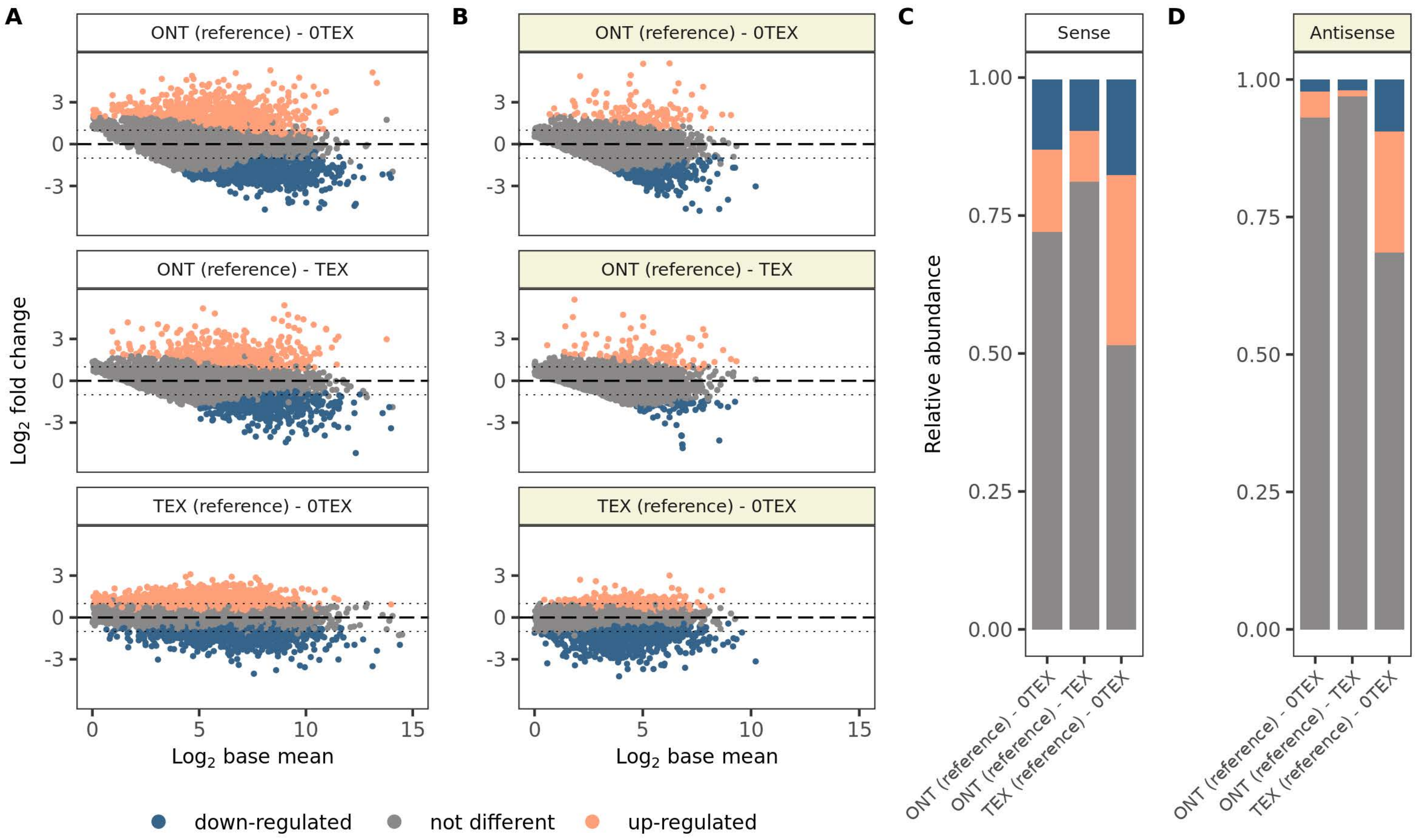

Supplementary Figure S5. Differential expression analysis between Illumina (TEX, 0TEX) and nanopore direct RNA-seq (ONT) data. (A) Comparison of sense transcription between (upper) ONT-NN2 and 0TEX-NN2, (middle) ONT-NN2 and TEX-NN2, (lower) TEX-NN2 and 0TEX-NN2. The blue, grey and orange dots represent down-regulated, similarly expressed or upregulated genomic features in ONT-NN2 samples. **(B)** Comparison of antisense transcription between (upper) ONT-NN2 and 0TEX-NN2, (middle) ONT-NN2 and TEX-NN2, (lower) TEX-NN2 and 0TEX-NN2. **(C)** Pairwise comparison of the relative abundance of genomic features characterized as down-regulated, not different and up-regulated in sense transcription. **(D)** Pairwise comparison of the relative abundance of genomic features characterized as down-regulated, not different and up-regulated in antisense transcription.

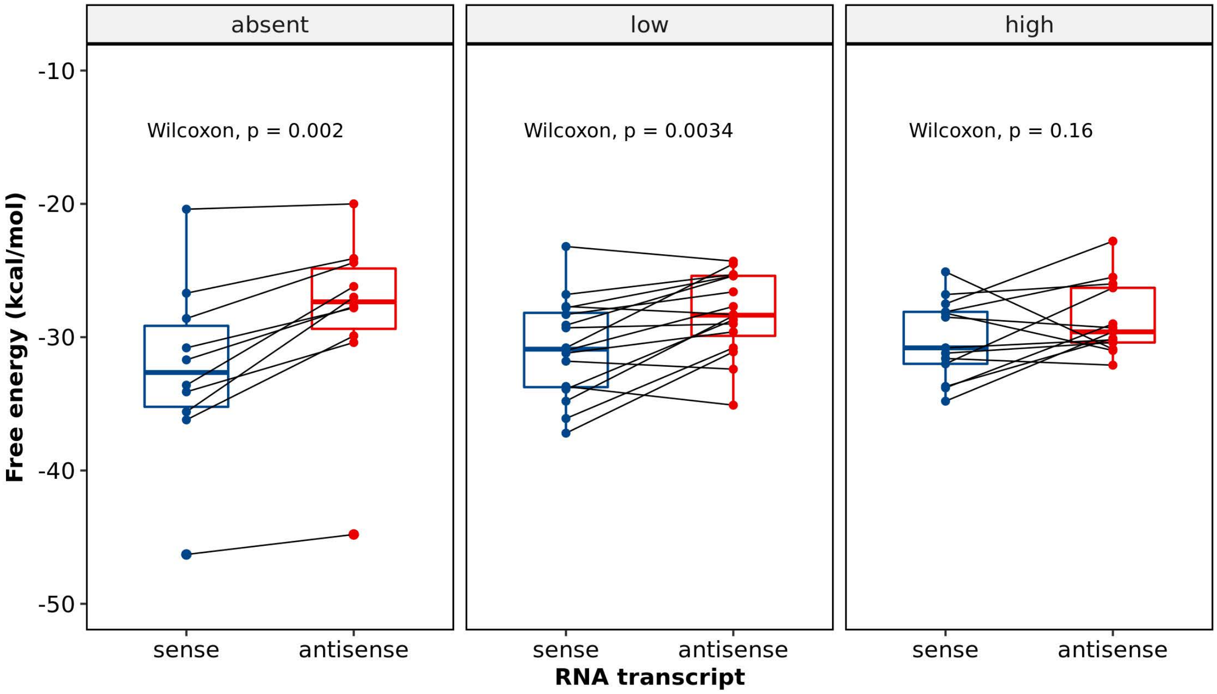

Supplementary Figure S6. Pairwise comparison of the predicted minimum free energy of folding (MFE) based on Zuker and Stiegler's algorithm (46) between sense-antisense tRNA transcription. **(Left box, 'absent')** For 10 tRNA genes, no antisense transcripts were detected. RNAfold was run to obtain the MFE prediction for sense and antisense sequences. In general, the MFE of the predicted sense secondary structure was significantly lower than the MFE of the corresponding antisense structure. **(Centre box, 'low')** For 16 tRNA genes, low antisense transcription was detected (< 10 reads per entity). In this case, the MFE of the predicted sense secondary structure was also typically lower compared to the partner antisense entity. (**Right box, 'high')** 13 high abundant tRNA entities were detected. Here, the MFE of the predicted sense and antisense secondary structure was comparable.

A) NN2:4721872-4721947 (tRNA-LysTTT)
5'GGGTCGTTAGCTCAGTCGGTAGAGCAGTTGGCTTTTAACCAATTGGTCGTAGGTTCGAATCCTACACGACCCACCA

B) NN2:5577611-5577710 (tRNA-LysTTT-pseudo)
5'AGCTAAATCGGTACGGGGCTTTAAAACCCTTCCTAGAGAGACCTAGGCCAATTGTCTTTTAACCAATTGGTCGTAGGTTCGAATCCTACACGACCCACCA

C) NN2:5704205-5704280 (tRNA-LysTTT)
5'GGGTCGTTAGCTCAGTCGGTAGAGCAGTTGGCTTTTAACCAATTGGTCGTAGGTTCGAATCCTACACGACCCACCA

Pseudomonas aeruginosa strain C plasmid pKLC102, complete sequence
Sequence ID: AY257538.1 Length: **103532** Number of Matches: **1**

Score	Expect	Identities	Gaps	Strand
185 bits(100)	4e-43	100/100(100%)	0/100(0%)	Plus/Minus

```
Query  1    AGCTAAATCGGTACGGGGCTTTAAAACCCTTCCTAGAGAGACCTAGGCCAATTGTCTTTT  60
            ||||||||||||||||||||||||||||||||||||||||||||||||||||||||||||
Sbjct  100  AGCTAAATCGGTACGGGGCTTTAAAACCCTTCCTAGAGAGACCTAGGCCAATTGTCTTTT  41

Query  61   AACCAATTGGTCGTAGGTTCGAATCCTACACGACCCACCA  100
            ||||||||||||||||||||||||||||||||||||||||
Sbjct  40   AACCAATTGGTCGTAGGTTCGAATCCTACACGACCCACCA  1
```

FASTA searches a protein or DNA sequence data bank version 36.3.8h Aug, 2019. W.R. Pearson & D.J. Lipman PNAS (1988) 85:2444-2448 .
Obtained from https://blast.ncbi.nlm.nih.gov/Blast.cgi#1992576687 (last accessed 18 October 2021).

Supplementary Figure S7. Comparison of tRNA-Lys-anticodon-TTT regions in the NN2 genome. (A-C) Location of three Lys-TTT regions in the NN2 genome. A BLAST search of the tRNA-LysTTT-pseudo sequence revealed a match (100 % identity) with the pKLC102 plasmid.

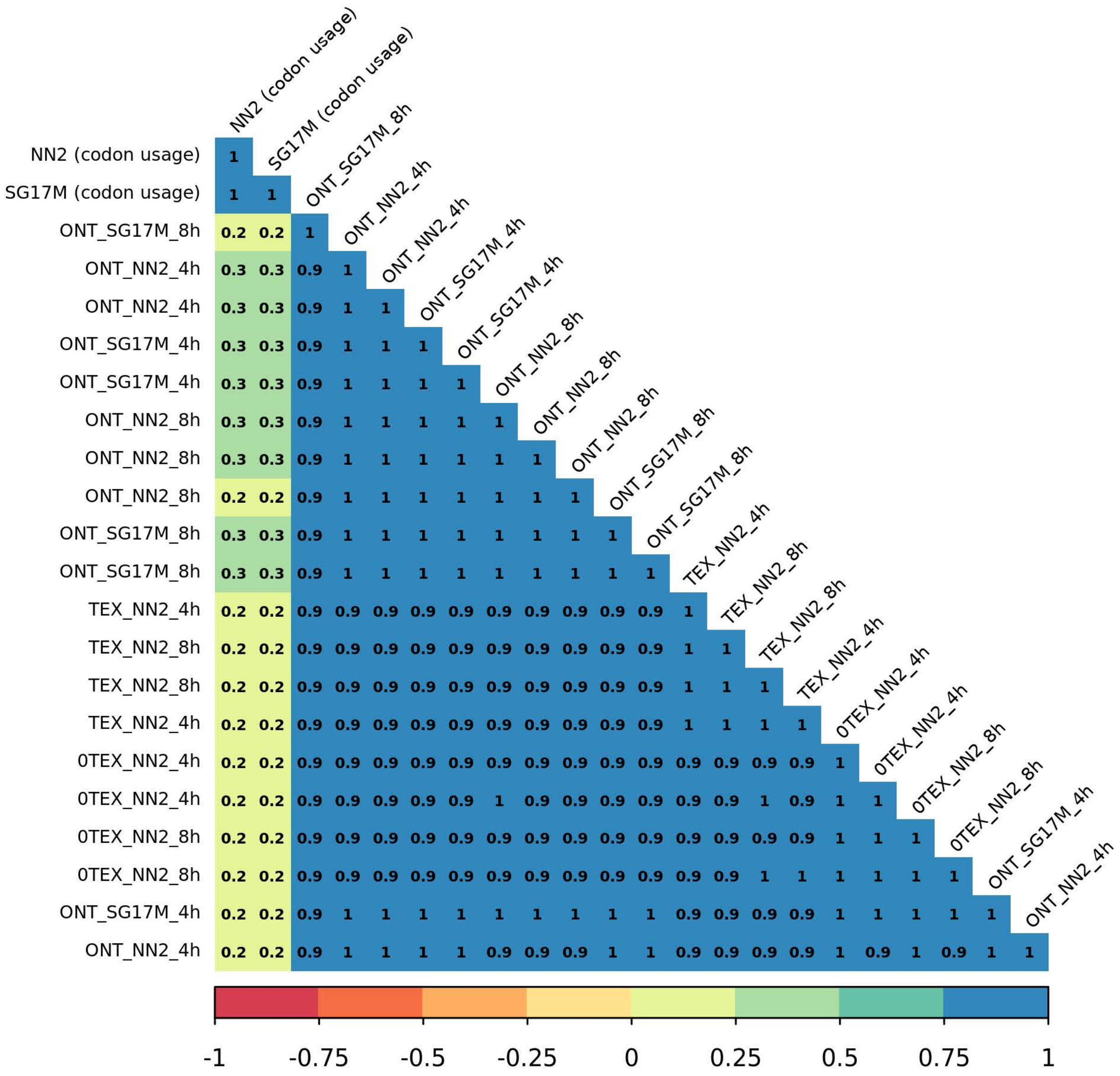

Supplementary Figure S8. Spearman's rank-order correlation analysis of the theoretical codon usage obtained from either the NN2 (NN2 codon usage) or SG17M (SG17M codon usage) genome sequence and tRNA abundance of the biological samples. The numeric Spearman's rank correlation coefficient (value between -1 and 1) is provided in the plot. The dark blue or dark red color represents a strong positive or a strong negative correlation, respectively.

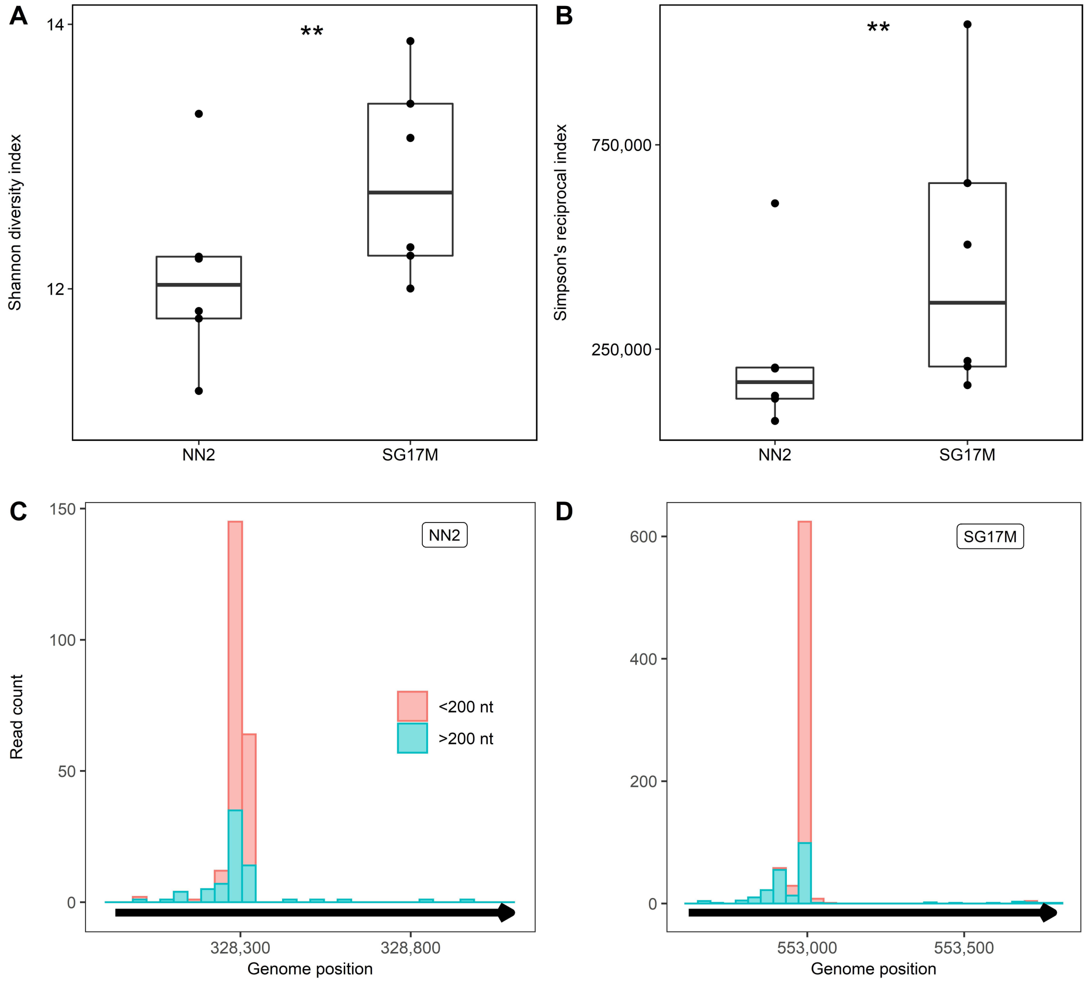

Supplementary Figure S9. Comparing of the diversity of antisense transcript start positions between NN2 and SG17M. (A) Shannon diversity of transcript starting sites (Wilcoxon p-value < 0.01, effect size r = 0.57, CI = 0.24 – 0.8) and **(B)** the Simpson's reciprocal index of starting sites (Wilcoxon p-value < 0.01, effect size r = 0.57, CI = 0.24 – 0.8) was significantly higher in SG17M compared to NN2, indicating higher diversity of starting sites in SG17M and increased dominance of specific read positions in NN2, respectively. Antisense transcript starting positions within the intQ gene. **(C)** Starting sites observed in NN2 replicates and (D) starting sites observed in SG17M replicates.

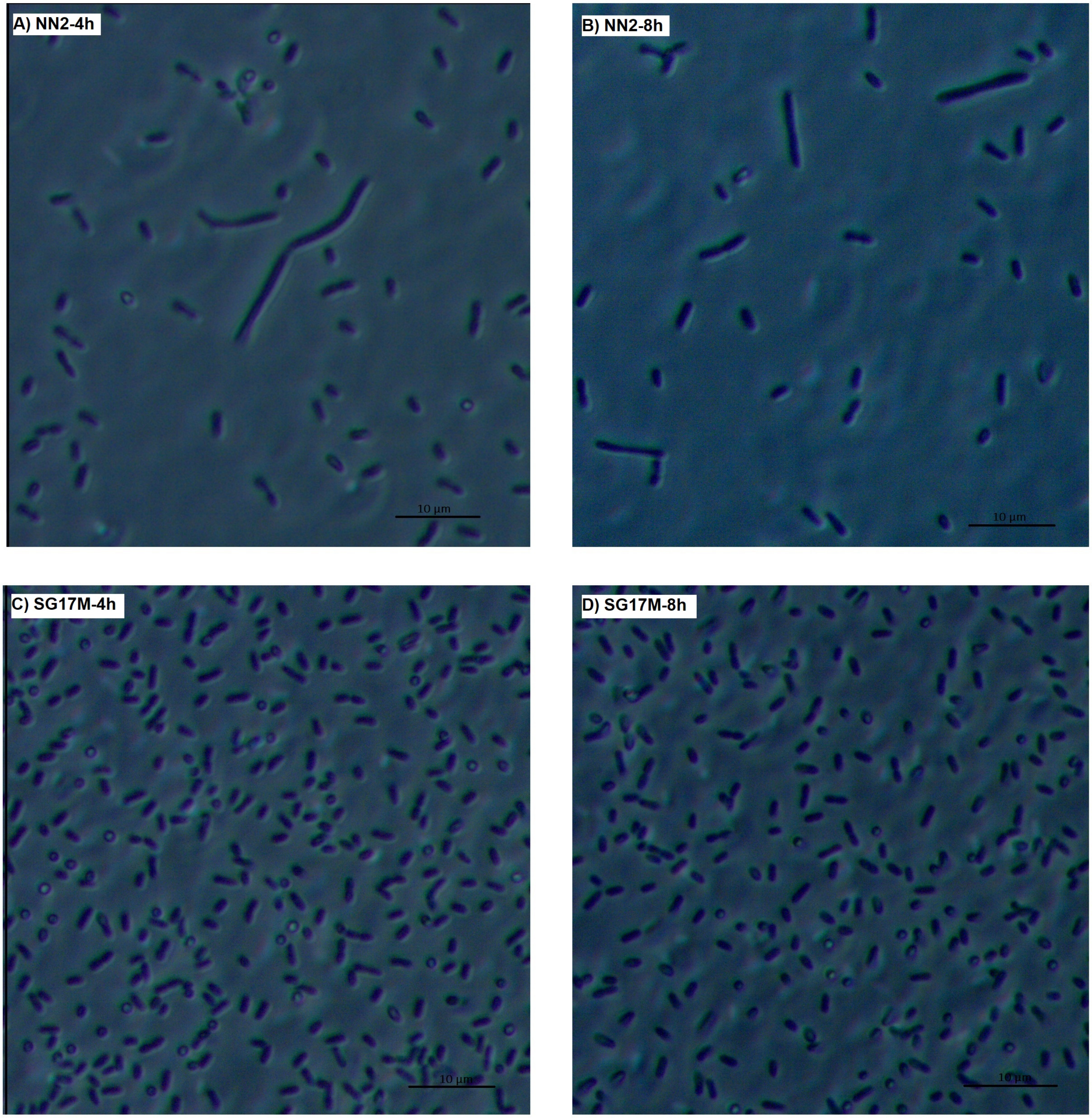

Supplementary Figure S10. Light microscopy images of planktonically growing clone C bacteria: (upper left) NN2-mid exponential, (upper right) NN2-early stationary, (lower left) SG17M-mid exponential and (lower right) SG17M-early stationary growth phase.

Bacteria

1st Base		A		G		U		C		3rd Base
	A	AAA	Phe	AGA	Ser	AUA	Tyr	ACA	Cys	A
	G	GAA	Phe	GGA	Ser	GUA	Tyr	GCA	Cys	
	U	UAA	Leu	UGA	Ser	UUA	Stop	UCA	Stop	
	C	CAA	Leu	CGA	Ser	CUA	Stop	CCA	Trp	
	A	AAG	Leu	AGG	Pro	AUG	His	ACG	Arg	G
	G	GAG	Leu	GGG	Pro	GUG	His	GCG	Arg	
	U	UAG	Leu	UGG	Pro	UUG	Gln	UCG	Arg	
	C	CAG	Leu	CGG	Pro	CUG	Gln	CCG	Arg	
	A	AAU	Ile	AGU	Thr	AUU	Asn	ACU	Ser	U
	G	GAU	Ile	GGU	Thr	GUU	Asn	GCU	Ser	
	U	UAU	Ile	UGU	Thr	UUU	Lys	UCU	Arg	
	C	CAU	Met	CGU	Thr	CUU	Lys	CCU	Arg	
	A	AAC	Val	AGC	Ala	AUC	Asp	ACC	Gly	C
	G	GAC	Val	GGC	Ala	GUC	Asp	GCC	Gly	
	U	UAC	Val	UGC	Ala	UUC	Glu	UCC	Gly	
	C	CAC	Val	CGC	Ala	CUC	Glu	CCC	Gly	

Table S1. This representation of missing tRNA anticodon alternatives was obtained and modified from Figure 2 of Ehrlich et al. (2021). The blue colour depicts missing tRNA isoacceptors as reported by Ehrlich et al. (2021) based on their investigations of more than 100 bacterial species, including Acidobacteria, Actinobacteria, Aquificae, Bacteroidetes, Chlamydiae, Chlorobi, Chloroflexi, Cyanobacteria, Deinococcus-Thermus, Firmicutes, Tenericutes, Thermodesulfobacteria, and Thermotogae phyla. The blue-coloured genes were also missing in *Pseudomonas aeruginosa* NN2 and SG17M reference sequences. Importantly, four other tRNA genes (red colour) were also missing. Please note that Ehrlich and colleagues did not compile data from proteobacteria.

Supplementary Table S2. Overview of the antisense tRNAs observed in clone C isolates with direct nanopore sequencing. For the 37 anticodon alternatives (without stop codons, see Supplementary Table 1), antisense tRNA expression was categorised into the following three groups: High expression of antisense tRNA (> 10 reads), low expression of antisense tRNA (< 10 reads) and no expression (< 1 read). The optimal secondary structure of sense tRNAs and their reverse complement sequence (antisense) was predicted with RNAfold 2.4.18 [1]. The minimum free energy of folding (MFE) based on Zuker and Stiegler's algorithm [2], and the ensembly diversity (ED) were calculated. As recently summarised by Sato and colleagues (2021), MFE is based on a thermodynamic model, in which a secondary structure is decomposed into sub-structures (nearest-neighbour loops), including hairpin loops, internal and external loops, or base-pair stacking. The free energy of every decomposed nearest-neighbour loop is calculated and summed to obtain the total free energy of the RNA structure [3]. Finally, the optimal secondary structure is defined by the construct containing the lowest free energy [2, 3]. The ensemble diversity quantifies the similarity of all predicted secondary structures from one sequence by computing pair probabilities [2].

tRNA gene category	tRNA-anticodon	transcript-type	MFE (kcal/mol)	ED	tRNA sequence (5'-3' direction)	Secondary structure in dot-bracket notation [1]
high	Lys-TTT	sense	-28.1	10.3	GGGTCGTTAGCTCAGTCGGTAGAGCAGTTGGCTTTTAACCAATTGGTCGTAGGTTCGAATCCTACACGACCCACCA	(((((((..(((.....))).((.(((((((.......))))))).))(((((.......)))))))))))))....
high	Lys-TTT-pKLC102	sense	-25.1	22.5	AGCTAAATCGGTACGGGGCTTTAAAACCCTTCCTAGAGAGACCTAGGCCAATTGTCTTTTAACCAATTGGTCGTAGGTTCGAATCCTACACGACCCACCA	.........(((.(((((........)))..(((((......)))))((((((.........)))))))..(((((.......))))).)))))....
high	Lys-TTT	antisense	-25.5	4.06	TGGTGGGTCGTGTAGGATTCGAACCTACGACCAATTGGTTAAAAGCCAACTGCTCTACCGACTGAGCTAACGACCC	....((((((((((((.......)))))......((((((...))))))..((((........))))..)))))))
high	Lys-TTT-pKLC102	antisense	-30.9	10.4	TGGTGGGTCGTGTAGGATTCGAACCTACGACCAATTGGTTAAAAGACAATTGGCCTAGGTCTCTCTAGGAAGGGTTTTAAAGCCCCGTACCGATTTAGCT	.(((.(((((((((((.......)))))).(((((((.........)))))))((((((....))))))..((((((....))))))).))).)))....
high	Thr-TGT	sense	-27.5	10.6	GCCGGTATAGCTCAGCTGGTAGAGCAACTGACTTGTAATCAGTAGGTCCCGGGTTCGACTCCTGGTGCCGGCACCA	(((((((..((((........)))).(((((.......)))))).....(((((.......)))))))))))))....
high	Thr-TGT	antisense	-22.8	11.5	TGGTGCCGGCACCAGGAGTCGAACCCGGGACCTACTGATTACAAGTCAGTTGCTCTACCAGCTGAGCTATACCGGC	....(((((........................(((((((...))))))).((((........))))....)))))
high	Ala-TGC	sense	-31.2	16.3	GGGGCCATAGCTCAGCTGGGAGAGCGCCTGCTTTGCACGCAGGAGGTCAGGAGTTCGATCCTCCTTGGCTCCACCA	(((((((..((((........))))((.(((...))).))((((((((........)).))))))))))))))....
high	Ala-TGC	antisense	-30.4	3.6	TGGTGGAGCCAAGGAGGATCGAACTCCTGACCTCCTGCGTGCAAAGCAGGCGCTCTCCCAGCTGAGCTATGGCCCC	....((.((((((((((.(((......))))))))))((.(((...))).))((((........))))..))))))).
high	Ile-GAT	sense	-31.6	11.1	GGGTCTGTAGCTCAGTTGGTTAGAGCGCACCCCTGATAAGGGTGAGGTCGGCAGTTCGAATCTGCCCAGACCCACCA	(((((((..((((.........)))).(((((.......)))))).....(((((.......))))))))))))....
high	Ile-GAT	antisense	-32.1	1.1	TGGTGGGTCTGGGCAGATTCGAACTGCCGACCTCACCCTTATCAGGGGTGCGCTCTAACCAACTGAGCTACAGACCC	....((((((((((((.......)))))).....(((((((...))))))).((((.........))))..)))))))
high	Asp-GTC	sense	-33.8	3.6	GCAGCGGTAGTTCAGTCGGTTAGAATACCGGCCTGTCACGCCGGGGGtCGCGGGTTCGAGTCCCGTCCGCTGCGCCA	(((((((..((.((((((((......))))).))).)).)).(((((.(((......))).))))).)))))))....
high	Asp-GTC	antisense	-29	5.8	TGGCGCAGCGGACGGGACTCGAACCCGCGACCCCCGGCGTGACAGGCCGGTATTCTAACCGACTGAACTACCGCTGC	....(((((((.((((..(((......)))..)))))..((..(((..((((......)))).))).)).)))))))
high	Gly-TCC	sense	-28.2	4.17	GCGGGTATAGTTCAGTGGTAGAACCTCAGCCTTCCAAGCTGATGATGCGGGTTCGATTCCCGCTACCCGCTCCA	(((((((..((((.......)))).(((((.......)))))....(((((.......))))))))))))....
high	Gly-TCC	antisense	-31	1.5	TGGAGCGGGTAGCGGGAATCGAACCCGCATCATCAGCTTGGAAGGCTGAGGTTCTACCACTGAACTATACCCGC	....((((((((((((.......)))))....(((((((...)))))))(((((.......))))).)))))))
high	Tyr-GTA	sense	-28.5	2.6	GGAGGGATTCCCGAGTGGCCAAAGGGATCAGACTGTAAATCTGACGTCATAGACTTCGAAGGTTCGAATCCTTCTCCCTCCACCA	(((((((.((((...........))))(((((.......))))).(((...)))....(((((.......))))))))))))....
high	Tyr-GTA	antisense	-29.3	2.1	TGGTGGAGGGAGAAGGATTCGAACCTTCGAAGTCTATGACGTCAGATTTACAGTCTGATCCCTTTGGCCACTCGGGAATCCCTCC	....((((((((((((.......)))))...(((...)))((((((((...))))))))(((...........)))..)))))))
high	Ser-TGA	sense	-33.7	17.2	GGAGGTGTGGCCGAGTGGTTTAAGGCAACGGTCTTGAAAACCGTCGAAGGGGAGACTCTTCCGTGAGTTCGAATCTCACCGCCTCCGCCA	(((((((..(((...........))).(((((.......))))).((((((....)))))).(((((.......)))))))))))))....
high	Ser-TGA	antisense	-30.1	7.2	TGGCGGAGGCGGTGAGATTCGAACTCACGGAAGAGTCTCCCCTTCGACGGTTTTCAAGACCGTTGCCTTAAACCACTCGGCCACACCTCC	((((.(((..((((((((((............)))))))......(((((((((...)))))))))......))).))).))))).......
high	Pro-TGG	sense	-32	2.14	CGGGGTATAGCGCAGTCCGGTAGCGCGCCTGCTTTGGGAGCAGGATGTCGGGAGTTCGAATCTCTCTACCCCGACCA	(((((((..((((.........)))).(((((((...)))))))......(((((.......))))))))))))....
high	Pro-TGG	antisense	-26.3	12.4	TGGTCGGGGTAGAGAGATTCGAACTCCCGACATCCTGCTCCCAAAGCAGGCGCGCTACCGGACTGCGCTATACCCCG	..((((((((.((.....))..)).)))))))..((((((.....)))))).((((.........))))).........
high	Leu-CAG	sense	-34.8	7.98	GCCCAGGTGGCGGAATTGGTAGACGCACTAGGTTCAGGTCCTAGCGGTGGCAACACCGTGGAAGTTCGAGTCTTCTCCTGGGCACCA	(((((((..(((...........))).(((((.......)))))(((((....))))).(((((.......))))))))))))....
high	Leu-CAG	antisense	-29.6	4.02	TGGTGCCCAGGAGAAGACTCGAACTTCCACGGTGTTGCCACCGCTAGGACCTGAACCTAGTGCGTCTACCAATTCCGCCACCTGGGC	....(((((((.((((.......))))..(((.((((..((((((((.......))))))).))....)))).)))...)))))))
high	Asn-GTT	sense	-26.8	8.4	TCCGCGATAGCTCAGTCGGTAGAGCAAATGACTGTTAATCATTGGGTCCCTGGTTCGAGTCCAGGTCGCGGAGCCA	(((((((..(((((((.(((..((((......)))).)))))))))).(((((.......))))))))))))....
high	Asn-GTT	antisense	-26	2.16	TGGCTCCGCGACCTGGACTCGAACCAGGGACCCAATGATTAACAGTCATTTGCTCTACCGACTGAGCTATCGCGGA	....((((((((((((.......))))).....(((((((...))))))).((((........))))..)))))))
high	Thr-GGT	sense	-30.8	1	GCTCATGTAGCTCAGTTGGTAGAGCACACCCTTGGTAAGGGTGAGGTCAGCGGTTCAAATCCGCTCATGAGCTCCA	(((((((..((((........)))).(((((((...)))))))......(((((.......)))))))))))))....
high	Thr-GGT	antisense	-30.2	0.79	TGGAGCTCATGAGCGGATTTGAACCGCTGACCTCACCCTTACCAAGGGTGTGCTCTACCAACTGAGCTACATGAGC	....((((((((((((.......)))))).....(((((((...))))))).((((........))))..)))))))
low	Leu-CAA	sense	-34.8	12	GCCTCGGTGGCGGAATCGGTAGACGCGGCGGATTCAAAATCCGTTTCTGGCGACAGAGTGAGAGTTCGAGTCTCTCCCGAGGCACCA	(((((((..(((...........)))((((((((...))))))))((((....))))..(((((.......))))))))))))....
low	Leu-CAA	antisense	-28.4	5	TGGTGCCTCGGGAGAGACTCGAACTCTCACTCTGTCGCCAGAAACGGATTTTGAATCCGCCGCGTCTACCGATTCCGCCACCGAGGC	....((((((((((((.......)))))..((((....))))..((((((...))))))..(((((....))...)))..)))))))
low	Phe-GAA	sense	-36.1	1.6	GCCCAGGTAGCTCAGTTGGTAGAGCAGGGGATTGAAAATCCCCGTGTCGGCGGTTCGATTCCGTCCCTGGGCACCA	(((((((..((((........)))).(((((((...)))))))......(((((.......))))))))))))....
low	Phe-GAA	antisense	-30.8	5	TGGTGCCCAGGGACGGAATCGAACCGCCGACACGGGGATTTTCAATCCCCTGCTCTACCAACTGAGCTACCTGGGC	....(((((((..(((.......)))........(((((((...))))))).((((........))))..)))))))

low	Gly-CCC	sense	-28.3	9.18	GCGGGCGTCGTATAATGGCATTACCTGAGCTTCCCAAGCTCATGACGAGGGTTCGATTCCCTTCGCCCGCTCCA	((((((((((((...)))))).......(((((((...)))))))....(((((.......))))).))))))....
low	Gly-CCC	antisense	-26.6	10	TGGAGCGGGCGAAGGGAATCGAACCCTCGTCATGAGCTTGGGAAGCTCAGGTAATGCCATTATACGACGCCCGC	....(((((((.((((.......)))).....(((((((...)))))))(((...)))..........)))))))
low	Arg-CCT	sense	-31.8	10.06	GTCCCGGTAGCTCAATTGGATAGAGCATCCCCCTCCTAAGGGGAAGGTTGGAGGTTCGACCCCTCTCCGGGACGCCA	(((((((..((((.........))))...(((((....)))))..((((((....))))))......)))))))....
low	Arg-CCT	antisense	-32.4	13.2	TGGCGTCCCGGAGAGGGGTCGAACCTCCAACCTTCCCCTTAGGAGGGGGATGCTCTATCCAATTGAGCTACCGGGAC	....(((((((.((((.......)))).....(((((((....))))))).((((.........))))..)))))))
low	Cys-GCA	sense	-23.2	17.4	GGCTGAGTAGCAGAGTGGTTATGCACCGGATTGCAAATCCGTGAACGCCGGTTCGATTCCGACCTCAGCCTCCA	(((((((...................((((((...))))))...((....))...........)))))))....
low	Cys-GCA	antisense	-24.3	10.1	TGGAGGCTGAGGTCGGAATCGAACCGGCGTTCACGGATTTGCAATCCGGTGCATAACCACTCTGCTACTCAGCC	....((((((((((((.......)))))).....((((((...))))))...................)))))))
low	Arg-CCG	sense	-33.9	9.03	GCACCAGTAGCTCAGCTGGATAGAGTACCGCCCTCCGAAGGCGGGGGTCGTGGGTTCGAATCCCGCCTGGTGCGCCA	(((((((.......((.((((.(((..((((((((((....))))))..)))).)))..)))).)))))))))....
low	Arg-CCG	antisense	-28.7	14	TGGCGCACCAGGCGGGATTCGAACCCACGACCCCCGCCTTCGGAGGGCGGTACTCTATCCAGCTGAGCTACTGGTGC	...((((((((((((..(((......)))..))))))(((((..((.((....))..))..)))))....))))))
low	His-GTG	sense	-26.8	2.8	GTGGGCGTAGCTCAGTTGGTAGAGCACAGGATTGTGGCTCCTGGTGTCGTGGGTTCGATTCCCATCGTCCACCCCA	(((((((..((((........)))).(((((.......)))))......(((((.......))))))))))))....
low	His-GTG	antisense	-25.3	13.2	TGGGGTGGACGATGGGAATCGAACCCACGACACCAGGAGCCACAATCCTGTGCTCTACCAACTGAGCTACGCCCAC	..(((((..((.((((.......))))))....(((((.......))))).((((........))))..)))))..
low	Met-CAT	sense	-27.8	2.9	CGCGGGATGGAGCAGTCTGGTAGCTCGTCGGGCTCATAACCCGAAGGTCGTTGGTTCAAATCCAGCTCCCGCAACCA	.((((((..((((.........)))).(((((.......)))))......(((((.......))))))))))).....
low	Met-CAT	antisense	-25.4	6.5	TGGTTGCGGGAGCTGGATTTGAACCAACGACCTTCGGGTTATGAGCCCGACGAGCTACCAGACTGCTCCATCCCGCG	.....((((((..(((.......)))........(((((((...))))))).((((.........))))..)))))).
low	Val-TAC	sense	-29.3	5.7	GGGTGATTAGCTCAGCTGGGAGAGCATCTGCCTTACAAGCAGAGGGTCGGCGGTTCGATCCCGTCATCACCCACCA	(((((((..((((........)))).(((((.......)))))(((((((....)))))))....)))))))....
low	Val-TAC	antisense	-29	1.5	TGGTGGGTGATGACGGGATCGAACCGCCGACCCTCTGCTTGTAAGGCAGATGCTCTCCCAGCTGAGCTAATCACCC	....(((((((...(((.(((......))))))(((((((...))))))).((((........))))..)))))))
low	Pro-GGG	sense	-30.8	5.3	CGGGGCGTAGCGCAGCCTGGTAGCGCACTTGCATGGGGTGCAAGGGGTCGAGTGTTCGAATCACTCCGTCCCGACCA	(((((((..((((.........)))).(((((((...))))))).....(((((.......))))))))))))....
low	Pro-GGG	antisense	-24.5	18.5	TGGTCGGGACGGAGTGATTCGAACACTCGACCCCTTGCACCCCATGCAAGTGCGCTACCAGGCTGCGCTACGCCCCG	....((((.(((((((.......)))))).....((((((.....)))))).((((.........))))..)).))))
low	Trp-CCA	sense	-31.2	13	AGGCCAGTAGCTCAATTGGCAGAGCGGCGGTCTCCAAAACCGCAGGTTGGGGGTTCGATTCCCTCCTGGCCTGCCA	(((((.((.((((........)))).))))))).........((((((((((((........)).))))))))))..
low	Trp-CCA	antisense	-29.6	6.4	TGGCAGGCCAGGAGGGAATCGAACCCCCAACCTGCGGTTTTGGAGACCGCCGCTCTGCCAATTGAGCTACTGGCCT	....(((((((..(((........)))......(((((((...))))))).((((........))))..)))))))
low	Arg-TCT	sense	-37.2	10.2	GCGCCCGTAGCTCAGCTGGATAGAGCATCCGCCTTCTAAGCGGATGGTCGCAGGTTCGAGTCCTGCCGGGTGCGCCA	((((((((((((...))))....((.(((((((.......))))))).))(((((.......))))))))))))))....
low	Arg-TCT	antisense	-31.1	9.5	TGGCGCACCCGGCAGGACTCGAACCTGCGACCATCCGCTTAGAAGGCGGATGCTCTATCCAGCTGAGCTACGGGCGC	....((.(((((((((.......)))))....((((((((...))))))))((((.........))))..)))).))
low	Arg-ACG	sense	-27.7	12.7	GCACTCATAGCTCAGCTGGATAGAGTACTCGGCTACGAACCGAGCGGTCGGAGGTTCGAATCCTCCTGAGTGCGCCA	(((((((..((((.........)))).(((((.......)))))......(((((.......))))))))))))....
low	Arg-ACG	antisense	-28.3	6.6	TGGCGCACTCAGGAGGATTCGAACCTCCGACCGCTCGGTTCGTAGCCGAGTACTCTATCCAGCTGAGCTATGAGTGC	....((((((((((((.......)))))....((((((((...)))))))).(((.........)))...)))))))
low	Ala-GGC	sense	-29.1	6.3	GGGGCTATAGCTCAGCTGGGAGAGCGCTTGCATGGCATGCAAGAGGTCGACGGTTCGATCCCGTCTAGCTCCACCA	(((((((..((((........)))).(((((((...))))))).....(((((.......))))))))))))....
low	Ala-GGC	antisense	-25.4	13.6	TGGTGGAGCTAGACGGGATCGAACCGTCGACCTCTTGCATGCCATGCAAGCGCTCTCCCAGCTGAGCTATAGCCCC	(((.(((((..((((((.......)))))).....(((((((...))))))).))))).)))((((.....))))...
low	Glu-TTC	sense	-33.7	4.2	GTCCCCTTCGTCTAGTGGCCTAGGACACCGCCCTTTCACGGCGGTAACAGGGGTTCGAGTCCCCTAGGGGACGCCA	(((((((..((((((....))).)))((((((.......))))))...(((((.......))))))))))))....
low	Glu-TTC	antisense	-35.1	4.7	TGGCGTCCCCTAGGGGACTCGAACCCCTGTTACCGCCGTGAAAGGGCGGTGTCCTAGGCCACTAGACGAAGGGGAC	....((((((((((((.......))))))..(((((((.......)))))))..((((....))))....)))))))
low	Gly-GCC	sense	-31	5.8	GCGGGAATAGCTCAGTTGGTAGAGCACGACCTTGCCAAGGTCGGGGTCGCGAGTTCGAGTCTCGTTTCCCGCTCCA	(((((((..((((........)))).(((((((...))))))).....(((((.......))))))))))))....
low	Gly-GCC	antisense	-27.7	9.3	TGGAGCGGGAAACGAGACTCGAACTCGCGACCCCGACCTTGGCAAGGTCGTGCTCTACCAACTGAGCTATTCCCGC	....(((((((.((((.......)))).......(((((((...))))))).((((.......))))..)))))))
none	Val-GAC	sense	-28.6	4.1	AGGCACGTAGCTCAGTTGGTTAGAGCACCACCTTGACATGGTGGGGGTCGTTGGTTCGAATCCAATCGTGCCTACCA	(((((((..((((.........)))).(((((.......)))))((((((......)).))))...)))))))....
none	Val-GAC	antisense	-24.4	7.6	TGGTAGGCACGATTGGATTCGAACCAACGACCCCCACCATGTCAAGGTGGTGCTCTAACCAACTGAGCTACGTGCCT	....(((((((...((..(((......))).))(((((.......))))).((((.........))))..)))))))
none	Pro-CGG	sense	-31.7	6.8	CGGAGCGTAGCGCAGCTTGGTAGCGCGTCTCGTTCGGGACGAGAAGGTCGCTGGTTCGAATCCAGTCGCTCCGACCA	(((((((..((((.(((....)))))))((((((...))))))......(((((.......))))))))))))....
none	Pro-CGG	antisense	-27.7	7	TGGTCGGAGCGACTGGATTCGAACCAGCGACCTTCTCGTCCCGAACGAGACGCGCTACCAAGCTGCGCTACGCTCCG	....(((((((.((((.......)))).......((((((.....)))))).((((.........))))..)))))))
none	Leu-TAG	sense	-34.1	10.3	GCGGACGTGGTGGAATTGGTAGACACACTGGATTTAGGTTCCAGCGCCGCAAGGCGTGAGAGTTCGAGTCTCTCCGTCCGCACCA	(((((((..(((...........))).(((((.......)))))((((....)))).(((((.......))))))))))))....

none	Leu-TAG	antisense	-30.4	5.7	TGGTGCGGACGGAGAGACTCGAACTCTCACGCCTTGCGGCGCTGGAACCTAAATCCAGTGTGTCTACCAATTCCACCACGTCCGC	....((((((((((((.......))))).(((...)))((((((((.......))))))))...............)))))))
none	Ser-CGA	sense	-35.6	10.3	GGAGAGATGCCGGAGTGGTCGAACGGGACGGATTCGAAATCCGTTGAGTCAGCAATGGCTCCTAGGGTTCAAATCCCTATCTCTCCGCCA	((((((((.(((...........)))((((((((...))))))))((((((....)))))).((((.......))))))))))))....
none	Ser-CGA-pseudo	sense	-20.4	19.3	GCCGAAGTGGTAAGAAAATCCCTCGCCGAAAGGCGTGCCGGTTCGATTCCGGCCTCGGGCACCA	(((..((.((.........))..((((....)))).(((((.......)))))))..)))....
none	Ser-CGA	antisense	-27	24.4	TGGCGGAGAGATAGGGATTTGAACCCTAGGAGCCATTGCTGACTCAACGGATTTCGAATCCGTCCCGTTCGACCACTCCGGCATCTCTCC	....((((((((((((.......)))).((((...(((..(((...(((((((...)))))))...))))))...))))...))))))))
none	Ser-CGA-pseudo	antisense	-20	11	TGGTGCCCGAGGCCGGAATCGAACCGGCACGCCTTTCGGCGAGGGATTTTCTTACCACTTCGGC	....(((.((((((((.......))))).((((....)))).((..........)).))).)))
none	Ser-GGA	sense	-36.2	11.5	GGTGAGGTGTCCGAGTGGTTGAAGGAGCACGCCTGGAAAGTGTGTATACGGGAAACCGTATCGAGGGTTCGAATCCCTCCCTCACCGCCA	(((((((..(((...........)))((((((.......))))))((((((....)))))).(((((.......)))))))))))))....
none	Ser-GGA	antisense	-29.9	7.5	TGGCGGTGAGGGAGGGATTCGAACCCTCGATACGGTTTCCCGTATACACACTTTCCAGGCGTGCTCCTTCAACCACTCGGACACCTCACC	....((((((((((((.......))))).((((((....)))))).(((.(((...))).))).(((...........)))..)))))))
none	Leu-GAG	sense	-33.6	17.3	GCCGAGGTGGTGGAATTGGTAGACACGCTACCTTGAGGTGGTAGTGGCCATAGGCTGTAGGGGTTCGAGTCCCCTCCTCGGTACCA	(((((((.((.(((.((((...((((((((((.......))))))))(((...))))))......)))).))))).)))))))....
none	Leu-GAG	antisense	-26.2	6.6	TGGTACCGAGGAGGGGACTCGAACCCCTACAGCCTATGGCCACTACCACCTCAAGGTAGCGTGTCTACCAATTCCACCACCTCGGC	.....(((((((((((.......)))))...(((...)))..(((((.......))))).(((...........)))..)))))).
none	Ser-GCT	sense	-46.3	6.8	GGAGAGGTGGCCGAGTGGCCGAAGGCGCTCCCCTGCTAAGGGAGTACACCTCAAAAGGGTGTCGGGGGTTCGAATCCCCCCCTCTCCGCCA	((((((((((((....)))))).....((((((.......))))))((((((.....)))))).(((((.......)))))))))))))....
none	Ser-GCT	antisense	-44.8	12.7	TGGCGGAGAGGGGGGGATTCGAACCCCCGACACCCTTTTGAGGTGTACTCCCTTAGCAGGGGAGCGCCTTCGGCCACTCGGCCACCTCTCC	....((((((((((((.......)))))...........((((((..(((((((...))))))))))))).((((....)))).)))))))
none	Thr-CGT	sense	-26.7	7.3	GCCGGATTAGCTCAGTCGGTAGAGCAGCTCATTCGTAATGAGAAGGTCGGGGGTTCGATTCCTCTATCCGGCACCA	(((((((..((((........))))..((((((...))))))......(((((......))))).)))))))....
none	Thr-CGT	antisense	-24.1	2.4	TGGTGCCGGATAGAGGAATCGAACCCCCGACCTTCTCATTACGAATGAGCTGCTCTACCGACTGAGCTAATCCGGC	....(((((((.((((..(((......)))))))((((((...))))))..((((........))))..)))))))
none	Gln-TTG	sense	-30.8	8.8	AGGGGCGTCGCCAAGCGGTAAGGCAGCAGGTTTTGATCCTGCCATGCGTTGGTTCGAATCCAGCCGCCCCTGCCA	(((((((((((...))))....((((((((.......)))))..)))(((((.......))))))))))))....
none	Gln-TTG	antisense	-27.8	6.5	TGGCAGGGGCGGCTGGATTCGAACCAACGCATGGCAGGATCAAAACCTGCTGCCTTACCGCTTGGCGACGCCCCT	....((((((((((((.......)))..))..((((((.......))))))(((.........)))..)))))))

[1] Lorenz R, Bernhart SH, Höner zu Siederdissen C, Tafer H, Flamm C, Stadler PF, Hofacker IL. 2011. ViennaRNA Package 2.0. Algorithms Mol Biol 6:26. https://doi.org/10.1186/1748-7188-6-26.

[2] Sato K, Akiyama M, Sakakibara Y. 2021. RNA secondary structure prediction using deep learning with thermodynamic integration. Nat Commun 12:941. https://doi.org/10.1038/s41467-021-21194-4

[3] Zuker M, Stiegler P. 1981. Optimal computer folding of large RNA sequences using thermodynamics and auxiliary information. Nucleic Acids Res 9:133–148. doi: 10.1093/nar/9.1.133.

Supplementary Table S3. Antisense hot spots of the 3% highest expression in NN2 and SG17M in the mid-exponential (4h) and early stationary (8h) growth phase.								
Isolate	Feature	Start	End	Strand	Gene	Length	Time	Biological function (as classified by UniProt)
NN2	CDS	746130	746930	-	ephD_1	800	4h	Aerobic respiration
NN2	CDS	1637122	1638753	-	algG_1	1631	8h	Alginate biosynthesis
SG17M	CDS	5547581	5548408	+	algB_4	827	8h	Alginate biosynthesis, Transcription regulation, Two-component regulatory system
SG17M	CDS	6829938	6832544	+	algC_2	2606	4h	Alginate biosynthesis, Lipopolysaccharide biosynthesis, Virulence
NN2	CDS	3556159	3557535	-	puuA_3	1376	4h	Amine and polyamine degradation
NN2	CDS	6648290	6648910	-	rhtC_4	620	4h	Amino-acid metabolism, Aromatic amino acid metabolism
NN2	CDS	6732729	6733982	+	glyA_3	1253	8h	Amino-acid metabolism, Aromatic amino acid metabolism
NN2	CDS	1195132	1195377	-	amino acid transporter	245	4h	Amino-acid metabolism, Aromatic amino acid metabolism
SG17M	CDS	622633	623325	-	yecS_5	692	4h	Amino-acid metabolism, Aromatic amino acid metabolism
NN2	CDS	6430205	6430978	+	occP_1	773	4h	Amino-acid metabolism, Aromatic amino acid metabolism
SG17M	CDS	622633	623325	-	yecS_5	692	8h	Amino-acid metabolism, Aromatic amino acid metabolism
NN2	CDS	397944	398636	-	yecS_1	692	8h	Amino-acid metabolism, Aromatic amino acid metabolism
NN2	CDS	6499200	6500354	-	argE_1	1154	4h	Amino-acid metabolism, Aromatic amino acid metabolism
SG17M	CDS	604438	605190	+	puuD_2	752	8h	Amino-acid metabolism, Aromatic amino acid metabolism
SG17M	CDS	5436120	5437016	+	nad-dependent L-serine dehydrogenase	896	4h	Amino-acid metabolism, Aromatic amino acid metabolism
SG17M	CDS	963088	963693	+	trpG_2	605	4h	Amino-acid metabolism, Aromatic amino acid metabolism
SG17M	CDS	1941949	1943718	-	asnB_2	1769	4h	Amino-acid metabolism, Aromatic amino acid metabolism
NN2	CDS	2285761	2286855	-	aroF_1	1094	4h	Amino-acid metabolism, Aromatic amino acid metabolism
NN2	CDS	906152	907126	-	acoA_1	974	4h	Amino-acid metabolism, Aromatic amino acid metabolism
SG17M	CDS	942720	944198	+	trpE_2	1478	8h	Amino-acid metabolism, Aromatic amino acid metabolism
SG17M	CDS	6132174	6133346	-	aspC_4	1172	8h	Amino-acid metabolism, Aromatic amino acid metabolism
NN2	CDS	1716775	1718544	-	asnB_1	1769	8h	Amino-acid metabolism, Aromatic amino acid metabolism
NN2	CDS	5905113	5906837	-	ilvI_1	1724	4h	Amino-acid metabolism, Branched-chain amino acid metabolism
SG17M	CDS	6572803	6574482	-	hutU_2	1679	4h	Amino-acid metabolism, Branched-chain amino acid metabolism
SG17M	CDS	3985423	3987081	+	ilvG_2	1658	8h	Amino-acid metabolism, Branched-chain amino acid metabolism

NN2	CDS	3560589	3562247	+	ilvG_1	1658	8h	Amino-acid metabolism, Branched-chain amino acid metabolism
NN2	CDS	6028398	6030839	-	fdoG_1	2441	8h	Anaerobic respiration
SG17M	CDS	474959	475741	-	cloR_3	782	4h	Antibiotic biosynthesis
NN2	CDS	250270	251052	-	cloR_1	782	4h	Antibiotic biosynthesis
NN2	CDS	5605530	5606288	+	tcmP	758	4h	Antibiotic resistance
NN2	CDS	5629072	5629845	+	tcmP	773	4h	Antibiotic resistance
NN2	CDS	4417307	4418755	+	ttgI_5	1448	4h	Antibiotic resistance
SG17M	CDS	6635402	6636880	+	ttgI_12	1478	4h	Antibiotic resistance
SG17M	CDS	4885094	4886542	+	ttgI_11	1448	4h	Antibiotic resistance
NN2	CDS	177587	180634	+	swrC_1	3047	4h	Antibiotic resistance
SG17M	CDS	1520849	1521478	-	sttH_5	629	4h	Antibiotic resistance
NN2	CDS	1296146	1296775	-	sttH_2	629	4h	Antibiotic resistance
NN2	CDS	5684660	5692924	-	ndvB_1	8264	4h	Antibiotic resistance
SG17M	CDS	6510630	6513101	+	mrcA_2	2471	4h	Antibiotic resistance
SG17M	CDS	741770	742213	-	mexR_2	443	4h	Antibiotic resistance
SG17M	CDS	3521429	3536404	+	lgrD_7	14975	4h	Antibiotic resistance
SG17M	CDS	94239	95732	+	cat1_2	1493	4h	Antibiotic resistance
SG17M	CDS	1827500	1828678	+	bcr_4	1178	4h	Antibiotic resistance
SG17M	CDS	6183330	6183860	+	bamE_2	530	4h	Antibiotic resistance
NN2	CDS	5983280	5983810	+	bamE_1	530	4h	Antibiotic resistance
SG17M	CDS	96369	97514	-	wbpZ	1145	8h	Antibiotic resistance
NN2	CDS	5605530	5606288	+	tcmP	758	8h	Antibiotic resistance
NN2	CDS	5629072	5629845	+	tcmP	773	8h	Antibiotic resistance
SG17M	CDS	1063308	1064771	-	ttgI_7	1463	8h	Antibiotic resistance
NN2	CDS	4417307	4418755	+	ttgI_5	1448	8h	Antibiotic resistance
SG17M	CDS	4885094	4886542	+	ttgI_11	1448	8h	Antibiotic resistance
SG17M	CDS	1520849	1521478	-	sttH_5	629	8h	Antibiotic resistance

NN2	CDS	1296146	1296775	-	sttH_2	629	8h	Antibiotic resistance
SG17M	CDS	5898272	5906536	-	ndvB_2	8264	8h	Antibiotic resistance
NN2	CDS	5684660	5692924	-	ndvB_1	8264	8h	Antibiotic resistance
SG17M	CDS	6510630	6513101	+	mrcA_2	2471	8h	Antibiotic resistance
SG17M	CDS	741770	742213	-	mexR_2	443	8h	Antibiotic resistance
SG17M	CDS	3521429	3536404	+	lgrD_7	14975	8h	Antibiotic resistance
SG17M	CDS	2290957	2292516	-	emrB_4	1559	8h	Antibiotic resistance
SG17M	CDS	5354914	5356161	+	cefD_4	1247	8h	Antibiotic resistance
NN2	CDS	4942667	4943914	+	cefD_2	1247	8h	Antibiotic resistance
SG17M	CDS	640424	641149	+	cat_4	725	8h	Antibiotic resistance
SG17M	CDS	1827500	1828678	+	bcr_4	1178	8h	Antibiotic resistance
NN2	CDS	1602558	1603736	+	bcr_2	1178	8h	Antibiotic resistance
SG17M	CDS	6183330	6183860	+	bamE_2	530	8h	Antibiotic resistance
NN2	CDS	5983280	5983810	+	bamE_1	530	8h	Antibiotic resistance
SG17M	CDS	4229390	4230892	+	nhaB_2	1502	8h	Antiport, Ion transport, Sodium transport, Transport
NN2	CDS	3804070	3805572	+	nhaB_1	1502	8h	Antiport, Ion transport, Sodium transport, Transport
NN2	CDS	4858511	4860523	+	neutral ceramidase	2012	8h	Apoptosis, Lipid metabolism, Sphingolipid metabolism
NN2	CDS	5483524	5485017	+	rpoN_1	1493	8h	Arginine metabolism, Transcription, Transcription regulation
NN2	CDS	6852140	6852898	-	tsaC1_1	758	8h	Aromatic hydrocarbons catabolism
NN2	CDS	935369	936673	-	nicT_1	1304	8h	Aromatic hydrocarbons catabolism
NN2	CDS	933729	934535	-	hpcH_1	806	8h	Aromatic hydrocarbons catabolism
NN2	CDS	934548	935351	-	hpcG_1	803	8h	Aromatic hydrocarbons catabolism
NN2	CDS	6133799	6134752	+	pcpD_2	953	4h	Aromatic hydrocarbons catabolism, Electron transport, Transport
SG17M	CDS	596990	598372	+	nicP_13	1382	4h	Aromatic hydrocarbons catabolism, Transport
NN2	CDS	372301	373683	+	nicP_2	1382	8h	Aromatic hydrocarbons catabolism, Transport
NN2	CDS	4198755	4199588	-	ylxH_1	833	8h	Bacterial flagellum biogenesis
SG17M	CDS	6007007	6007582	-	pucB_2	575	4h	Bacteriochlorophyll, Chlorophyll, Chromophore, Magnesium, Metal-binding

SG17M	CDS	4164735	4172879	-	bapA	8144	4h	Biofilm formation
NN2	CDS	3739673	3747559	-	bapA	7886	4h	Biofilm formation
SG17M	CDS	178446	179126	+	mcbR_2	680	4h	Biofilm formation
NN2	CDS	6856719	6857399	+	mcbR_1	680	4h	Biofilm formation
SG17M	CDS	4164735	4172879	-	bapA	8144	8h	Biofilm formation
NN2	CDS	3739673	3747559	-	bapA	7886	8h	Biofilm formation
SG17M	CDS	178446	179126	+	mcbR_2	680	8h	Biofilm formation
NN2	CDS	4348918	4349889	+	czcD_2	971	8h	Cadmium resistance, Ion transport, Transport, Zinc transport
SG17M	CDS	4509459	4510688	-	czcB_4	1229	4h	Cadmium resistance, Transport
NN2	CDS	4907798	4909027	+	czcB_2	1229	4h	Cadmium resistance, Transport
NN2	CDS	4909038	4912178	+	czcA_2	3140	4h	Cadmium resistance, Transport
NN2	CDS	4907798	4909027	+	czcB_2	1229	8h	Cadmium resistance, Transport
SG17M	CDS	6775127	6777979	+	cyaA_2	2852	4h	cAMP biosynthesis
NN2	CDS	6575079	6577931	+	cyaA_1	2852	8h	cAMP biosynthesis
NN2	CDS	3277270	3278220	-	kdgK_1	950	8h	Carbohydrate metabolism
SG17M	CDS	4139632	4140345	-	anoR_2	713	8h	Carbohydrate metabolism
SG17M	CDS	977246	978337	-	anmK_2	1091	8h	Carbohydrate metabolism
NN2	CDS	5876585	5877232	-	can_1	647	4h	Carbon utilisation
NN2	CDS	1542106	1543218	+	frmA_1	1112	4h	Cell adhesion
NN2	CDS	1296896	1297849	+	cdhR_3	953	4h	Cell adhesion
SG17M	CDS	1521599	1522552	+	cdhR_12	953	4h	Cell adhesion
SG17M	CDS	4073721	4074986	+	fap	1265	8h	Cell adhesion
NN2	CDS	1542106	1543218	+	frmA_1	1112	8h	Cell adhesion
SG17M	CDS	1744357	1746744	+	bamA_2	2387	8h	Cell adhesion
SG17M	CDS	44533	44823	+	zapA	290	4h	Cell cycle, Cell division, DNA damage, Septation, SOS response
SG17M	CDS	5617446	5618504	+	sacchrp dh NADP domain-containing protein	1058	4h	Cell cycle, Cell division, DNA damage, Septation, SOS response
SG17M	CDS	5884842	5887784	+	ftsK domain-containing protein	2942	4h	Cell cycle, Cell division, DNA damage, Septation, SOS response

SG17M	CDS	5806529	5807587	-	zapE_6	1058	4h	Cell cycle, Cell division, DNA damage, Septation, SOS response
NN2	CDS	5610020	5610979	+	xerD_3	959	4h	Cell cycle, Cell division, DNA damage, Septation, SOS response
SG17M	CDS	2442508	2442993	-	sulA_3	485	4h	Cell cycle, Cell division, DNA damage, Septation, SOS response
NN2	CDS	2217328	2217813	-	sulA_1	485	4h	Cell cycle, Cell division, DNA damage, Septation, SOS response
NN2	CDS	6309332	6310396	-	ftsZ	1184	4h	Cell cycle, Cell division, DNA damage, Septation, SOS response
SG17M	CDS	44533	44823	+	zapA	290	8h	Cell cycle, Cell division, DNA damage, Septation, SOS response
NN2	CDS	4935885	4936586	+	trbL	701	8h	Cell cycle, Cell division, DNA damage, Septation, SOS response
SG17M	CDS	5548434	5550104	-	tadC domain-containing protein	1670	8h	Cell cycle, Cell division, DNA damage, Septation, SOS response
SG17M	CDS	6302949	6303356	-	rho N domain-containing protein	407	8h	Cell cycle, Cell division, DNA damage, Septation, SOS response
SG17M	CDS	5884842	5887784	+	ftsK domain-containing protein	2942	8h	Cell cycle, Cell division, DNA damage, Septation, SOS response
SG17M	CDS	5352452	5353336	+	yofA_10	884	8h	Cell cycle, Cell division, DNA damage, Septation, SOS response
NN2	CDS	5610020	5610979	+	xerD_3	959	8h	Cell cycle, Cell division, DNA damage, Septation, SOS response
NN2	CDS	5406133	5407776	-	groL_1	1643	8h	Cell cycle, Cell division, DNA damage, Septation, SOS response
SG17M	CDS	6502098	6503753	-	damX_2	1655	8h	Cell cycle, Cell division, DNA damage, Septation, SOS response
NN2	CDS	1828598	1829335	-	flgA	737	4h	Cell motility
SG17M	CDS	2053774	2054511	-	flgA	737	8h	Cell motility
SG17M	CDS	6869119	6870018	-	mepM_8	899	4h	Cell shape, Cell wall biogenesis/degradation, Peptidoglycan synthesis
SG17M	CDS	978340	979779	-	mepM_5	1439	4h	Cell shape, Cell wall biogenesis/degradation, Peptidoglycan synthesis
NN2	CDS	6669069	6669968	-	mepM_4	899	4h	Cell shape, Cell wall biogenesis/degradation, Peptidoglycan synthesis
NN2	CDS	753639	755078	-	mepM_1	1439	4h	Cell shape, Cell wall biogenesis/degradation, Peptidoglycan synthesis
NN2	CDS	3264528	3266225	-	pbpC_1	1697	8h	Cell shape, Cell wall biogenesis/degradation, Peptidoglycan synthesis
NN2	CDS	4553317	4553823	+	oprF_3	506	8h	Cell shape, Cell wall biogenesis/degradation, Peptidoglycan synthesis
SG17M	CDS	978340	979779	-	mepM_5	1439	8h	Cell shape, Cell wall biogenesis/degradation, Peptidoglycan synthesis
NN2	CDS	753639	755078	-	mepM_1	1439	8h	Cell shape, Cell wall biogenesis/degradation, Peptidoglycan synthesis
NN2	CDS	645571	646314	-	cpdA_1	743	8h	Cell shape, Cell wall biogenesis/degradation, Peptidoglycan synthesis

SG17M	CDS	6362761	6363837	-	alr_2	1076	8h	Cell shape, Cell wall biogenesis/degradation, Peptidoglycan synthesis
SG17M	CDS	451172	452629	-	aam_4	1457	4h	Cellular amide catabolic process
SG17M	CDS	451172	452629	-	aam_4	1457	8h	Cellular amide catabolic process
SG17M	CDS	5760354	5761952	+	dppA_7	1598	4h	Chemotaxis, two-component regulatory system, signal transduction
NN2	CDS	2978178	2979500	-	qseC_2	1322	4h	Chemotaxis, two-component regulatory system, signal transduction
NN2	CDS	4398491	4400116	+	mcpQ_6	1625	4h	Chemotaxis, two-component regulatory system, signal transduction
SG17M	CDS	724590	732008	+	cheA_6	7418	4h	Chemotaxis, two-component regulatory system, signal transduction
NN2	CDS	499890	507308	+	cheA_2	7418	4h	Chemotaxis, two-component regulatory system, signal transduction
SG17M	CDS	5179466	5180140	-	queC_2	674	8h	Chemotaxis, two-component regulatory system, signal transduction
NN2	CDS	4398491	4400116	+	mcpQ_6	1625	8h	Chemotaxis, two-component regulatory system, signal transduction
SG17M	CDS	4866278	4867903	+	mcpQ_14	1625	8h	Chemotaxis, two-component regulatory system, signal transduction
SG17M	CDS	6344464	6346089	+	mcpH_4	1625	8h	Chemotaxis, two-component regulatory system, signal transduction
NN2	CDS	2879599	2881215	-	ctpH_1	1616	8h	Chemotaxis, two-component regulatory system, signal transduction
NN2	CDS	5301933	5303138	+	srpC_1	1205	4h	Chromate resistance, Stress response, Transport
NN2	CDS	5577747	5578607	+	soj_7	860	8h	Chromosome partition
SG17M	CDS	6505446	6507590	-	pilQ_2	2144	4h	Competence, Protein transport, Transport
NN2	CDS	5681619	5683154	+	traG	1535	4h	Conjugation
SG17M	CDS	5895231	5896766	+	traG	1535	4h	Conjugation
NN2	CDS	5681619	5683154	+	traG	1535	8h	Conjugation
SG17M	CDS	5895231	5896766	+	traG	1535	8h	Conjugation
SG17M	CDS	5172807	5174318	+	traG	1511	8h	Conjugation
NN2	CDS	5618409	5620934	-	virD4	2525	8h	Conjugation
SG17M	CDS	4711629	4712357	+	gloC_3	728	4h	Detoxification
NN2	CDS	4243844	4244572	+	gloC_1	728	4h	Detoxification
SG17M	CDS	4711629	4712357	+	gloC_3	728	8h	Detoxification
SG17M	CDS	227864	228973	+	recF_2	1109	4h	DNA damage, DNA repair, DNA replication
SG17M	CDS	5512196	5515270	+	dnaE2_2	3074	4h	DNA damage, DNA repair, DNA replication

NN2	CDS	5275685	5278759	+	dnaE2_1	3074	4h	DNA damage, DNA repair, DNA replication
NN2	CDS	4773302	4774351	-	dinB_1	1049	4h	DNA damage, DNA repair, DNA replication
SG17M	CDS	5998185	5999546	-	radA_2	1361	8h	DNA damage, DNA repair, DNA replication
SG17M	CDS	5512196	5515270	+	dnaE2_2	3074	8h	DNA damage, DNA repair, DNA replication
SG17M	CDS	1280612	1281205	+	lon	593	4h	DNA damage, DNA repair, Stress response
NN2	CDS	1055909	1056502	+	lon	593	4h	DNA damage, DNA repair, Stress response
NN2	CDS	1427989	1433229	+	dna repair ATPase	5240	4h	DNA damage, DNA repair, Stress response
SG17M	CDS	1652692	1657932	+	dna repair ATPase	5240	4h	DNA damage, DNA repair, Stress response
NN2	CDS	5819926	5820540	-	abasic site-processing protein	614	4h	DNA damage, DNA repair, Stress response
SG17M	CDS	144179	146920	-	polA_2	2741	4h	DNA damage, DNA repair, Stress response
SG17M	CDS	1774714	1777281	-	mutS_2	2567	4h	DNA damage, DNA repair, Stress response
NN2	CDS	1550001	1552568	-	mutS_1	2567	4h	DNA damage, DNA repair, Stress response
SG17M	CDS	2148554	2152900	+	cshB_2	4346	4h	DNA damage, DNA repair, Stress response
NN2	CDS	1923383	1927729	+	cshB_1	4346	4h	DNA damage, DNA repair, Stress response
NN2	CDS	5662794	5664275	+	addA_1	1481	4h	DNA damage, DNA repair, Stress response
SG17M	CDS	1280612	1281205	+	lon	593	8h	DNA damage, DNA repair, Stress response
NN2	CDS	1055909	1056502	+	lon	593	8h	DNA damage, DNA repair, Stress response
NN2	CDS	1427989	1433229	+	dna repair ATPase	5240	8h	DNA damage, DNA repair, Stress response
SG17M	CDS	6033776	6034390	-	abasic site-processing protein	614	8h	DNA damage, DNA repair, Stress response
NN2	CDS	6822452	6825193	-	polA_1	2741	8h	DNA damage, DNA repair, Stress response
NN2	CDS	2610102	2612624	-	helD_1	2522	8h	DNA damage, DNA repair, Stress response
SG17M	CDS	2148554	2152900	+	cshB_2	4346	8h	DNA damage, DNA repair, Stress response
NN2	CDS	1923383	1927729	+	cshB_1	4346	8h	DNA damage, DNA repair, Stress response
NN2	CDS	3947816	3948709	+	alkA_1	893	8h	DNA damage, DNA repair, Stress response
NN2	CDS	5662794	5664275	+	addA_1	1481	8h	DNA damage, DNA repair, Stress response
NN2	CDS	324199	327330	+	tyr_recombinase_domain-containing_protein	3131	4h	DNA integration/DNA recombination
SG17M	CDS	552623	553783	+	intQ_2	1160	4h	DNA integration/DNA recombination

NN2	CDS	327934	329094	+	intQ_1	1160	4h	DNA integration/DNA recombination
SG17M	CDS	2863284	2864666	-	intA_3	1382	4h	DNA integration/DNA recombination
NN2	CDS	324199	327330	+	tyr_recombinase_domain-containing_protein	3131	8h	DNA integration/DNA recombination
SG17M	CDS	552623	553783	+	intQ_2	1160	8h	DNA integration/DNA recombination
NN2	CDS	327934	329094	+	intQ_1	1160	8h	DNA integration/DNA recombination
SG17M	CDS	5522688	5526323	+	sbcC_2	3635	4h	DNA recombination, Transposition
NN2	CDS	5611096	5611632	-	tnpA	536	8h	DNA recombination, Transposition
NN2	CDS	995675	996343	-	transcriptional_regulator	668	4h	DNA replication, Transcription, Transcription regulation
NN2	CDS	308773	314988	-	dead/deah box helicase	6215	4h	DNA replication, Transcription, Transcription regulation
SG17M	CDS	533462	539677	-	dead/deah box helicase	6215	4h	DNA replication, Transcription, Transcription regulation
NN2	CDS	457009	457656	+	acrR	647	4h	DNA replication, Transcription, Transcription regulation
NN2	CDS	583962	584912	-	yhaJ_1	950	4h	DNA replication, Transcription, Transcription regulation
NN2	CDS	6601401	6603410	+	rep_1	2009	4h	DNA replication, Transcription, Transcription regulation
NN2	CDS	233172	234836	+	madA_1	1664	4h	DNA replication, Transcription, Transcription regulation
NN2	CDS	2016425	2017282	-	hexR_1	857	4h	DNA replication, Transcription, Transcription regulation
NN2	CDS	683333	685327	-	dnaG_1	1994	4h	DNA replication, Transcription, Transcription regulation
SG17M	CDS	6363906	6365300	-	dnaB_4	1394	4h	DNA replication, Transcription, Transcription regulation
SG17M	CDS	5819539	5820882	+	dnaB_3	1343	4h	DNA replication, Transcription, Transcription regulation
NN2	CDS	5582867	5584210	+	dnaB_1	1343	4h	DNA replication, Transcription, Transcription regulation
SG17M	CDS	225178	226722	+	dnaA_2	1544	4h	DNA replication, Transcription, Transcription regulation
NN2	CDS	483	2027	+	dnaA_1	1544	4h	DNA replication, Transcription, Transcription regulation
NN2	CDS	631190	632068	-	cynR_3	878	4h	DNA replication, Transcription, Transcription regulation
NN2	CDS	308773	314988	-	dead/deah box helicase	6215	8h	DNA replication, Transcription, Transcription regulation
SG17M	CDS	533462	539677	-	dead/deah box helicase	6215	8h	DNA replication, Transcription, Transcription regulation
NN2	CDS	457009	457656	+	acrR	647	8h	DNA replication, Transcription, Transcription regulation
SG17M	CDS	1700389	1700859	+	zntR_3	470	8h	DNA replication, Transcription, Transcription regulation
NN2	CDS	1475678	1476148	+	zntR_1	470	8h	DNA replication, Transcription, Transcription regulation

NN2	CDS	1230671	1231675	-	yejK_1	1004	8h	DNA replication, Transcription, Transcription regulation
NN2	CDS	430522	431316	+	thyA_1	794	8h	DNA replication, Transcription, Transcription regulation
SG17M	CDS	2152908	2156387	-	pleC_2	3479	8h	DNA replication, Transcription, Transcription regulation
NN2	CDS	1927737	1931216	-	pleC_1	3479	8h	DNA replication, Transcription, Transcription regulation
NN2	CDS	3129420	3129899	+	fecI_6	479	8h	DNA replication, Transcription, Transcription regulation
NN2	CDS	925694	926116	+	farR_1	422	8h	DNA replication, Transcription, Transcription regulation
NN2	CDS	5582867	5584210	+	dnaB_1	1343	8h	DNA replication, Transcription, Transcription regulation
NN2	CDS	483	2027	+	dnaA_1	1544	8h	DNA replication, Transcription, Transcription regulation
SG17M	CDS	4923222	4924136	+	dmlR_49	914	8h	DNA replication, Transcription, Transcription regulation
SG17M	CDS	365149	366057	-	dmlR_28	908	8h	DNA replication, Transcription, Transcription regulation
NN2	CDS	140460	141368	-	dmlR_2	908	8h	DNA replication, Transcription, Transcription regulation
SG17M	CDS	1440701	1441333	-	dauR_2	632	8h	DNA replication, Transcription, Transcription regulation
NN2	CDS	1215998	1216630	-	dauR_1	632	8h	DNA replication, Transcription, Transcription regulation
SG17M	CDS	855891	856769	-	cynR_9	878	8h	DNA replication, Transcription, Transcription regulation
SG17M	CDS	5834749	5836668	+	topA_4	1919	8h	DNA topological change
NN2	CDS	5598077	5599996	+	topA_2	1919	8h	DNA topological change
SG17M	CDS	4510685	4511932	-	czcC_4	1247	4h	Efflux transmembrane transporter activity
NN2	CDS	4906554	4907801	+	czcC_2	1247	8h	Efflux transmembrane transporter activity
NN2	CDS	2271900	2273555	-	flavoprotein-ubiquinone_oxidoreductase	1655	4h	Electron transport, Respiratory chain, Transport
SG17M	CDS	2227777	2227989	+	fe-S protein	212	4h	Electron transport, Respiratory chain, Transport
SG17M	CDS	1728385	1728738	+	fe-S oxidoreductase	353	4h	Electron transport, Respiratory chain, Transport
SG17M	CDS	6189589	6192405	+	fad binding oxidoreductase	2816	4h	Electron transport, Respiratory chain, Transport
SG17M	CDS	2497080	2498735	-	electron transfer-flavoprotein-ubiquinone oxidoreductase	1655	4h	Electron transport, Respiratory chain, Transport
SG17M	CDS	142791	143084	-	cytochrome C domain-containing protein	293	4h	Electron transport, Respiratory chain, Transport
SG17M	CDS	3228386	3229576	+	thlA_4	1190	4h	Electron transport, Respiratory chain, Transport
SG17M	CDS	57455	60472	+	soxA_2	3017	4h	Electron transport, Respiratory chain, Transport
SG17M	CDS	624955	626184	-	serA_2	1229	4h	Electron transport, Respiratory chain, Transport

SG17M	CDS	444730	445848	+	pntAA_2	1118	4h	Electron transport, Respiratory chain, Transport
SG17M	CDS	1053929	1055137	-	phzS_2	1208	4h	Electron transport, Respiratory chain, Transport
SG17M	CDS	1430559	1434344	+	narG_2	3785	4h	Electron transport, Respiratory chain, Transport
NN2	CDS	2271900	2273555	-	flavoprotein-ubiquinone_oxidoreductase	1655	8h	Electron transport, Respiratory chain, Transport
SG17M	CDS	1728385	1728738	+	fe-S oxidoreductase	353	8h	Electron transport, Respiratory chain, Transport
SG17M	CDS	1333905	1335044	-	endo/exonuclease/phosphatase domain-containing protein	1139	8h	Electron transport, Respiratory chain, Transport
SG17M	CDS	2497080	2498735	-	electron transfer-flavoprotein-ubiquinone oxidoreductase	1655	8h	Electron transport, Respiratory chain, Transport
NN2	CDS	2437956	2438597	-	cytochrome C domain-containing protein	641	8h	Electron transport, Respiratory chain, Transport
SG17M	CDS	3228386	3229576	+	thlA_4	1190	8h	Electron transport, Respiratory chain, Transport
NN2	CDS	6735729	6738746	+	soxA_1	3017	8h	Electron transport, Respiratory chain, Transport
SG17M	CDS	624955	626184	-	serA_2	1229	8h	Electron transport, Respiratory chain, Transport
SG17M	CDS	1053929	1055137	-	phzS_2	1208	8h	Electron transport, Respiratory chain, Transport
NN2	CDS	2748740	2749153	-	ndhC_1	413	8h	Electron transport, Respiratory chain, Transport
NN2	CDS	4093487	4094443	+	ccoP2_1	956	8h	Electron transport, Respiratory chain, Transport
SG17M	CDS	3687342	3688403	+	adhA_2	1061	8h	Electron transport, Respiratory chain, Transport
NN2	CDS	3262509	3263570	+	adhA_1	1061	8h	Electron transport, Respiratory chain, Transport
SG17M	CDS	1932052	1933767	-	entS_4	1715	4h	Enterobactin biosynthesis, enterobactin transport
NN2	CDS	4503236	4503964	+	entD_1	728	4h	Enterobactin biosynthesis, enterobactin transport
SG17M	CDS	5798883	5800103	+	epsF_8	1220	4h	Exopolysaccharide synthesis
NN2	CDS	5562211	5563431	+	epsF_4	1220	4h	Exopolysaccharide synthesis
SG17M	CDS	6704009	6705793	+	epsE_8	1784	4h	Exopolysaccharide synthesis
SG17M	CDS	5798883	5800103	+	epsF_8	1220	8h	Exopolysaccharide synthesis
NN2	CDS	5562211	5563431	+	epsF_4	1220	8h	Exopolysaccharide synthesis
SG17M	CDS	6704009	6705793	+	epsE_8	1784	8h	Exopolysaccharide synthesis
NN2	CDS	2337148	2338974	-	long chain acyl-CoA synthetase	1826	4h	Fatty acid metabolism, Lipid degradation, Lipid metabolism
NN2	CDS	5231046	5231951	+	Fatty acid hydroxylase domain-containing protein	905	4h	Fatty acid metabolism, Lipid degradation, Lipid metabolism

NN2	CDS	2377059	2377664	+	tesA_1	605	4h	Fatty acid metabolism, Lipid degradation, Lipid metabolism
NN2	CDS	6716762	6718723	+	fadF_1	1961	4h	Fatty acid metabolism, Lipid degradation, Lipid metabolism
SG17M	CDS	2434915	2437062	+	fadB_5	2147	4h	Fatty acid metabolism, Lipid degradation, Lipid metabolism
NN2	CDS	2209735	2211882	+	fadB_1	2147	4h	Fatty acid metabolism, Lipid degradation, Lipid metabolism
SG17M	CDS	6205371	6206726	+	fabG_30	1355	4h	Fatty acid metabolism, Lipid degradation, Lipid metabolism
NN2	CDS	5409695	5410453	+	fabG_12	758	4h	Fatty acid metabolism, Lipid degradation, Lipid metabolism
SG17M	CDS	2562327	2564153	-	long chain acyl-CoA synthetase	1826	8h	Fatty acid metabolism, Lipid degradation, Lipid metabolism
SG17M	CDS	2434915	2437062	+	fadB_5	2147	8h	Fatty acid metabolism, Lipid degradation, Lipid metabolism
NN2	CDS	2209735	2211882	+	fadB_1	2147	8h	Fatty acid metabolism, Lipid degradation, Lipid metabolism
SG17M	CDS	6205371	6206726	+	fabG_30	1355	8h	Fatty acid metabolism, Lipid degradation, Lipid metabolism
NN2	CDS	6005316	6006671	+	fabG_13	1355	8h	Fatty acid metabolism, Lipid degradation, Lipid metabolism
NN2	CDS	6476648	6477811	-	adh1_1	1163	4h	Fermentation
NN2	CDS	6476648	6477811	-	adh1_1	1163	8h	Fermentation
NN2	CDS	5631143	5632852	+	bfpB_1	1709	8h	Fimbrium biogenesis
SG17M	CDS	707034	708068	+	pilT_4	1034	4h	Fimbrium biogenesis, Transport
NN2	CDS	4582371	4583555	-	fliC_1	1184	4h	Flagellar synthesis
SG17M	CDS	2051961	2052710	-	ycgR_2	749	8h	Flagellum-dependent motility
NN2	CDS	5608004	5608843	-	folP_1	839	8h	Folate biosynthesis
SG17M	CDS	1281216	1282529	-	yabI_2	1313	4h	FtsZ-dependent cytokinesis
NN2	CDS	1056513	1057826	-	yabI_1	1313	4h	FtsZ-dependent cytokinesis
NN2	CDS	2225593	2227059	+	gap2_1	1466	8h	Glucose metabolic process
SG17M	CDS	718895	719848	-	gshB_2	953	4h	Glutathione biosynthesis
NN2	CDS	494195	495148	-	gshB_1	953	4h	Glutathione biosynthesis
NN2	CDS	6495265	6496848	+	gshA_1	1583	4h	Glutathione biosynthesis
SG17M	CDS	718895	719848	-	gshB_2	953	8h	Glutathione biosynthesis
SG17M	CDS	4655613	4656299	+	gstB_6	686	4h	Glutathione metabolic process
NN2	CDS	4187829	4188515	+	gstB_3	686	4h	Glutathione metabolic process

SG17M	CDS	4655613	4656299	+	gstB_6	686	8h	Glutathione metabolic process
SG17M	CDS	2462713	2463435	-	ugpQ_2	722	4h	Glycerol metabolism
SG17M	CDS	2462713	2463435	-	ugpQ_2	722	8h	Glycerol metabolism
SG17M	CDS	6731691	6733037	+	glpT_4	1346	4h	Glycerol metabolism, Transport
NN2	CDS	6506914	6509790	-	gcvP_2	2876	4h	glycine decarboxylation
SG17M	CDS	6706963	6709839	-	gcvP_4	2876	8h	glycine decarboxylation
NN2	CDS	6506914	6509790	-	gcvP_2	2876	8h	glycine decarboxylation
NN2	CDS	3291032	3292318	-	bkdB_1	1286	8h	Glycolysis
NN2	CDS	6265838	6268486	+	aceE_1	2648	8h	Glycolysis
SG17M	CDS	4621127	4621909	+	hyi_2	782	4h	Glyoxylate metabolic process
SG17M	CDS	6017313	6024023	-	hxuA_8	6710	4h	Heme/hemopexin-binding
NN2	CDS	984588	987644	+	hxuA_1	3056	4h	Heme/hemopexin-binding
SG17M	CDS	1209286	1212342	+	hxuA_5	3056	8h	Heme/hemopexin-binding
NN2	CDS	5803700	5810173	-	hxuA_4	6473	8h	Heme/hemopexin-binding
NN2	CDS	984588	987644	+	hxuA_1	3056	8h	Heme/hemopexin-binding
SG17M	CDS	1257252	1258253	-	fecR domain-containing protein	1001	4h	Ion transport, Iron transport, Sensory transduction, Transport
SG17M	CDS	3914557	3917208	-	pfeA_5	2651	4h	Ion transport, Iron transport, Transport
SG17M	CDS	2911621	2913861	-	pfeA_4	2240	4h	Ion transport, Iron transport, Transport
NN2	CDS	3489723	3492374	-	pfeA_2	2651	4h	Ion transport, Iron transport, Transport
SG17M	CDS	3940844	3943486	+	cirA_7	2642	4h	Ion transport, Iron transport, Transport
SG17M	CDS	3914557	3917208	-	pfeA_5	2651	8h	Ion transport, Iron transport, Transport
NN2	CDS	3489723	3492374	-	pfeA_2	2651	8h	Ion transport, Iron transport, Transport
SG17M	CDS	2540695	2541726	-	hmuU_3	1031	8h	Ion transport, Iron transport, Transport
SG17M	CDS	3940844	3943486	+	cirA_7	2642	8h	Ion transport, Iron transport, Transport
NN2	CDS	4025438	4027546	+	cirA_5	2108	8h	Ion transport, Iron transport, Transport
SG17M	CDS	5359827	5361143	+	mntH_3	1316	8h	Ion transport, Symport, Transport
NN2	CDS	6831241	6832050	+	znuC_2	809	4h	Ion transport, Transport, Zinc transport

SG17M	CDS	798898	801312	-	fhuA_4	2414	4h	Iron transport
NN2	CDS	574199	576613	-	fhuA_1	2414	4h	Iron transport
SG17M	CDS	798898	801312	-	fhuA_4	2414	8h	Iron transport
SG17M	CDS	489916	490731	+	iolI_2	815	4h	Isomerase activity
SG17M	exon	1769180	1769274	-	ispF	94	4h	Isoprene biosynthesis
NN2	CDS	5722271	5723215	-	ispH_1	944	4h	Isoprene biosynthesis
SG17M	CDS	5606703	5607479	+	olsA	776	4h	Lipid biosynthesis, Lipid metabolism, Phospholipid biosynthesis, Phospholipid metabolism
SG17M	CDS	5717446	5717985	+	kdsC_2	539	8h	Lipopolysaccharide biosynthesis
NN2	CDS	5480939	5481478	+	kdsC_1	539	8h	Lipopolysaccharide biosynthesis
SG17M	CDS	5717994	5718566	+	lptC_2	572	8h	Lipopolysaccharide transport
NN2	CDS	5481487	5482059	+	lptC_1	572	8h	Lipopolysaccharide transport
NN2	CDS	2432198	2433733	-	lnt_2	1535	4h	Lipoprotein biosynthesis
NN2	CDS	2432198	2433733	-	lnt_2	1535	8h	Lipoprotein biosynthesis
SG17M	CDS	3082584	3083297	+	merR	713	4h	Mercuric resistance, Transcription, Transcription regulation
SG17M	CDS	3082584	3083297	+	merR	713	8h	Mercuric resistance, Transcription, Transcription regulation
SG17M	CDS	4754659	4755057	+	phnB	398	4h	Methylation
NN2	CDS	5677502	5678368	+	tam_5	866	4h	Methylation
SG17M	CDS	768879	770162	+	hyuC_2	1283	8h	Nitrogen compound metabolism
SG17M	CDS	4411392	4412285	-	panS_4	893	4h	Pantothenate precursors transporter
NN2	CDS	6325052	6326731	+	phaC_1	1679	8h	PHB biosynthesis
NN2	CDS	687449	688018	-	plsY_1	569	4h	Phospholipid biosynthetic process
SG17M	CDS	2913960	2915300	-	pfeS_5	1340	4h	Phosphorelay signal transduction system
SG17M	CDS	2913960	2915300	-	pfeS_5	1340	8h	Phosphorelay signal transduction system
NN2	CDS	2690428	2691768	-	pfeS_2	1340	8h	Phosphorelay signal transduction system
SG17M	CDS	1843050	1844807	+	fruA_2	1757	8h	Phosphotransferase system, Sugar transport, Transport
SG17M	CDS	5174807	5175079	+	parD	272	4h	Plasmid partition, Toxin-antitoxin system, Transcription, Transcription regulation

NN2	CDS	4717348	4717620	+	parD	272	8h	Plasmid partition, Toxin-antitoxin system, Transcription, Transcription regulation
SG17M	CDS	6193316	6194443	+	speE_6	1127	8h	Polyamine biosynthesis, Spermidine biosynthesis
SG17M	CDS	6739674	6741179	+	ppx_2	1505	4h	Polyphosphate catabolism
NN2	CDS	6539626	6541131	+	ppx_1	1505	4h	Polyphosphate catabolism
SG17M	CDS	6739674	6741179	+	ppx_2	1505	8h	Polyphosphate catabolism
NN2	CDS	6539626	6541131	+	ppx_1	1505	8h	Polyphosphate catabolism
NN2	CDS	4100051	4101433	+	hemN_1	1382	4h	Porphyrin biosynthesis
SG17M	CDS	2639666	2640727	-	hemE_2	1061	4h	Porphyrin biosynthesis
SG17M	CDS	6741166	6743376	-	ppk_2	2210	4h	Protein autophosphorylation
NN2	CDS	6541118	6543328	-	ppk_1	2210	4h	Protein autophosphorylation
SG17M	CDS	1469679	1472531	-	valS_2	2852	4h	Protein biosynthesis
SG17M	CDS	1028300	1028797	+	ssb_2	497	8h	Protein biosynthesis
NN2	CDS	2750386	2752404	-	putative phosphatase	2018	8h	Protein dephosphorylation
NN2	CDS	3870151	3870969	+	ppsR_1	818	8h	Protein dephosphorylation
SG17M	CDS	4201789	4204077	-	cis/trans isomerase	2288	4h	Protein folding
SG17M	CDS	4201789	4204077	-	cis/trans isomerase	2288	8h	Protein folding
NN2	CDS	877387	877668	+	ppiC_1	281	8h	Protein folding
NN2	CDS	2101996	2102652	-	xcpP_1	656	4h	Protein secretion
NN2	CDS	4659416	4660702	-	puuB_10	1286	4h	Putrescine catabolism
SG17M	CDS	5416397	5418013	+	nadB_2	1616	4h	Pyridine nucleotide biosynthesis
NN2	CDS	5004148	5005764	+	nadB_1	1616	4h	Pyridine nucleotide biosynthesis
SG17M	CDS	5416397	5418013	+	nadB_2	1616	8h	Pyridine nucleotide biosynthesis
NN2	CDS	2002825	2003829	-	epd_2	1004	8h	Pyridoxine biosynthesis
SG17M	CDS	2395795	2396823	+	pyrD_2	1028	8h	Pyrimidine biosynthesis
SG17M	CDS	193414	194751	+	pyrC_3	1337	8h	Pyrimidine biosynthesis
SG17M	CDS	3071032	3072573	-	deoA	1541	4h	Pyrimidine metabolism
NN2	CDS	4774753	4775067	+	transcriptional_regulator	314	4h	Quorum sensing, Transcription, Transcription regulation

SG17M	CDS	5232212	5232526	+	transcriptional_regulator	314	4h	Quorum sensing, Transcription, Transcription regulation
SG17M	CDS	6753823	6754857	-	transcriptional_regulator	1034	4h	Quorum sensing, Transcription, Transcription regulation
SG17M	CDS	5110717	5113260	+	quiP_6	2543	4h	Quorum sensing, Transcription, Transcription regulation
NN2	CDS	4653258	4655801	+	quiP_3	2543	4h	Quorum sensing, Transcription, Transcription regulation
NN2	CDS	5985388	5986161	-	pdhR_1	773	4h	Quorum sensing, Transcription, Transcription regulation
SG17M	CDS	3230849	3231781	-	pcpR_12	932	4h	Quorum sensing, Transcription, Transcription regulation
NN2	CDS	4224670	4225389	-	lasR_1	719	4h	Quorum sensing, Transcription, Transcription regulation
SG17M	CDS	3043271	3044161	-	hcaR_9	890	4h	Quorum sensing, Transcription, Transcription regulation
NN2	CDS	5193265	5194173	-	hcaR_4	908	4h	Quorum sensing, Transcription, Transcription regulation
SG17M	CDS	3418799	3419707	+	hcaR_11	908	4h	Quorum sensing, Transcription, Transcription regulation
NN2	CDS	4224670	4225389	-	lasR_1	719	8h	Quorum sensing, Transcription, Transcription regulation
NN2	CDS	5193265	5194173	-	hcaR_4	908	8h	Quorum sensing, Transcription, Transcription regulation
SG17M	CDS	3418799	3419707	+	hcaR_11	908	8h	Quorum sensing, Transcription, Transcription regulation
SG17M	CDS	5290082	5290495	-	arfB_2	413	4h	Rescue of stalled ribosome
NN2	CDS	4832624	4833037	-	arfB_1	413	4h	Rescue of stalled ribosome
SG17M	CDS	5290082	5290495	-	arfB_2	413	8h	Rescue of stalled ribosome
NN2	CDS	4634395	4634637	+	ribosomal protein 30S-S3	242	4h	Ribonucleoprotein, Ribosomal protein, RNA-binding, rRNA-binding
SG17M	CDS	6386678	6387697	+	rsgA_2	1019	4h	Ribosome biogenesis
SG17M	CDS	6386678	6387697	+	rsgA_2	1019	8h	Ribosome biogenesis
NN2	CDS	6186619	6187638	+	rsgA_1	1019	8h	Ribosome biogenesis
SG17M	CDS	1930605	1931930	+	rhlE_6	1325	8h	Ribosome biogenesis
SG17M	CDS	1062293	1063297	+	phzM_2	1004	8h	Secondary metabolite biosynthesis
SG17M	CDS	4363633	4364109	+	yscO_2	476	4h	Secretion protein
NN2	CDS	3938311	3938787	+	yscO_1	476	4h	Secretion protein
NN2	CDS	3938311	3938787	+	yscO_1	476	8h	Secretion protein
SG17M	CDS	2169505	2171253	-	bluF_2	1748	8h	Sensory transduction
SG17M	CDS	2078818	2085876	-	dhbF_2	7058	8h	Siderophore biosynthesis

NN2	CDS	1853646	1860704	-	dhbF_1	7058	8h	Siderophore biosynthesis
SG17M	CDS	2790310	2791335	-	dgcT_5	1025	4h	Single-species biofilm formation
NN2	CDS	2566780	2567805	-	dgcT_2	1025	4h	Single-species biofilm formation
SG17M	CDS	1993620	1994237	+	hasA_2	617	8h	Single-species biofilm formation
NN2	CDS	5205133	5205744	+	hdeD-GI	611	4h	Stress response
SG17M	CDS	6692367	6694706	+	yhgF_2	2339	4h	Stress response
NN2	CDS	6492318	6494657	+	yhgF_1	2339	4h	Stress response
SG17M	CDS	2635889	2636551	-	sspA_3	662	4h	Stress response
NN2	CDS	2410709	2411371	-	sspA_1	662	4h	Stress response
SG17M	CDS	5839292	5841265	+	rapA_7	1973	4h	Stress response
NN2	CDS	5602620	5604593	+	rapA_4	1973	4h	Stress response
SG17M	CDS	249252	250229	-	qorA_4	977	4h	Stress response
SG17M	CDS	125812	126372	-	patZ_2	560	4h	Stress response
SG17M	CDS	1038640	1044069	+	mbtB_4	5429	4h	Stress response
SG17M	CDS	6690021	6691340	-	envZ_4	1319	4h	Stress response
SG17M	CDS	6299922	6301187	+	dnaK_4	1265	4h	Stress response
NN2	CDS	6099863	6101128	+	dnaK_2	1265	4h	Stress response
NN2	CDS	5858057	5859157	+	ychF_1	1100	8h	Stress response
NN2	CDS	161463	162515	+	rihB_1	1052	8h	Stress response
SG17M	CDS	5839292	5841265	+	rapA_7	1973	8h	Stress response
NN2	CDS	5602620	5604593	+	rapA_4	1973	8h	Stress response
NN2	CDS	6804085	6804645	-	patZ_1	560	8h	Stress response
SG17M	CDS	6473534	6474181	+	msrA_2	647	8h	Stress response
NN2	CDS	4986845	4989244	+	lon_2	2399	8h	Stress response
NN2	CDS	3252559	3254103	-	htpX_2	1544	8h	Stress response
SG17M	CDS	6690021	6691340	-	envZ_4	1319	8h	Stress response
SG17M	CDS	6299922	6301187	+	dnaK_4	1265	8h	Stress response

NN2	CDS	6686080	6687765	-	betA_2	1685	8h	Stress response
SG17M	CDS	649087	651366	+	ptsP_2	2279	4h	Sugar phosphotransferase system
NN2	CDS	425074	426666	+	ptsP_1	1592	8h	Sugar phosphotransferase system
SG17M	CDS	6723290	6723901	+	ygfA_2	611	4h	Tetrahydrofolate interconversion
SG17M	CDS	6723290	6723901	+	ygfA_2	611	8h	Tetrahydrofolate interconversion
SG17M	CDS	3336327	3339095	-	rhsC_6	2768	4h	Toxin activity
NN2	CDS	2997603	3000371	-	rhsC_3	2768	4h	Toxin activity
NN2	CDS	2694869	2698822	+	rhsC_2	3953	4h	Toxin activity
NN2	CDS	1807232	1809943	+	rhsC_1	2711	4h	Toxin activity
SG17M	CDS	267598	278130	+	cdiA_2	10532	4h	Toxin activity
NN2	CDS	42903	53435	+	cdiA_1	10532	4h	Toxin activity
NN2	CDS	2997603	3000371	-	rhsC_3	2768	8h	Toxin activity
NN2	CDS	2694869	2698822	+	rhsC_2	3953	8h	Toxin activity
NN2	CDS	1807232	1809943	+	rhsC_1	2711	8h	Toxin activity
NN2	CDS	6383978	6385918	-	estA_1	1940	8h	Toxin activity
SG17M	CDS	5848323	5849903	+	tcpT_2	1580	4h	Toxin coregulated pilus biosynthesis
NN2	CDS	5634713	5636293	+	tcpT_1	1580	4h	Toxin coregulated pilus biosynthesis
SG17M	CDS	5848323	5849903	+	tcpT_2	1580	8h	Toxin coregulated pilus biosynthesis
NN2	CDS	5634713	5636293	+	tcpT_1	1580	8h	Toxin coregulated pilus biosynthesis
SG17M	CDS	5348132	5348833	+	vrr-nuc-domain containing protein	701	4h	Transcription, Transcription regulation, Two-component regulatory system
NN2	CDS	6160799	6162700	+	diguanylate cyclase/phosphodiesterase	1901	4h	Transcription, Transcription regulation, Two-component regulatory system
SG17M	CDS	6360858	6362759	+	diguanylate cyclase/phosphodiesterase	1901	4h	Transcription, Transcription regulation, Two-component regulatory system
SG17M	CDS	2135970	2136485	-	ankrd33	515	4h	Transcription, Transcription regulation, Two-component regulatory system
NN2	CDS	6232857	6233456	+	puuR_2	599	4h	Transcription, Transcription regulation, Two-component regulatory system
SG17M	CDS	3053807	3054961	+	cusS_4	1154	4h	Transcription, Transcription regulation, Two-component regulatory system

SG17M	CDS	3052861	3053553	+	cusR	692	4h	Transcription, Transcription regulation, Two-component regulatory system
SG17M	CDS	1826496	1827134	-	bepR_2	638	4h	Transcription, Transcription regulation, Two-component regulatory system
NN2	CDS	6081933	6084761	+	barA_6	2828	4h	Transcription, Transcription regulation, Two-component regulatory system
SG17M	CDS	5224008	5226785	+	barA_11	2777	4h	Transcription, Transcription regulation, Two-component regulatory system
NN2	CDS	6160799	6162700	+	diguanylate cyclase/phosphodiesterase	1901	8h	Transcription, Transcription regulation, Two-component regulatory system
SG17M	CDS	2336346	2337383	-	araC	1037	8h	Transcription, Transcription regulation, Two-component regulatory system
SG17M	CDS	4784091	4784999	+	pgrR_16	908	8h	Transcription, Transcription regulation, Two-component regulatory system
SG17M	CDS	3161660	3163003	-	hupR1_4	1343	8h	Transcription, Transcription regulation, Two-component regulatory system
SG17M	CDS	1459006	1459902	-	gcvA_13	896	8h	Transcription, Transcription regulation, Two-component regulatory system
SG17M	CDS	3052861	3053553	+	cusR	692	8h	Transcription, Transcription regulation, Two-component regulatory system
SG17M	CDS	80377	81327	+	cmpR_5	950	8h	Transcription, Transcription regulation, Two-component regulatory system
SG17M	CDS	5224008	5226785	+	barA_11	2777	8h	Transcription, Transcription regulation, Two-component regulatory system
NN2	CDS	6474727	6475134	+	transmembrane_protein	407	4h	Transmembrane transporter activity
SG17M	CDS	3296265	3297491	-	yycB_5	1226	4h	Transmembrane transporter activity
NN2	CDS	6515397	6516590	+	ynfM_2	1193	4h	Transmembrane transporter activity
SG17M	CDS	6724736	6725860	-	yhhJ_2	1124	4h	Transmembrane transporter activity
SG17M	CDS	3296265	3297491	-	yycB_5	1226	8h	Transmembrane transporter activity
SG17M	CDS	4410189	4411385	+	ydcO_2	1196	8h	Transmembrane transporter activity
NN2	CDS	884433	886841	-	fpvA_1	2408	8h	Transmembrane transporter activity
SG17M	exon	989564	989648	+	tRNA-TyrGTA	84	4h	tRNA
SG17M	exon	989768	989843	+	tRNA-ThrGGT	75	4h	tRNA
NN2	exon	5577611	5577710	-	tRNA-LysTTT-pseudo	99	4h	tRNA
SG17M	exon	5814283	5814362	-	tRNA-LysTTT-pseudo	79	4h	tRNA
NN2	exon	5704205	5704280	-	tRNA-LysTTT	75	4h	tRNA

SG17M	exon	5917817	5917892	-	tRNA-LysTTT	75	4h	tRNA
SG17M	exon	989675	989748	+	tRNA-GlyTCC	73	4h	tRNA
NN2	exon	764865	764949	+	tRNA-TyrGTA	84	8h	tRNA
NN2	exon	765069	765144	+	tRNA-ThrGGT	75	8h	tRNA
SG17M	exon	989768	989843	+	tRNA-ThrGGT	75	8h	tRNA
NN2	exon	5577611	5577710	-	tRNA-LysTTT-pseudo	99	8h	tRNA
SG17M	exon	5814283	5814362	-	tRNA-LysTTT-pseudo	79	8h	tRNA
NN2	exon	5704205	5704280	-	tRNA-LysTTT	75	8h	tRNA
SG17M	exon	5179331	5179406	-	tRNA-LysTTT	75	8h	tRNA
NN2	exon	6171300	6171386	+	tRNA-LeuCAG	86	8h	tRNA
NN2	exon	764976	765049	+	tRNA-GlyTCC	73	8h	tRNA
SG17M	exon	989675	989748	+	tRNA-GlyTCC	73	8h	tRNA
NN2	CDS	4233066	4233677	-	tRNA-uridine_aminocarboxypropyltransferase	611	4h	tRNA processing
SG17M	CDS	6384905	6385990	+	queG_2	1085	4h	tRNA processing
NN2	CDS	3275900	3277207	-	ttuB_1	1307	8h	tRNA processing
SG17M	CDS	6384905	6385990	+	queG_2	1085	8h	tRNA processing
NN2	CDS	5683932	5684282	+	type_II_toxin-antitoxin_system_RelE/ParE_family_toxin	350	8h	Type II toxin-antitoxin system
NN2	CDS	5717363	5717950	+	PilX	587	4h	Type IV pilus biogenesis
SG17M	CDS	5931574	5935065	+	pilY1_2	3491	8h	Type IV pilus biogenesis
NN2	CDS	5717962	5721453	+	pilY1	3491	8h	Type IV pilus biogenesis
SG17M	CDS	6717536	6718753	-	ubiI_2	1217	8h	Ubiquinone biosynthesis
NN2	CDS	6517487	6518704	-	ubiI_1	1217	8h	Ubiquinone biosynthesis
SG17M	CDS	6864074	6864964	+	ubiA_2	890	8h	Ubiquinone biosynthesis
NN2	CDS	6664024	6664914	+	ubiA_1	890	8h	Ubiquinone biosynthesis
SG17M	CDS	1599587	1601251	-	uncharacterised/unknown	1664	4h	Uncharacterised/Hypothetical/duf
NN2	CDS	706140	707201	-	uncharacterised/unknown	1061	4h	Uncharacterised/Hypothetical/duf
SG17M	CDS	3167160	3169496	-	uncharacterised/unknown	2336	4h	Uncharacterised/Hypothetical/duf

SG17M	CDS	5156755	5157756	-	uncharacterised/unknown	1001	4h	Uncharacterised/Hypothetical/duf
SG17M	CDS	173076	173819	+	uncharacterised/unknown	743	4h	Uncharacterised/Hypothetical/duf
NN2	CDS	8660	10366	+	uncharacterised/unknown	1706	4h	Uncharacterised/Hypothetical/duf
NN2	CDS	73381	74508	+	uncharacterised/unknown	1127	4h	Uncharacterised/Hypothetical/duf
NN2	CDS	162543	163169	-	uncharacterised/unknown	626	4h	Uncharacterised/Hypothetical/duf
NN2	CDS	431343	432140	-	uncharacterised/unknown	797	4h	Uncharacterised/Hypothetical/duf
NN2	CDS	471268	472614	+	uncharacterised/unknown	1346	4h	Uncharacterised/Hypothetical/duf
NN2	CDS	766626	767003	-	uncharacterised/unknown	377	4h	Uncharacterised/Hypothetical/duf
NN2	exon	768760	768952	-	uncharacterised/unknown	192	4h	Uncharacterised/Hypothetical/duf
NN2	CDS	1195031	1195132	-	uncharacterised/unknown	101	4h	Uncharacterised/Hypothetical/duf
NN2	CDS	1328988	1329584	-	uncharacterised/unknown	596	4h	Uncharacterised/Hypothetical/duf
NN2	CDS	1374884	1376548	-	uncharacterised/unknown	1664	4h	Uncharacterised/Hypothetical/duf
NN2	CDS	1598853	1599680	-	uncharacterised/unknown	827	4h	Uncharacterised/Hypothetical/duf
NN2	CDS	1949427	1950077	-	uncharacterised/unknown	650	4h	Uncharacterised/Hypothetical/duf
NN2	CDS	1993452	1993931	-	uncharacterised/unknown	479	4h	Uncharacterised/Hypothetical/duf
NN2	CDS	2630649	2630837	-	uncharacterised/unknown	188	4h	Uncharacterised/Hypothetical/duf
NN2	CDS	2630884	2631804	-	uncharacterised/unknown	920	4h	Uncharacterised/Hypothetical/duf
NN2	CDS	2828437	2830773	-	uncharacterised/unknown	2336	4h	Uncharacterised/Hypothetical/duf
NN2	CDS	2857837	2858151	-	uncharacterised/unknown	314	4h	Uncharacterised/Hypothetical/duf
NN2	CDS	2866620	2866844	-	uncharacterised/unknown	224	4h	Uncharacterised/Hypothetical/duf
NN2	CDS	2871217	2872419	-	uncharacterised/unknown	1202	4h	Uncharacterised/Hypothetical/duf
NN2	CDS	3020203	3020907	-	uncharacterised/unknown	704	4h	Uncharacterised/Hypothetical/duf
NN2	CDS	3211283	3211648	-	uncharacterised/unknown	365	4h	Uncharacterised/Hypothetical/duf
NN2	CDS	3467936	3468916	+	uncharacterised/unknown	980	4h	Uncharacterised/Hypothetical/duf
NN2	CDS	3771482	3771757	+	uncharacterised/unknown	275	4h	Uncharacterised/Hypothetical/duf
NN2	CDS	3790645	3791520	+	uncharacterised/unknown	875	4h	Uncharacterised/Hypothetical/duf
NN2	CDS	3961434	3964961	-	uncharacterised/unknown	3527	4h	Uncharacterised/Hypothetical/duf

NN2	CDS	4896525	4896839	+	uncharacterised/unknown	314	4h	Uncharacterised/Hypothetical/duf
NN2	CDS	4935370	4935888	+	uncharacterised/unknown	518	4h	Uncharacterised/Hypothetical/duf
NN2	CDS	4935885	4936586	+	uncharacterised/unknown	701	4h	Uncharacterised/Hypothetical/duf
NN2	CDS	5067115	5067864	+	uncharacterised/unknown	749	4h	Uncharacterised/Hypothetical/duf
NN2	CDS	5344189	5344548	-	uncharacterised/unknown	359	4h	Uncharacterised/Hypothetical/duf
NN2	CDS	5370194	5370970	+	uncharacterised/unknown	776	4h	Uncharacterised/Hypothetical/duf
NN2	CDS	5584207	5584887	+	uncharacterised/unknown	680	4h	Uncharacterised/Hypothetical/duf
NN2	CDS	5590722	5592053	+	uncharacterised/unknown	1331	4h	Uncharacterised/Hypothetical/duf
NN2	CDS	5606105	5606869	-	uncharacterised/unknown	764	4h	Uncharacterised/Hypothetical/duf
NN2	CDS	5628279	5629043	-	uncharacterised/unknown	764	4h	Uncharacterised/Hypothetical/duf
NN2	CDS	5664407	5665516	+	uncharacterised/unknown	1109	4h	Uncharacterised/Hypothetical/duf
NN2	CDS	5696313	5698331	+	uncharacterised/unknown	2018	4h	Uncharacterised/Hypothetical/duf
NN2	CDS	5997822	5998388	-	uncharacterised/unknown	566	4h	Uncharacterised/Hypothetical/duf
NN2	CDS	6065541	6065672	+	uncharacterised/unknown	131	4h	Uncharacterised/Hypothetical/duf
NN2	CDS	6096317	6097684	+	uncharacterised/unknown	1367	4h	Uncharacterised/Hypothetical/duf
NN2	CDS	6287265	6288080	+	uncharacterised/unknown	815	4h	Uncharacterised/Hypothetical/duf
NN2	CDS	6356191	6356862	-	uncharacterised/unknown	671	4h	Uncharacterised/Hypothetical/duf
NN2	CDS	6356920	6359157	-	uncharacterised/unknown	2237	4h	Uncharacterised/Hypothetical/duf
NN2	CDS	6442131	6443111	+	uncharacterised/unknown	980	4h	Uncharacterised/Hypothetical/duf
NN2	exon	6473815	6474213	-	uncharacterised/unknown	398	4h	Uncharacterised/Hypothetical/duf
NN2	CDS	6476182	6476625	-	uncharacterised/unknown	443	4h	Uncharacterised/Hypothetical/duf
NN2	CDS	6501767	6502444	-	uncharacterised/unknown	677	4h	Uncharacterised/Hypothetical/duf
NN2	CDS	6516629	6517450	+	uncharacterised/unknown	821	4h	Uncharacterised/Hypothetical/duf
NN2	CDS	6524219	6524677	+	uncharacterised/unknown	458	4h	Uncharacterised/Hypothetical/duf
NN2	CDS	6606006	6607934	-	uncharacterised/unknown	1928	4h	Uncharacterised/Hypothetical/duf
NN2	CDS	6664938	6665321	-	uncharacterised/unknown	383	4h	Uncharacterised/Hypothetical/duf
NN2	CDS	6802706	6804079	+	uncharacterised/unknown	1373	4h	Uncharacterised/Hypothetical/duf

NN2	CDS	6806451	6807491	+	uncharacterised/unknown	1040	4h	Uncharacterised/Hypothetical/duf
NN2	CDS	6851349	6852092	+	uncharacterised/unknown	743	4h	Uncharacterised/Hypothetical/duf
NN2	exon	4810150	4810659	+	uncharacterised/unknown	509	4h	Uncharacterised/Hypothetical/duf
NN2	exon	5976006	5976084	+	uncharacterised/unknown	78	4h	Uncharacterised/Hypothetical/duf
NN2	exon	6310557	6310850	-	uncharacterised/unknown	293	4h	Uncharacterised/Hypothetical/duf
SG17M	exon	88069	88160	+	uncharacterised/unknown	91	4h	Uncharacterised/Hypothetical/duf
SG17M	exon	405324	405447	-	uncharacterised/unknown	123	4h	Uncharacterised/Hypothetical/duf
SG17M	CDS	548888	552019	+	uncharacterised/unknown	3131	4h	Uncharacterised/Hypothetical/duf
SG17M	exon	604253	604432	-	uncharacterised/unknown	179	4h	Uncharacterised/Hypothetical/duf
SG17M	CDS	656043	656840	-	uncharacterised/unknown	797	4h	Uncharacterised/Hypothetical/duf
SG17M	CDS	852731	854569	+	uncharacterised/unknown	1838	4h	Uncharacterised/Hypothetical/duf
SG17M	CDS	930841	931902	-	uncharacterised/unknown	1061	4h	Uncharacterised/Hypothetical/duf
SG17M	CDS	945784	945954	-	uncharacterised/unknown	170	4h	Uncharacterised/Hypothetical/duf
SG17M	CDS	991325	991702	-	uncharacterised/unknown	377	4h	Uncharacterised/Hypothetical/duf
SG17M	exon	993459	993651	-	uncharacterised/unknown	192	4h	Uncharacterised/Hypothetical/duf
SG17M	CDS	1823566	1824393	-	uncharacterised/unknown	827	4h	Uncharacterised/Hypothetical/duf
SG17M	CDS	2218623	2219102	-	uncharacterised/unknown	479	4h	Uncharacterised/Hypothetical/duf
SG17M	CDS	2824856	2825413	+	uncharacterised/unknown	557	4h	Uncharacterised/Hypothetical/duf
SG17M	CDS	2854181	2854369	-	uncharacterised/unknown	188	4h	Uncharacterised/Hypothetical/duf
SG17M	CDS	2854416	2855336	-	uncharacterised/unknown	920	4h	Uncharacterised/Hypothetical/duf
SG17M	CDS	3196560	3196874	-	uncharacterised/unknown	314	4h	Uncharacterised/Hypothetical/duf
SG17M	CDS	3358927	3359631	-	uncharacterised/unknown	704	4h	Uncharacterised/Hypothetical/duf
SG17M	CDS	3676771	3677406	-	uncharacterised/unknown	635	4h	Uncharacterised/Hypothetical/duf
SG17M	CDS	3892770	3893750	+	uncharacterised/unknown	980	4h	Uncharacterised/Hypothetical/duf
SG17M	CDS	4386743	4390270	-	uncharacterised/unknown	3527	4h	Uncharacterised/Hypothetical/duf
SG17M	CDS	4519545	4520306	-	uncharacterised/unknown	761	4h	Uncharacterised/Hypothetical/duf
SG17M	CDS	5241834	5242226	+	uncharacterised/unknown	392	4h	Uncharacterised/Hypothetical/duf

SG17M	CDS	5262082	5262342	+	uncharacterised/unknown	260	4h	Uncharacterised/Hypothetical/duf
SG17M	exon	5267608	5268117	+	uncharacterised/unknown	509	4h	Uncharacterised/Hypothetical/duf
SG17M	CDS	5648141	5649109	-	uncharacterised/unknown	968	4h	Uncharacterised/Hypothetical/duf
SG17M	CDS	5827394	5828725	+	uncharacterised/unknown	1331	4h	Uncharacterised/Hypothetical/duf
SG17M	CDS	5828670	5829629	-	uncharacterised/unknown	959	4h	Uncharacterised/Hypothetical/duf
SG17M	CDS	5830782	5831330	+	uncharacterised/unknown	548	4h	Uncharacterised/Hypothetical/duf
SG17M	CDS	5837406	5839295	+	uncharacterised/unknown	1889	4h	Uncharacterised/Hypothetical/duf
SG17M	CDS	5878019	5879128	+	uncharacterised/unknown	1109	4h	Uncharacterised/Hypothetical/duf
SG17M	CDS	6298058	6298759	+	uncharacterised/unknown	701	4h	Uncharacterised/Hypothetical/duf
SG17M	CDS	6766093	6769362	-	uncharacterised/unknown	3269	4h	Uncharacterised/Hypothetical/duf
NN2	CDS	6553299	6553778	+	TIGR02444_family_protein	479	4h	Uncharacterised/Hypothetical/duf
SG17M	CDS	6753347	6753826	+	TIGR02444_family_protein	479	4h	Uncharacterised/Hypothetical/duf
SG17M	CDS	1228177	1228533	-	SirB	356	4h	Uncharacterised/Hypothetical/duf
SG17M	CDS	4692255	4692464	+	regulatory protein	209	4h	Uncharacterised/Hypothetical/duf
NN2	CDS	1254899	1255396	-	rdd domain-containing protein	497	4h	Uncharacterised/Hypothetical/duf
SG17M	CDS	3209942	3211144	-	putative integrase	1202	4h	Uncharacterised/Hypothetical/duf
SG17M	CDS	298076	299203	+	putative aminopeptidase	1127	4h	Uncharacterised/Hypothetical/duf
SG17M	CDS	1383705	1385096	+	PINc domain-containing protein	1391	4h	Uncharacterised/Hypothetical/duf
SG17M	CDS	5831377	5832216	+	phage regulatory protein	839	4h	Uncharacterised/Hypothetical/duf
NN2	CDS	4036406	4037311	+	pbpb_domain-containing_protein	905	4h	Uncharacterised/Hypothetical/duf
SG17M	CDS	2167493	2168239	-	pbpb_domain-containing_protein	746	4h	Uncharacterised/Hypothetical/duf
SG17M	CDS	5175083	5175433	+	parE	350	4h	Uncharacterised/Hypothetical/duf
SG17M	CDS	5347617	5348135	+	paar_domain-containing_protein	518	4h	Uncharacterised/Hypothetical/duf
SG17M	CDS	124433	125806	+	insulinase_family_metalloprotease	1373	4h	Uncharacterised/Hypothetical/duf
NN2	CDS	53669	53782	-	hypothetical_protein	113	4h	Uncharacterised/Hypothetical/duf
NN2	CDS	72379	72816	-	hypothetical_protein	437	4h	Uncharacterised/Hypothetical/duf
NN2	CDS	1598515	1598856	-	hypothetical_protein	341	4h	Uncharacterised/Hypothetical/duf

NN2	CDS	5591998	5592957	-	hypothetical_protein	959	4h	Uncharacterised/Hypothetical/duf
SG17M	CDS	4215965	4216840	+	hydrolase-4 domain-containing protein	875	4h	Uncharacterised/Hypothetical/duf
SG17M	CDS	1664659	1665300	-	hth_tetR-type_domain-containing_protein	641	4h	Uncharacterised/Hypothetical/duf
SG17M	CDS	2174598	2175248	-	had_phosphoserine_phosphatase	650	4h	Uncharacterised/Hypothetical/duf
SG17M	CDS	3128678	3129502	-	GTPase	824	4h	Uncharacterised/Hypothetical/duf
SG17M	CDS	306175	307887	-	fge-domain containing sulfatase	1712	4h	Uncharacterised/Hypothetical/duf
SG17M	CDS	5664916	5665377	+	duf721	461	4h	Uncharacterised/Hypothetical/duf
NN2	CDS	5428409	5428870	+	duf721	461	4h	Uncharacterised/Hypothetical/duf
NN2	CDS	1292986	1293381	-	duf4345	395	4h	Uncharacterised/Hypothetical/duf
SG17M	CDS	5244051	5244566	+	duf4124	515	4h	Uncharacterised/Hypothetical/duf
SG17M	CDS	6600665	6601186	+	duf4124	521	4h	Uncharacterised/Hypothetical/duf
NN2	CDS	6400617	6401138	+	duf4124	521	4h	Uncharacterised/Hypothetical/duf
SG17M	CDS	113113	115071	+	duf4105	1958	4h	Uncharacterised/Hypothetical/duf
NN2	CDS	6791386	6793344	+	duf4105	1958	4h	Uncharacterised/Hypothetical/duf
NN2	CDS	3573194	3573418	+	duf3203	224	4h	Uncharacterised/Hypothetical/duf
NN2	CDS	1081481	1082242	+	duf218	761	4h	Uncharacterised/Hypothetical/duf
SG17M	CDS	2051346	2051957	+	duf2064	611	4h	Uncharacterised/Hypothetical/duf
SG17M	CDS	954693	958307	+	duf1983	3614	4h	Uncharacterised/Hypothetical/duf
NN2	CDS	729992	733606	+	duf1983	3614	4h	Uncharacterised/Hypothetical/duf
NN2	CDS	6774383	6774586	+	duf1127	203	4h	Uncharacterised/Hypothetical/duf
NN2	CDS	416503	416835	-	duf0339	332	4h	Uncharacterised/Hypothetical/duf
NN2	CDS	5953613	5953810	-	duf0337	197	4h	Uncharacterised/Hypothetical/duf
NN2	CDS	5674450	5675106	+	dsba-like thioredoxin domain protein	656	4h	Uncharacterised/Hypothetical/duf
SG17M	CDS	5888062	5888718	+	dsba-like thioredoxin domain protein	656	4h	Uncharacterised/Hypothetical/duf
NN2	CDS	4221642	4223594	+	diguanylate cyclase/phosphodiesterase	1952	4h	Uncharacterised/Hypothetical/duf
NN2	CDS	4639186	4640067	-	yiaD_3	881	4h	Uncharacterised/Hypothetical/duf
SG17M	CDS	5091202	5091666	-	slyB_2	464	4h	Uncharacterised/Hypothetical/duf

NN2	CDS	3957995	3958540	+	folE_2	545	4h	Uncharacterised/Hypothetical/duf
SG17M	CDS	4020711	4021481	+	bdhA_2	770	4h	Uncharacterised/Hypothetical/duf
SG17M	CDS	1834495	1835508	-	zinc-type alcohol dehydrogenase	1013	8h	Uncharacterised/Hypothetical/duf
SG17M	CDS	2204062	2204379	+	uncharacterised/unknown	317	8h	Uncharacterised/Hypothetical/duf
SG17M	CDS	1599587	1601251	-	uncharacterised/unknown	1664	8h	Uncharacterised/Hypothetical/duf
SG17M	CDS	2442253	2442486	+	uncharacterised/unknown	233	8h	Uncharacterised/Hypothetical/duf
SG17M	CDS	6346136	6346831	-	uncharacterised/unknown	695	8h	Uncharacterised/Hypothetical/duf
SG17M	CDS	3167160	3169496	-	uncharacterised/unknown	2336	8h	Uncharacterised/Hypothetical/duf
SG17M	CDS	173076	173819	+	uncharacterised/unknown	743	8h	Uncharacterised/Hypothetical/duf
NN2	CDS	73381	74508	+	uncharacterised/unknown	1127	8h	Uncharacterised/Hypothetical/duf
NN2	CDS	162543	163169	-	uncharacterised/unknown	626	8h	Uncharacterised/Hypothetical/duf
NN2	CDS	283366	284067	-	uncharacterised/unknown	701	8h	Uncharacterised/Hypothetical/duf
NN2	CDS	431343	432140	-	uncharacterised/unknown	797	8h	Uncharacterised/Hypothetical/duf
NN2	CDS	766626	767003	-	uncharacterised/unknown	377	8h	Uncharacterised/Hypothetical/duf
NN2	CDS	1159002	1160393	+	uncharacterised/unknown	1391	8h	Uncharacterised/Hypothetical/duf
NN2	CDS	1439593	1439943	+	uncharacterised/unknown	350	8h	Uncharacterised/Hypothetical/duf
NN2	CDS	1862134	1863021	-	uncharacterised/unknown	887	8h	Uncharacterised/Hypothetical/duf
NN2	CDS	1949427	1950077	-	uncharacterised/unknown	650	8h	Uncharacterised/Hypothetical/duf
NN2	CDS	2588780	2588869	+	uncharacterised/unknown	89	8h	Uncharacterised/Hypothetical/duf
NN2	CDS	2588888	2589064	-	uncharacterised/unknown	176	8h	Uncharacterised/Hypothetical/duf
NN2	CDS	2601326	2601883	+	uncharacterised/unknown	557	8h	Uncharacterised/Hypothetical/duf
NN2	CDS	2828437	2830773	-	uncharacterised/unknown	2336	8h	Uncharacterised/Hypothetical/duf
NN2	CDS	2857837	2858151	-	uncharacterised/unknown	314	8h	Uncharacterised/Hypothetical/duf
NN2	CDS	2871217	2872419	-	uncharacterised/unknown	1202	8h	Uncharacterised/Hypothetical/duf
NN2	CDS	3314674	3315270	-	uncharacterised/unknown	596	8h	Uncharacterised/Hypothetical/duf
NN2	CDS	3343318	3344277	+	uncharacterised/unknown	959	8h	Uncharacterised/Hypothetical/duf
NN2	CDS	3664472	3665611	+	uncharacterised/unknown	1139	8h	Uncharacterised/Hypothetical/duf

NN2	CDS	3707045	3707581	+	uncharacterised/unknown	536	8h	Uncharacterised/Hypothetical/duf
NN2	CDS	3771482	3771757	+	uncharacterised/unknown	275	8h	Uncharacterised/Hypothetical/duf
NN2	CDS	3786567	3786794	+	uncharacterised/unknown	227	8h	Uncharacterised/Hypothetical/duf
NN2	CDS	3790645	3791520	+	uncharacterised/unknown	875	8h	Uncharacterised/Hypothetical/duf
NN2	CDS	4699036	4699257	-	uncharacterised/unknown	221	8h	Uncharacterised/Hypothetical/duf
NN2	CDS	4699296	4700297	-	uncharacterised/unknown	1001	8h	Uncharacterised/Hypothetical/duf
NN2	CDS	4715348	4716859	+	uncharacterised/unknown	1511	8h	Uncharacterised/Hypothetical/duf
NN2	CDS	4717624	4717974	+	uncharacterised/unknown	350	8h	Uncharacterised/Hypothetical/duf
NN2	CDS	4718305	4720224	+	uncharacterised/unknown	1919	8h	Uncharacterised/Hypothetical/duf
NN2	CDS	4923341	4924714	+	uncharacterised/unknown	1373	8h	Uncharacterised/Hypothetical/duf
NN2	CDS	4942236	4942670	+	uncharacterised/unknown	434	8h	Uncharacterised/Hypothetical/duf
NN2	CDS	5044484	5045722	+	uncharacterised/unknown	1238	8h	Uncharacterised/Hypothetical/duf
NN2	CDS	5067115	5067864	+	uncharacterised/unknown	749	8h	Uncharacterised/Hypothetical/duf
NN2	CDS	5584207	5584887	+	uncharacterised/unknown	680	8h	Uncharacterised/Hypothetical/duf
NN2	CDS	5590722	5592053	+	uncharacterised/unknown	1331	8h	Uncharacterised/Hypothetical/duf
NN2	CDS	5600734	5602623	+	uncharacterised/unknown	1889	8h	Uncharacterised/Hypothetical/duf
NN2	CDS	5606105	5606869	-	uncharacterised/unknown	764	8h	Uncharacterised/Hypothetical/duf
NN2	CDS	5643533	5643730	+	uncharacterised/unknown	197	8h	Uncharacterised/Hypothetical/duf
NN2	CDS	5669304	5670809	+	uncharacterised/unknown	1505	8h	Uncharacterised/Hypothetical/duf
NN2	CDS	5671230	5674172	+	uncharacterised/unknown	2942	8h	Uncharacterised/Hypothetical/duf
NN2	CDS	5696313	5698331	+	uncharacterised/unknown	2018	8h	Uncharacterised/Hypothetical/duf
NN2	CDS	5700636	5702555	+	uncharacterised/unknown	1919	8h	Uncharacterised/Hypothetical/duf
NN2	CDS	5832998	5833513	+	uncharacterised/unknown	515	8h	Uncharacterised/Hypothetical/duf
NN2	CDS	5915476	5916363	-	uncharacterised/unknown	887	8h	Uncharacterised/Hypothetical/duf
NN2	CDS	5997822	5998388	-	uncharacterised/unknown	566	8h	Uncharacterised/Hypothetical/duf
NN2	CDS	6102890	6103297	-	uncharacterised/unknown	407	8h	Uncharacterised/Hypothetical/duf
NN2	CDS	6330444	6331373	-	uncharacterised/unknown	929	8h	Uncharacterised/Hypothetical/duf

NN2	CDS	6397721	6398125	+	uncharacterised/unknown	404	8h	Uncharacterised/Hypothetical/duf
NN2	CDS	6476182	6476625	-	uncharacterised/unknown	443	8h	Uncharacterised/Hypothetical/duf
NN2	CDS	6524219	6524677	+	uncharacterised/unknown	458	8h	Uncharacterised/Hypothetical/duf
NN2	CDS	6566045	6569314	-	uncharacterised/unknown	3269	8h	Uncharacterised/Hypothetical/duf
NN2	CDS	6664938	6665321	-	uncharacterised/unknown	383	8h	Uncharacterised/Hypothetical/duf
NN2	CDS	6667694	6669034	+	uncharacterised/unknown	1340	8h	Uncharacterised/Hypothetical/duf
NN2	CDS	6800889	6802637	+	uncharacterised/unknown	1748	8h	Uncharacterised/Hypothetical/duf
NN2	CDS	6851349	6852092	+	uncharacterised/unknown	743	8h	Uncharacterised/Hypothetical/duf
NN2	exon	4810150	4810659	+	uncharacterised/unknown	509	8h	Uncharacterised/Hypothetical/duf
NN2	exon	5976006	5976084	+	uncharacterised/unknown	78	8h	Uncharacterised/Hypothetical/duf
SG17M	CDS	695968	697314	+	uncharacterised/unknown	1346	8h	Uncharacterised/Hypothetical/duf
SG17M	CDS	930841	931902	-	uncharacterised/unknown	1061	8h	Uncharacterised/Hypothetical/duf
SG17M	CDS	945784	945954	-	uncharacterised/unknown	170	8h	Uncharacterised/Hypothetical/duf
SG17M	CDS	958736	958876	-	uncharacterised/unknown	140	8h	Uncharacterised/Hypothetical/duf
SG17M	CDS	991325	991702	-	uncharacterised/unknown	377	8h	Uncharacterised/Hypothetical/duf
SG17M	exon	993459	993651	-	uncharacterised/unknown	192	8h	Uncharacterised/Hypothetical/duf
SG17M	CDS	1275937	1276233	+	uncharacterised/unknown	296	8h	Uncharacterised/Hypothetical/duf
SG17M	CDS	1549776	1552700	+	uncharacterised/unknown	2924	8h	Uncharacterised/Hypothetical/duf
SG17M	CDS	1823566	1824393	-	uncharacterised/unknown	827	8h	Uncharacterised/Hypothetical/duf
SG17M	CDS	1986860	1987258	-	uncharacterised/unknown	398	8h	Uncharacterised/Hypothetical/duf
SG17M	CDS	1987303	1987887	-	uncharacterised/unknown	584	8h	Uncharacterised/Hypothetical/duf
SG17M	CDS	2218623	2219102	-	uncharacterised/unknown	479	8h	Uncharacterised/Hypothetical/duf
SG17M	CDS	2703596	2704345	-	uncharacterised/unknown	749	8h	Uncharacterised/Hypothetical/duf
SG17M	CDS	2726147	2726860	-	uncharacterised/unknown	713	8h	Uncharacterised/Hypothetical/duf
SG17M	CDS	2812310	2812399	+	uncharacterised/unknown	89	8h	Uncharacterised/Hypothetical/duf
SG17M	CDS	2824856	2825413	+	uncharacterised/unknown	557	8h	Uncharacterised/Hypothetical/duf
SG17M	CDS	2854181	2854369	-	uncharacterised/unknown	188	8h	Uncharacterised/Hypothetical/duf

SG17M	CDS	2854416	2855336	-	uncharacterised/unknown	920	8h	Uncharacterised/Hypothetical/duf
SG17M	CDS	3108185	3108934	-	uncharacterised/unknown	749	8h	Uncharacterised/Hypothetical/duf
SG17M	CDS	3205345	3205569	-	uncharacterised/unknown	224	8h	Uncharacterised/Hypothetical/duf
SG17M	CDS	3293239	3294492	-	uncharacterised/unknown	1253	8h	Uncharacterised/Hypothetical/duf
SG17M	CDS	3358927	3359631	-	uncharacterised/unknown	704	8h	Uncharacterised/Hypothetical/duf
SG17M	CDS	3676771	3677406	-	uncharacterised/unknown	635	8h	Uncharacterised/Hypothetical/duf
SG17M	CDS	4519545	4520306	-	uncharacterised/unknown	761	8h	Uncharacterised/Hypothetical/duf
SG17M	CDS	4521576	4521890	-	uncharacterised/unknown	314	8h	Uncharacterised/Hypothetical/duf
SG17M	CDS	5210027	5210218	+	uncharacterised/unknown	191	8h	Uncharacterised/Hypothetical/duf
SG17M	CDS	5232541	5232900	-	uncharacterised/unknown	359	8h	Uncharacterised/Hypothetical/duf
SG17M	exon	5267608	5268117	+	uncharacterised/unknown	509	8h	Uncharacterised/Hypothetical/duf
SG17M	CDS	5837406	5839295	+	uncharacterised/unknown	1889	8h	Uncharacterised/Hypothetical/duf
SG17M	CDS	5878019	5879128	+	uncharacterised/unknown	1109	8h	Uncharacterised/Hypothetical/duf
SG17M	CDS	6265598	6265729	+	uncharacterised/unknown	131	8h	Uncharacterised/Hypothetical/duf
SG17M	CDS	6487314	6488129	+	uncharacterised/unknown	815	8h	Uncharacterised/Hypothetical/duf
SG17M	CDS	6706599	6706928	+	uncharacterised/unknown	329	8h	Uncharacterised/Hypothetical/duf
SG17M	CDS	3206152	3206814	-	uncharacterised/unknown	662	8h	Uncharacterised/Hypothetical/duf
NN2	CDS	6553299	6553778	+	TIGR02444_family_protein	479	8h	Uncharacterised/Hypothetical/duf
SG17M	CDS	6753347	6753826	+	TIGR02444_family_protein	479	8h	Uncharacterised/Hypothetical/duf
SG17M	CDS	3083353	3085218	-	relaxase	1865	8h	Uncharacterised/Hypothetical/duf
SG17M	CDS	4692255	4692464	+	regulatory protein	209	8h	Uncharacterised/Hypothetical/duf
SG17M	CDS	3209942	3211144	-	putative integrase	1202	8h	Uncharacterised/Hypothetical/duf
SG17M	CDS	5368367	5370688	+	putative helicase	2321	8h	Uncharacterised/Hypothetical/duf
SG17M	CDS	2066722	2068908	-	pnpla domain-containing protein	2186	8h	Uncharacterised/Hypothetical/duf
SG17M	CDS	6530493	6531422	-	phasin family protein	929	8h	Uncharacterised/Hypothetical/duf
SG17M	CDS	2167493	2168239	-	pbpb_domain-containing_protein	746	8h	Uncharacterised/Hypothetical/duf
SG17M	CDS	5347617	5348135	+	paar_domain-containing_protein	518	8h	Uncharacterised/Hypothetical/duf

SG17M	CDS	5145851	5146849	+	mvfR	998	8h	Uncharacterised/Hypothetical/duf
SG17M	CDS	5357742	5358989	+	mfs domain-containing protein	1247	8h	Uncharacterised/Hypothetical/duf
SG17M	CDS	2536936	2537193	-	membrane protein	257	8h	Uncharacterised/Hypothetical/duf
NN2	CDS	2022249	2022626	+	hypothetical_protein	377	8h	Uncharacterised/Hypothetical/duf
SG17M	CDS	4215965	4216840	+	hydrolase-4 domain-containing protein	875	8h	Uncharacterised/Hypothetical/duf
SG17M	CDS	3695735	3697054	-	gluconate_2-dehydrogenase_cytochrome_c	1319	8h	Uncharacterised/Hypothetical/duf
SG17M	CDS	2894815	2895210	+	gfa_family_protein	395	8h	Uncharacterised/Hypothetical/duf
SG17M	CDS	5568511	5569512	+	flippase-like domain-containing protein	1001	8h	Uncharacterised/Hypothetical/duf
NN2	CDS	5428409	5428870	+	duf721	461	8h	Uncharacterised/Hypothetical/duf
SG17M	CDS	5244051	5244566	+	duf4124	515	8h	Uncharacterised/Hypothetical/duf
NN2	CDS	6400617	6401138	+	duf4124	521	8h	Uncharacterised/Hypothetical/duf
SG17M	CDS	6867744	6869084	+	duf3971	1340	8h	Uncharacterised/Hypothetical/duf
NN2	CDS	5642684	5643163	+	duf3577	479	8h	Uncharacterised/Hypothetical/duf
SG17M	CDS	4211887	4212114	+	duf2970	227	8h	Uncharacterised/Hypothetical/duf
SG17M	CDS	354378	354587	-	duf2909	209	8h	Uncharacterised/Hypothetical/duf
SG17M	CDS	2132796	2134931	+	duf2235	2135	8h	Uncharacterised/Hypothetical/duf
SG17M	CDS	5018993	5019214	+	duf2132	221	8h	Uncharacterised/Hypothetical/duf
NN2	CDS	4561534	4561755	+	duf2132	221	8h	Uncharacterised/Hypothetical/duf
SG17M	CDS	2051346	2051957	+	duf2064	611	8h	Uncharacterised/Hypothetical/duf
SG17M	CDS	954693	958307	+	duf1983	3614	8h	Uncharacterised/Hypothetical/duf
SG17M	CDS	96110	96313	+	duf1127	203	8h	Uncharacterised/Hypothetical/duf
NN2	CDS	6774383	6774586	+	duf1127	203	8h	Uncharacterised/Hypothetical/duf
SG17M	CDS	5729061	5729585	+	duf0307	524	8h	Uncharacterised/Hypothetical/duf
NN2	CDS	4348049	4348498	-	duf0260	449	8h	Uncharacterised/Hypothetical/duf
SG17M	CDS	5888062	5888718	+	dsba-like thioredoxin domain protein	656	8h	Uncharacterised/Hypothetical/duf
SG17M	CDS	4689426	4691378	+	diguanylate cyclase/phosphodiesterase	1952	8h	Uncharacterised/Hypothetical/duf
NN2	CDS	3179899	3180831	-	scrK_1	932	8h	Uncharacterised/Hypothetical/duf

NN2	CDS	6525815	6528565	-	rbbA_1	2750	8h	Uncharacterised/Hypothetical/duf
SG17M	CDS	5610765	5611817	+	nemA_6	1052	8h	Uncharacterised/Hypothetical/duf
NN2	CDS	5374256	5375308	+	nemA_3	1052	8h	Uncharacterised/Hypothetical/duf
SG17M	CDS	4817688	4818620	-	ghrA_2	932	8h	Uncharacterised/Hypothetical/duf
NN2	CDS	4349901	4350833	-	ghrA_1	932	8h	Uncharacterised/Hypothetical/duf
SG17M	CDS	2776676	2777914	-	cpg2_2	1238	8h	Uncharacterised/Hypothetical/duf
NN2	CDS	1443424	1445130	+	urea amidohydrolase subunit_alpha	1706	4h	urea catabolic process
SG17M	CDS	1668139	1669845	+	urea amidohydrolase subunit alpha	1706	4h	urea catabolic process
NN2	CDS	6359154	6361526	-	vgrG1_7	2372	8h	Virulence
NN2	CDS	4689343	4689945	-	phnB_1	602	8h	Virulence
